# Synthesis and Reactivity of a Mono‐Coordinated Triplet Bismuthinidene

**DOI:** 10.1002/anie.202508250

**Published:** 2025-07-11

**Authors:** Yannick Schulte, Timo Freese, Christoph Wölper, Jan Schulte, Gebhard Haberhauer, Stephan Schulz

**Affiliations:** ^1^ Institute of Inorganic Chemistry University of Duisburg‐Essen Universitätsstraße 5–7 Essen D‐45141 Germany; ^2^ Institute of Organic Chemistry University of Duisburg‐Essen Universitätsstraße 5–7 Essen D‐45141 Germany; ^3^ Center for Nanointegration Duisburg‐Essen (CENIDE) University of Duisburg‐Essen Carl‐Benz‐Straße 199 Duisburg D‐47057 Germany

**Keywords:** Bismuth hydride, Bismuthinidene, DFT calculation, Sc‐XRD

## Abstract

The triplet bismuthinidene Ar*Bi (**4**) stabilized by a very bulky septiphenyl ligand (Ar* = 3,5‐*i*‐Pr_2_‐2,6‐(2,6‐Me_2_‐3,5‐(2,6‐*i*‐Pr_2_C_6_H_3_)_2_–C_6_H)–C_6_H) was synthesized by dehydrogenation of in situ formed bismuth dihydride Ar*BiH_2_ (**3**). Oxidative addition reactions of **4** with alkyl halides (MeI, EtBr, *i*‐PrBr) yielded bismuthanes Ar*Bi(Me)I (**5**), Ar*Bi(Et)Br (**6**), and Ar*Bi(*i*‐Pr)Br (**7**), which reacted with LiAlH_4_ and LiAlD_4_ to the thermally robust bismuth monohydrides Ar*Bi(R)H (R = Me **8**, Et **10**, *i*‐Pr **12**) and monodeuterides Ar*Bi(R)D (R = Me **9**, Et **11**, *i*‐Pr **13**). Ar*Bi(NMe_2_)_2_ **1** and Ar*BiH_2_ **3** were characterized in situ by ^1^H NMR spectroscopy and sc‐XRD (**1**), whereas the other compounds were characterized by heteronuclear NMR (^1^H/^2^H (D), ^13^C) and IR spectroscopy, elemental analysis (Ar*‐2, Ar*‐3, Ar*‐5, Ar*‐7, Ar*I, Ar*H, **2**, **4**−**7**), as well as by UV–vis (**4**) and sc‐XRD (Ar*‐7, Ar*I, Ar*Li·Li*t*‐Bu, Ar*H, **1**, **2**, **4**, **12**). Quantum chemical calculations revealed the triplet character of the bismuthinidine **4**.

## Graphical abstract

Dehydrogenation of Ar*BiH_2_ **3** yielded the kinetically stabilized mono‐coordinated triplet bismuthinidene **4**, which was structurally characterized by sc‐XRD and whose electronic character was analyzed by quantum chemical calculations. Bismuthinidene **4** reacts with alkyl halides with oxidative addition to bismuthanes Ar*Bi(R)X, which react with LiAlH_4_ to the corresponding room temperature‐stable bismuthanes Ar*Bi(R)H.

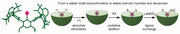

## Introduction

Unsupported pnictinidenes RPn (Pn = N–Bi) containing a monocoordinated pnictogen atom in the formal oxidation state +1, which are isoelectronic to tetrylenes R_2_M (M = C−Pb), are electron‐deficient six‐valence electron species. They are typically highly reactive, short‐lived species, which makes their isolation and structural characterization very challenging.^[^ ^1^ ^]^ For instance, the life span of nitrenes RN, which are involved as reaction intermediates in many organic reactions, is typically in the microsecond range.^[^ ^2^ ^]^

**Figure 1:**
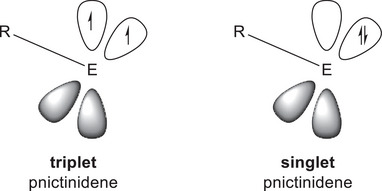
Electronic structure of triplet and singlet pnictinidenes. The heaviest pnictinidenes (E = As, Sb, Bi) each contain an energetically low‐lying s‐orbital, which is not presented, while the electron lone pair of the lighter homologues (N, P) shows substantial p‐orbital contributions due to a more effective hybridization (also not presented).

Pnictinidenes RPn can adopt two different electronic states: In the closed‐shell singlet state, they contain electron lone pairs in the (at least for the heavier pnictinidenes) energetically low‐lying s‐orbital and in one p‐orbital, leaving one p‐orbital empty, whereas in the triplet state, they exist as single‐centered diradicals with one electron lone pair in the s‐orbital and two unpaired electrons in the p‐orbitals (Figure 1).

Nitrenes typically exist in the triplet ground state. The same is true for most heavier pnictinidenes as was recently demonstrated by Schaefer III et al. They calculated singlet‐triplet energy differences (Δ*E*_ST_ = *E*_Singlet _− *E*_Triplet_) of a series of heavy pnictinidenes (RPn; Pn = P, As, Sb, Bi) and identified only P−PH_2_ (−3.2 kcal mol^−1^) and P−NH_2_ (−0.2 kcal mol^−1^) as pnictinidenes with favorable singlet states.^[^ ^3^ ^]^

**Scheme 1:**
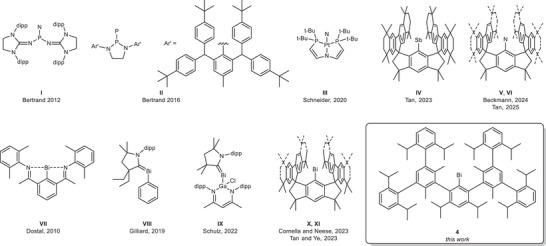
Examples of isolated pnictinidenes.

Two general approaches have been established for the isolation of pnictinidenes RPn:^[^ ^4^ ^]^ Singlet pnictinidenes are accessible via *electronic stabilization* using a suitable donor ligand that compensates the electron deficiency at the pnictogen center as was revealed by quantum chemical calculations, whereas the use of sterically demanding aryl substituents stabilizes the triplet ground state.^[^ ^3^ ^]^ This strategy allowed for the isolation and structural characterization of phosphino‐substituted singlet nitrene **I** ^[^ ^5^ ^]^ and phosphinidene **II**,^[^ ^6^ ^]^ whereas the structural characterization of the unstable Pt‐coordinated nitrene **III** was only possible by in situ generation on the diffractometer (Scheme 1).^[^ ^7^ ^]^

In addition, room temperature‐stable triplet pnictinidenes have recently been synthesized by use of sterically demanding hydrindacene (M^s^FluInd) ligands. Tan et al. reported on the single crystal X‐ray structure (sc‐XRD) of the NMR‐silent stibinidene M^s^Fluind*Sb **IV**,^[^ ^8^ ^]^ which showed an effective magnetic moment of 1.96 *μ*_B_ at 293 K according to Evan's method, while the same group and Beckmann et al. very recently structurally characterized two room temperature‐stable triplet nitrenes, M^s^FluIndN **V** and M^s^FluInd*N **VI**, which were synthesized by photolysis of the corresponding arylazides.^[^ ^9^, ^10^, ^11^ ^]^

The synthesis of bismuthinidenes RBi is also very challenging since they exhibit the weakest Bi─R bond energy within the group. While room temperature‐stable intra‐ and intermolecular donor‐stabilized species are known for some time (**VII**, **VIII**, **IX**),^[^ ^12^, ^13^, ^14^ ^]^ an unsupported bismuthinidene, BiMe, was accessed by Lichtenberg et al. only recently via controlled thermal homolysis in the gas phase. It shows a triplet (biradical) ground state according to quantum chemical computations^[^ ^15^ ^]^ as is also true for the parent bismuthinidene, BiH, which can also be generated in the gas phase.^[^ ^16^, ^17^ ^]^ In 2023, the groups of Tan et al. and Cornella et al. independently reported on the isolation and single crystal structures of two bismuthinidenes stabilized by sterically demanding hydrindacene ligands M^s^FluInd^tBu^ and M^s^FluInd* (**X**, **XI**),^[^ ^18^, ^19^ ^]^ respectively. Both compounds showed diamagnetism in EPR and SQUID measurements, but quantum chemical calculations revealed that the ground state in these compounds is dominated by spin triplets (inert 6s electron lone pair and two unpaired valence electrons in two 6p orbitals) and the diamagnetic nature was explained by very large SOC‐induced positive zero‐field splitting (>4300 wavenumbers) in their S = 1 ground states, which leads in the population of the *M*_s_ = 0 level at room temperature that renders them diamagnetic. This extremely large ZFS has been recently confirmed by Said et al.^[^ ^20^ ^]^

While a few bismuthinidenes with a coordination number of one are now known, their chemical reactivity is still rather unexplored. Triplet bismuthinidenes were reported to react with insertion into the *σ* bonds of methyl iodide and bibenzyl as well as to form Fe(CO)_5_ and Cr(CO)_5_ complexes.^[^ ^18^, ^19^ ^]^ In addition, the protolysis of a triplet bismuthinidene has also been reported, resulting in the formation of a novel tribismaallyl cation.^[^ ^21^ ^]^

We are generally interested in low‐valent organobismuth compounds and their reactivity. Since terphenyl ligands have been used in the past for the stabilization of main group metal and transition metal compounds with unusual electronic properties and structures,^[^ ^22^ ^]^ i.e., the stabilization of the first Cr─Cr quintuple bond,^[^ ^23^ ^]^ the first alanediyl with monocoordinate Al(I) atom,^[^ ^24^ ^]^ and the only room temperature stable bismuth hydride,^[^ ^25^ ^]^ respectively, we started to investigate its potential use for the stabilization of mono‐coordinate bismuthinidenes. We here report on the synthesis and reactivity of a terphenyl‐substituted bismuthinidene Ar*Bi **4** (Ar* = 3,5‐*i*‐Pr_2_‐2,6‐(2,6‐Me_2_‐3,5‐(2,6‐*i*‐Pr_2_C_6_H_3_)_2_–C_6_H)–C_6_H).

## Results and Discussion

**Scheme 2:**
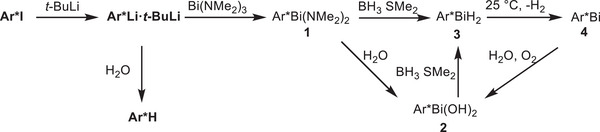
Synthesis of bismuthinidene Ar*Bi (**4**) via Ar*Bi(NMe_2_)_2_ (**1**) and Ar*BiH_2_ (**3**) and reoxidation of **4** to dihydroxide Ar*Bi(OH)_2_ (**2**).

The very bulky, rigid, and chemically inert septiphenyl iodide Ar*I was synthesized in a multistep reaction sequence (Figure S1) including sequential additions of organomagnesium or ‐lithium reagents to aryne intermediates as was previously described for related ligands.^[^ ^26^, ^27^, ^28^ ^]^ Ar*I was obtained in moderate overall yield of 20% based on Ar*‐6 in five synthetic steps from commercially available starting materials (Ar*‐1, Ar*‐4, Ar*‐6) and was purified by recrystallization from toluene. The lithiation of Ar*I turned out to be rather difficult. While the reaction of Ar*I with *n*‐BuLi in benzene at 25 °C was very slow, the addition of Et_2_O or THF resulted in slow (a few hours) or immediate decomposition reactions, respectively. In contrast, the reaction with *t*‐BuLi in benzene or toluene at ambient temperature yielded Ar*Li·Li*t*‐Bu in 75% isolated yield together with small amounts of Ar*H despite the use of carefully dried and purified solvents (Scheme 2). Single crystals containing Ar*Li·Li*t*‐Bu and approx. 15% of Ar*H as well as 2 equiv of toluene were obtained from toluene solution.

The reaction of Ar*Li·Li*t*‐Bu with an excess of BiCl_3_ only resulted in slow formation of Ar*H, whereas the reaction with Bi(NMe_2_)_3_ at 25 °C cleanly gave the corresponding bisamidobismuthane **1**. Bismuthane **1** is sensitive to heat and light and decomposes in solution at ambient temperature within minutes and was therefore only characterized by ^1^H NMR spectroscopy. In addition, storage of a saturated solution of **1** in C_6_D_6_ at 6 °C resulted in the formation of yellow crystals of **1** suitable for sc‐XRD. Bisamidobismuthane **1** crystallizes in the space group *P*2_1_/c with four molecules in the unit cell. The bond angles around the bismuth atom and the Bi─C bond distance are rather large (N1─Bi1─N2 99.82(15)°, N1─Bi1─C1 97.34(14)°, N2─Bi1─C1 108.14(13)°, Bi1─C1 2.309(4) Å), most likely due to the sterically encumbered environment around the bismuth atom. However, considering the low quality of the data, results beyond the connectivity should be carefully assessed.

Reactivity studies of **1** were performed with samples of **1** generated in situ at 25 °C. Hydrolysis of a solution of **1** in toluene yielded the bishydroxobismuthane **2**, which is thermally stable and moderately light‐sensitive but not sensitive to air and moisture. Bishydroxobismuthane **2** was spectroscopically characterized (^1^H, ^13^C NMR, IR) and by sc‐XRD.^[^ ^29^ ^]^ **2** crystallizes in the space group *P*2_1_/*m* with two molecules in the unit cell. Unfortunately, the oxygen atoms are strongly disordered and restraints had to be applied for refinement, hence a detailed structural discussion is not possible. The Bi─C bond distance is shorter than in **1** (2.054(7) Å), in accordance with the lower steric strain.

**Figure 2:**
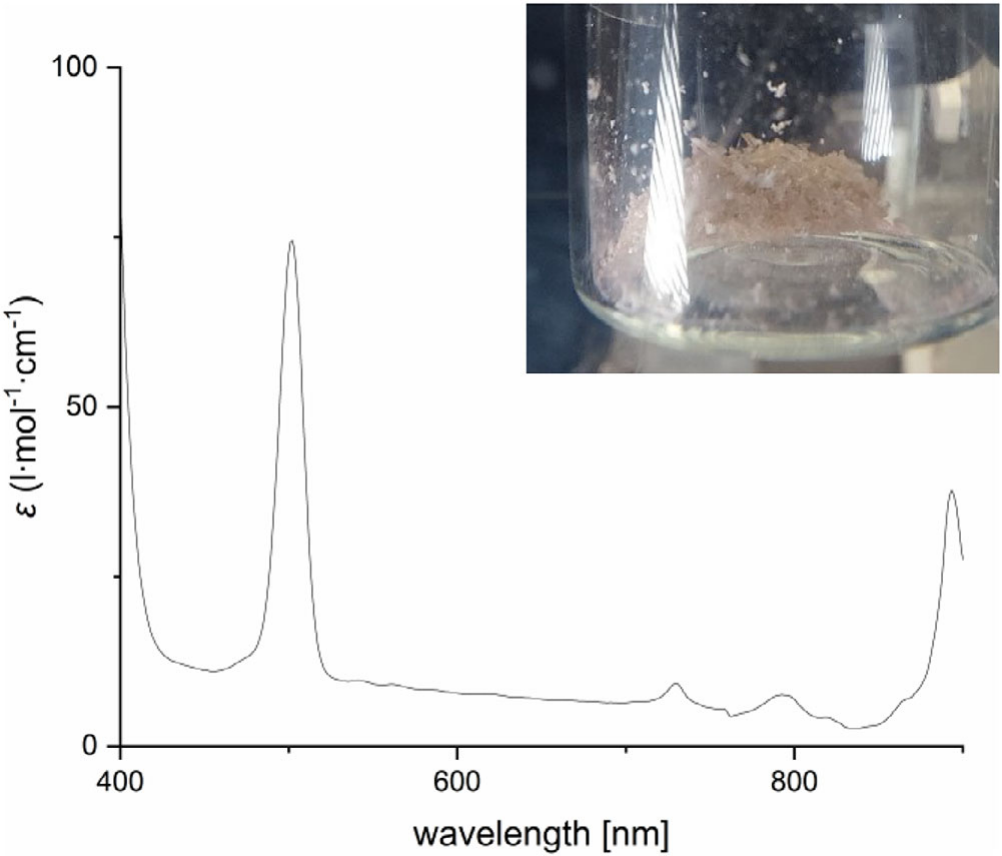
UV–vis spectrum (1 mM, toluene, 25 °C) with maxima of absorption bands at 502 and 893 nm and photo of bismuthinidene **4** (cf. Figure S35 for complete spectrum).

Both **1** and **2** were found to react with BH_3_∙SMe_2_ to the dihydride Ar*BiH_2_ **3**, whose formation was identified by in situ ^1^H NMR spectroscopy at −40 °C (Figure S27) due to the appearance of a resonance at 9.67 ppm for the Bi─H unit. As‐prepared yellow solutions of **3** decomposed upon raising the temperature to 25 °C with evolution of dihydrogen, resulting in the formation of bismuthinidene Ar*Bi **4**. Bismuthinidene **4** was isolated as an almost colorless to light pink crystalline solid (Figure 2), which is soluble in common organic solvents. **4** is moderately light‐sensitive and should be stored in the dark, but short handling (some minutes) can be performed under normal laboratory conditions. The solution of **4** in C_6_D_6_ is diamagnetic at ambient temperature according to Evan's method, and the ^1^H NMR spectrum shows sharp resonances with unusual chemical shifts as was also observed for **X** and **XI**.^[^ ^18^, ^19^ ^]^ For instance, the resonance of the *para*‐H atom appears at −1.41 ppm and the ^13^C resonance of the Bi─C atom was found at −194.7 ppm (Figures S30 and S32), respectively. The UV–vis spectrum of **4** showed maxima of absorption bands at 502 and 893 nm (Figure 2), which is in accordance with those reported for **X** by Cornella et al. (490 and 1011 nm).^[^ ^19^ ^]^

**Figure 3:**
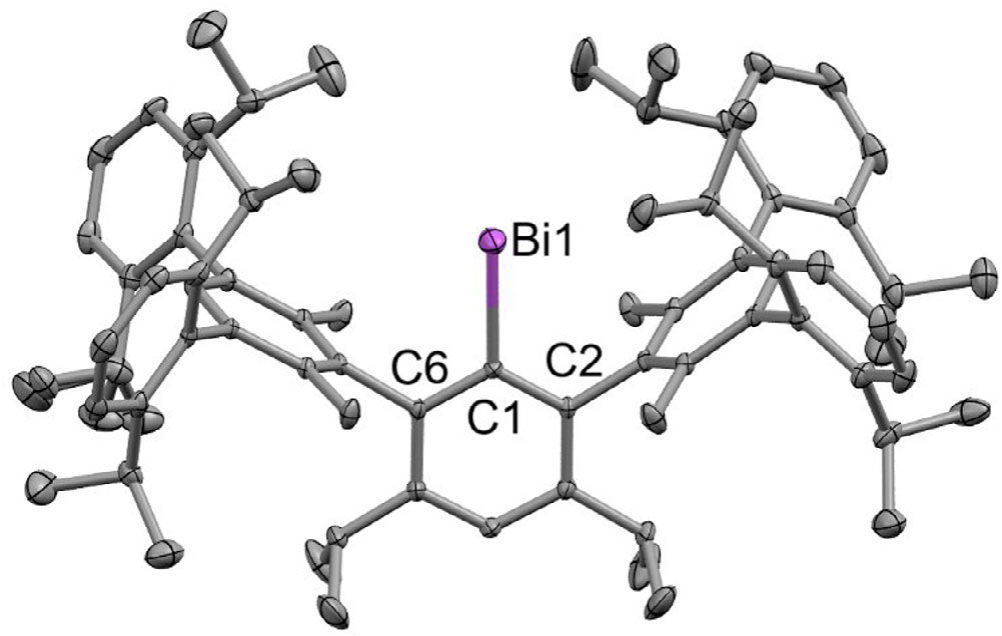
Molecular structure of bismuthinidene **4** in the crystalline state. The bismuth atom is depicted in magenta and carbon atoms in gray. Hydrogen atoms and a solvent molecule (hexafluorobenzene) are omitted for clarity. Ellipsoids are drawn at a probability of 50%.

Bismuthinidene **4** crystallizes in the space group *P*‐1 with two molecules in the unit cell and shows a mono‐coordinated Bi atom (Figure 3) as was observed for the hydrindacene‐substituted bismuthinidenes **X** and **XI**, respectively.

All structurally characterized bismuthinidenes show very similar Bi─C bond lengths (cf. Table 1), and the bond angles around the bismuth‐substituted carbon atom (C1) are all nearly equal to 120°, indicating an approximate triangular planar coordination environment. These findings are not surprising since the calculated buried volumes^[^ ^30^ ^]^ demonstrate that the steric demand of the septiphenyl ligand used in this study is approximately the same as that of M^s^FluInd* but higher than that of M^s^FluInd^tBu^ (cf. Table 1).

**Table 1:** Selected bond lengths and angles of **4**, **X**, and **XI**, as well as buried volumes of the corresponding ligands.

	Ar*Bi **4**	M^s^FluInd^tBu^Bi **X** ^a)^	M^s^FluInd*Bi **XI**
Distance Bi─C1 (A)	2.270(3)	2.2783(10)	2.276(5)
Bi─C1─C (°)	118.8(2), 120.8(2)	121.41(7), 122.22(7)	121.8(3), 122.1(3)
C─C1─C (°)	120.4(2)	116.37(9)	116.1(4)
Sum of angles (C1) (°)	360	360	360
Buried volume (%)	74.6	69.0	74.7

^a)^ Both Tan et al.^[^ ^18^ ^]^ and Cornella et al.^[^ ^19^ ^]^ reported the crystal structure of the same polymorph of **X**, but the resolution of the structure reported by Cornella et al. is higher.

By means of quantum chemical calculations, we wanted to get an insight into the electronic structure of bismuthinidene **4**. As mentioned at the beginning, a bismuthinidene can exist in the electronic triplet or singlet state. In the latter, a distinction can be made between open‐ and closed‐shell states. To examine the electronic configuration of **4**, geometry optimizations were performed by means of density functional theory (DFT). The functional B3LYP^[^ ^31^, ^32^, ^33^ ^]^ with the dispersion correction D3BJ^[^ ^34^ ^]^ was employed (see Supporting Information). Three structures were computed, assuming a closed‐shell singlet ground state, a triplet ground state, and an open‐shell singlet ground state. For the latter, the open‐shell variant UDFT was used as implemented in Gaussian. With this method, we have already been able to describe a multitude of diradical singlet systems well.^[^ ^35^, ^36^, ^37^, ^38^, ^39^ ^]^ The energy values obtained with this method were in excellent agreement with those determined experimentally.^[^ ^35^, ^36^, ^37^, ^38^, ^39^ ^]^ The triplet state showed the lowest energy followed by the open‐shell (+4.6 kcal mol^−1^) and the closed‐shell singlet state (+11.2 kcal mol^−1^). These values are similar to those for bismuthinidene **X**.^[^ ^19^ ^]^ The triplet and open‐shell singlet structures of **4** show similar lengths for the Bi─C bond (2.273 and 2.264 Å) to that found experimentally (2.270(3) Å), whereas a shorter Bi─C bond length (2.225 Å) is found for the closed‐shell system.

**Figure 4:**
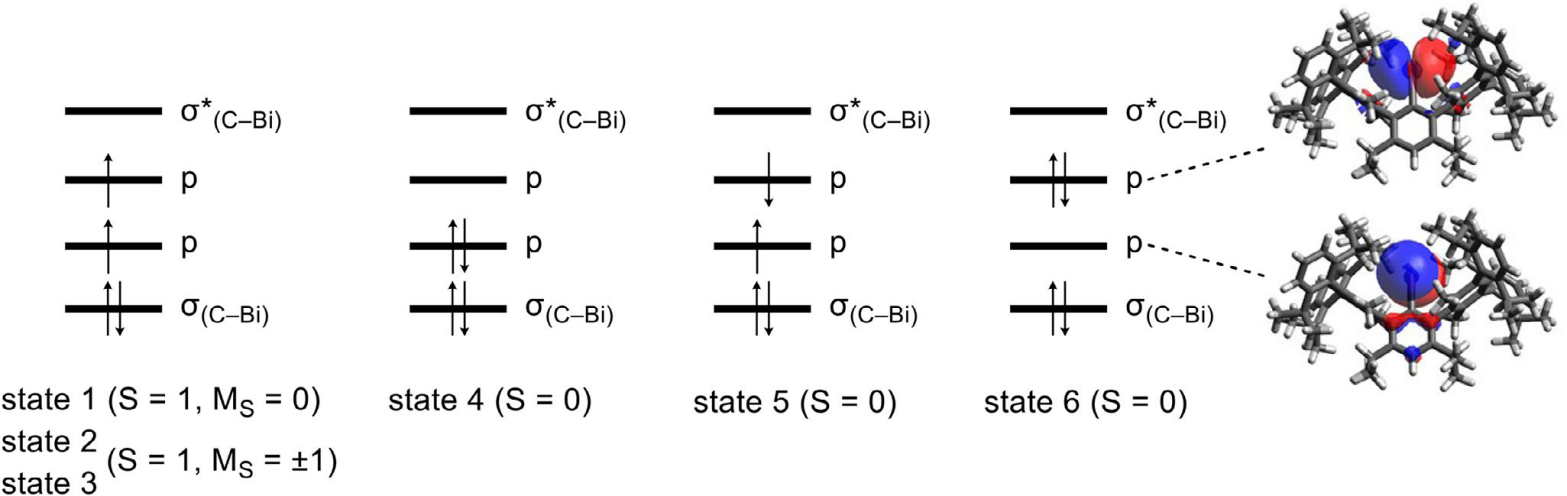
Electronic configuration of the lowest states of **4** calculated by means of NEVPT2. The two nonbonding 6p orbitals at the Bi atom are illustrated on the right side.

The electronic structure was subsequently determined on the DFT‐optimized geometry using relativistic multiconfigurational ab initio methods to consider the effects of spin‐orbit coupling (SOC). In analogy to system **X**,^[^ ^19^ ^]^ a scalar relativistic complete active space self‐consistent field (CASSCF) calculation was performed. The thus obtained orbitals were used as starting orbitals for the *n*‐electron valence second‐order perturbation theory (NEVPT2^[^ ^40^ ^]^), which covers dynamic correlation. The active space included four electrons and four orbitals. The latter are the two non‐bonding 6p orbitals of Bi as well as the *σ* and σ* bonding orbitals of the Bi─C bond (Figure S80). In these calculations, the scalar relativistic states (six singlets and five triplets) were allowed to interact through the SOC. As with bismuthinidene **X**, the ground state is dominated by the spin triplet state (S = 1 and M_S_ = 0) (weight: 0.77), in which one electron is found in each of the non‐bonding 6p orbitals (Figure 4). The two closed‐shell singlet states, in which two electrons are localized in one of the two 6p orbitals, have a total weight of 0.21. The SOC‐induced splitting into the magnetic sublevels is so large that the triplet states with the components M_S_ = ±1 are more than 4100 cm^−1^ higher in energy (4144 and 4442 cm^−1^). Both states have an almost complete triplet character.

Based on the results from the relativistic CASSCF and NEVPT2 calculations (Table S1 and Figure S81), the bands in the UV–vis spectrum can also be determined. The band at 893 nm corresponds to the transitions from the ground state (state 1: S = 1, M_S_ = 0) to the states 4 and 5, which are almost pure singlet states (Figure 4). The leading configurations of the latter are closed‐shell singlet (1023 nm) and open‐shell singlet (1017 nm; see Figure 4). The band at 502 nm can be assigned to a transition from the triplet ground state (state 1) to a closed‐shell singlet (state 6). This transition is calculated at 487 nm, which is in agreement with experiment.

**Scheme 3:**
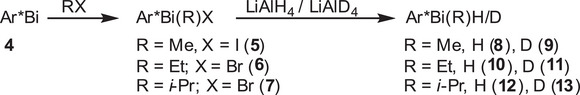
Oxidative addition reactions of bismuthinidene 4 with alkyl halides to compounds **5**–**7** and their subsequent reactions to the corresponding bismuth monohydrides and monodeuterides **8**–**13**.

With bismuthinidene Ar*Bi **4** in hand, we became interested in its reactivity. Surprisingly, **4** turned out to be remarkably stable in C_6_D_6_ solution and did not react even at 110 °C with water, H_2_, CO, or cyclohexene, respectively, whereas the oxidation reaction of **4** with wet air cleanly gave Ar*Bi(OH)_2_ (**2**). In contrast, oxidative addition reactions of **4** with MeI, EtBr, and *i*‐PrBr gave the corresponding bismuthanes Ar*Bi(R)X (**5**, **6**, **7**; Scheme 3), whereas no reaction was observed with alkyl chlorides, most likely caused by the stronger C─Cl bond. Bismuthanes **5**–**7** are thermally stable in solution (110 °C, 1 h, C_6_D_6_) and can be stored at ambient temperature in isolated form for months. Mono‐coordinated triplet bismuthinidenes seem to be generally capable of the oxidative addition of C─X bonds of alkyl bromides or iodides, as the reaction of MeI with bismuthinidene **X** was also reported by Pang et al.^[^ ^19^ ^]^

**Figure 5:**
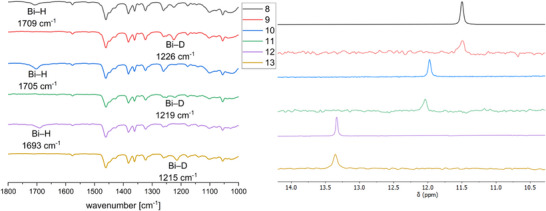
a) IR spectra of bismuth monohydrides and monodeuterides **8**–**13** (absorbance normalized and in arbitrary units, for unmanipulated spectra, see Supporting Information). b) Overlay of the ^1^H (**8**, **10**, **12**) and ^2^H NMR spectra (**9**, **11**, **13**) in the spectral range from 10 to 14 ppm, allowing for an assignment of the Bi─H absorption bands.

The reaction of bismuthanes **5**–**7** with LiAlH_4_ or LiAlD_4_ afforded the corresponding bismuth monohydrides or monodeuterides **8**–**13**, which were isolated as colorless solids. The IR spectra of the monohydrides **8**, **10**, and **12** show Bi─H absorption bands (**8**: 1709 cm^−1^, **10**: 1705 cm^−1^, and **12**: 1693 cm^−1^, Figure 5a), which shift to smaller wavenumbers in case of the corresponding monodeuterides (**9**: 1226 cm^−1^, **11**: 1219 cm^−1^, and **13**: 1215 cm^−1^). The Bi─D absorption bands were assigned in the IR spectra (Figure 5a). Both (Mes_2_Ph)_2_BiH (1759 cm^−1^) and [(Me_3_Si)_2_CH]_2_BiH (1690 cm^−1^) show comparable Bi─H absorption bands in the IR spectra.^[^ ^25^, ^41^ ^]^ In addition, the IR spectrum of monohydride **8** was simulated by means of B3LYP‐D3BJ (Figure S79), and the stretching frequency for the Bi─H bond is calculated to a value of 1718 cm^−1^, which agrees with the experimental result. All bismuth monohydrides show characteristic ^1^H NMR resonances for the Bi─H unit (**8**: 11.50 ppm; **10**: 11.97 ppm; and **12**: 13.33 ppm), which are in between those reported for (Mes_2_Ph)_2_BiH (19.64 ppm) and [(Me_3_Si)_2_CH]_2_BiH (3.24 ppm), respectively, and slightly shifted compared to that observed for the bismuth dihydride **3** (9.67 ppm). The ^1^H NMR spectra of the deuterium‐substituted bismuthanes **9**, **11**, and **13** are virtually identical, except for the missing Bi─H resonances, while ^2^H (D) NMR experiments of **9**, **11**, and **13** verified the assignment of the Bi─H bands in the ^1^H NMR spectra of **8**, **10**, and **12**, showing identical Bi─D resonances (Figure 5b).

Bismuthane **12** crystallizes in the space group *P*‐1 with four molecules in the elemental cell. The Bi─C_Ar_ bond length is between those observed for **1** and **2** (2.281(4) and 2.286(4) Å, two independent molecules). The isopropyl group is unfortunately too disordered to be well resolved and the hydrogen atom was not observed.

The formation of thermally stable bismuthanes **8**–**13** (110 °C, 1 h, C_6_D_6_) is remarkable, since bismuth hydrides are typically rather thermolabile compounds. For instance, BiH_3_, MeBiH_2_, and Me_2_BiH, which have been prepared at very low temperature (−110 °C) and characterized by vapor pressure measurements, IR (BiH_3_: 1734 cm^−1^) and MW spectroscopy, respectively, were found to disproportionate at higher temperatures to BiH_3_ and BiMe_3_. BiH_3_ then decomposes to bismuth metal and H_2_.^[^ ^42^ ^]^ To the best of our knowledge, the only structurally characterized stable bismuth monohydride is (Mes_2_Ph)_2_BiH,^[^ ^25^ ^]^ while [(Me_3_Si)_2_CH]_2_BiH has been only characterized by NMR and IR spectroscopy.^[^ ^41^ ^]^ Thermolysis of (Mes_2_Ph)_2_BiH and [(Me_3_Si)_2_CH]_2_BiH yielded the corresponding dibismuthenes [(Mes_2_Ph)Bi]_2_ and [(Me_3_Si)_2_CH]_2_Bi]_2_, respectively. Bismuthanes **8**–**13** are promising reagents for further transformation reactions due to the weakness of the Bi─H bond, and we are currently investigating the possibility of hydrogenation reactions in detail.

## Conclusion

The introduction of the sterically demanding septiphenyl ligand Ar* allowed the synthesis of the mono‐coordinated bismuthinidene Ar*Bi **4**. **4** is formed by dehydrogenation reaction of the corresponding bismuth dihydride Ar*BiH_2_ **3**, which was prepared in situ and characterized by ^1^H NMR spectroscopy at low temperature in solution. Kinetically stabilized bismuthinidene **4** adopts the triplet ground state according to quantum chemical calculations and was found to react with several alkyl halides with oxidative addition to halide‐substituted bismuthanes Ar*Bi(R)X, which are convenient starting reagents for the synthesis of room temperature‐stable bismuth monohydrides Ar*Bi(R)H and monodeuterides Ar*Bi(R)D.

## Supporting Information

The authors have cited additional references within the Supporting Information.^[^ ^40^, ^43^, ^44^, ^45^, ^46^, ^47^, ^48^, ^49^, ^50^, ^51^, ^52^, ^53^, ^54^, ^55^, ^56^, ^57^, ^58^ ^]^

## Conflict of Interests

The authors declare no conflict of interest.

## Supporting information



Supporting Information

## Data Availability

The data that support the findings of this study are available in the Supporting Information of this article.
