# Supplementary material for: Synthesis and Reactivity of a Mono‐Coordinated Triplet Bismuthinidene
Source: Angew Chem Int Ed Engl. 2025 Jul 11;64(36):e202508250. doi: 10.1002/anie.202508250 (PMC12402843; doi:10.1002/anie.202508250)
Supplement: Supplementary file 1 — Supporting Information [file ANIE-64-e202508250-s001.pdf]

Supporting Information

Synthesis and Reactivity of a Mono-Coordinated Triplet Bismuthinidene

Yannick Schulte,[a] Timo Freese,[a] Christoph Wölper,[a] Jan Schulte,[b] Gebhard Haberhauer,[b]* and Stephan Schulz[a;c]*

[a] Institute of Inorganic Chemistry, University of Duisburg-Essen, Universitätsstraße 5-7, D-45141 Essen

E-mail: stephan.schulz@uni-due.de; https://www.uni-due.de/ak_schulz/index_en.php

[b] Institute of Organic Chemistry, University of Duisburg-Essen, Universitätsstraße 5-7, D-45141 Essen

E-mail: gebhard.haberhauer@uni-due.de; https://www.uni-due.de/akhaberhauer/

[c] Institute of Inorganic Chemistry and Center for Nanointegration Duisburg-Essen (CENIDE), University of Duisburg-Essen, Carl-Benz-Straße 199, D-47057 Duisburg.

Content

S3−S9 I. Experimental Section

S13−S48 II. Spectroscopic Data

S13−S14 Figure S2-S4: ^{1}H, ^{13}C NMR and IR spectra of **Ar*-2**.
S14−S15 Figure S5-S7: ^{1}H, ^{13}C NMR and IR spectra of **Ar*-3**.
S16−S17 Figure S8-S10: ^{1}H, ^{13}C NMR and IR spectra of **Ar*-5**.
S17−S18 Figure S11-S13: ^{1}H, ^{13}C NMR and IR spectra of **Ar*-7**.
S19−S20 Figure S14-S16: ^{1}H, ^{13}C NMR and IR spectra of **Ar*I**.
S20−S21 Figure S17-S19: ^{1}H, ^{13}C NMR and IR spectra of **Ar*Li·Li*t*-Bu**.
S22−S23 Figure S20-S22: ^{1}H, ^{13}C NMR and IR spectra of **Ar*H**.
S23 Figure S23: ^{1}H NMR spectrum of impure **1**.
S24−S25 Figure S24-S26: ^{1}H, ^{13}C NMR and IR spectra of **2**.
S25 Figure S27: *In situ* ^{1}H NMR spectra of a mixture of **2** and $BH_3 \cdot SMe_2$ at different temperatures.
S26 Figure S28: *In situ* ^{1}H NMR spectrum of a mixture of **2** and $BH_3 \cdot SMe_2$ at 25 °C.
S26 Figure S29: *In situ* ^{1}H NMR spectrum of a mixture of **2** and $BH_3 \cdot SMe_2$ at 25 °C, low-field section.
S27−S30 Figure S30-S36: ^{1}H, ^{13}C NMR, UV-vis and IR spectra and a photograph of **4**.
S31−S32 Figure S37-S39: ^{1}H, ^{13}C NMR and IR spectra of **5**.
S32−S33 Figure S40-S42: ^{1}H, ^{13}C NMR and IR spectra of **6**.
S34−S35 Figure S43-S45: ^{1}H, ^{13}C NMR and IR spectra of **7**.
S35−S36 Figure S46-S48: ^{1}H, ^{13}C NMR and IR spectra of **8**.
S37−S38 Figure S49-S52: ^{1}H, ^{2}H, ^{13}C NMR and IR spectra of **9**.
S39−S40 Figure S53-S55: ^{1}H, ^{13}C NMR and IR spectra of **10**.
S41−S42 Figure S56-S59: ^{1}H, ^{2}H, ^{13}C NMR and IR spectra of **11**.
S42−S43 Figure S60-S62: ^{1}H, ^{13}C NMR and IR spectra of **12**.
S44−S45 Figure S63-S66: ^{1}H, ^{2}H, ^{13}C NMR and IR spectra of **13**.
S46 Figure S67: Comparison of IR spectra of **8** – **13**. Assignment of Bi–H and Bi–D stretching bands.
S47 Figure S68: Overlay of IR spectra of **8** and **9**.
S47 Figure S69: Overlay of IR spectra of **10** and **11**.
S48 Figure S70: Overlay of IR spectra of **12** and **13**.

S49−S55 III. Crystallographic Details

S52 Figure S71: Molecular structure of **Ar*-7** in the solid state
S52 Figure S72: Molecular structure of **Ar*I** in the solid state
S53 Figure S73: Molecular structure of **Ar*Li·Li*t*-Bu** in the solid state
S53 Figure S74: Molecular structure of **Ar*H** in the solid state
S54 Figure S75: Molecular structure of **1** in the solid state
S54 Figure S76: Molecular structure of **2** in the solid state
S55 Figure S77: Molecular structure of **4** in the solid state
S55 Figure S78: Molecular structure of **12** in the solid state

S56−S58 IV. Buried Volume Calculations

S59−S75 V. Computational Details

S59 Table S1. Energies for the transition from ground state (state 1) to the excited states 2–21.
S60 Figure S79: Overlay of measured (black) and calculated IR spectra of compound **8** simulated by means of B3LYP-D3BJ. The scale factor was determined to a value of 0.9486. The stretching frequency for the Bi–H bond is calculated to a value of 1718 cm^{-1}.
S61 Figure S80: Active orbitals of bismuthinidene **4**.
S62 Figure S81: Lowest states of **4** calculated by means of NEVPT2. The energies of the states are given in relation to state 1.

S76-S77 VI. References

I. Experimental Section

I) Synthesis of Ar*I

General Procedures. The starting materials **Ar*-1**, **Ar*-4**, **Ar*-6**, *n*-BuLi (2.5 M in *n*-hexane), Br_2, and Mg turnings were commercially available and used as received. MeI, EtBr, *i*-PrBr, *t*-BuBr, and *t*-BuCl were dried over CaH_2 for several days before use. I_2 was purified by sublimation. $AlBr_3$ was prepared from the elements and distilled twice from elemental Al. Commercially available *t*-BuLi in *n*-pentane was filtered, concentrated until incipient crystallization, redissolved by slight warming, and crystallized at -30 °C. Commercially available $LiAlH_4$ in Et_2O was filtered and freed from all volatiles *in vacuo*. THF, *n*-hexane, benzene, and toluene were dried by reflux over sodium-potassium alloy, followed by distillation.

Standard Schlenk and glove box techniques were used. Aqueous workup and subsequent handling of air-stable compounds **Ar*-1 – Ar*-7, Ar*I**, **Ar*H,** and **2** were performed in air without any special precautions.

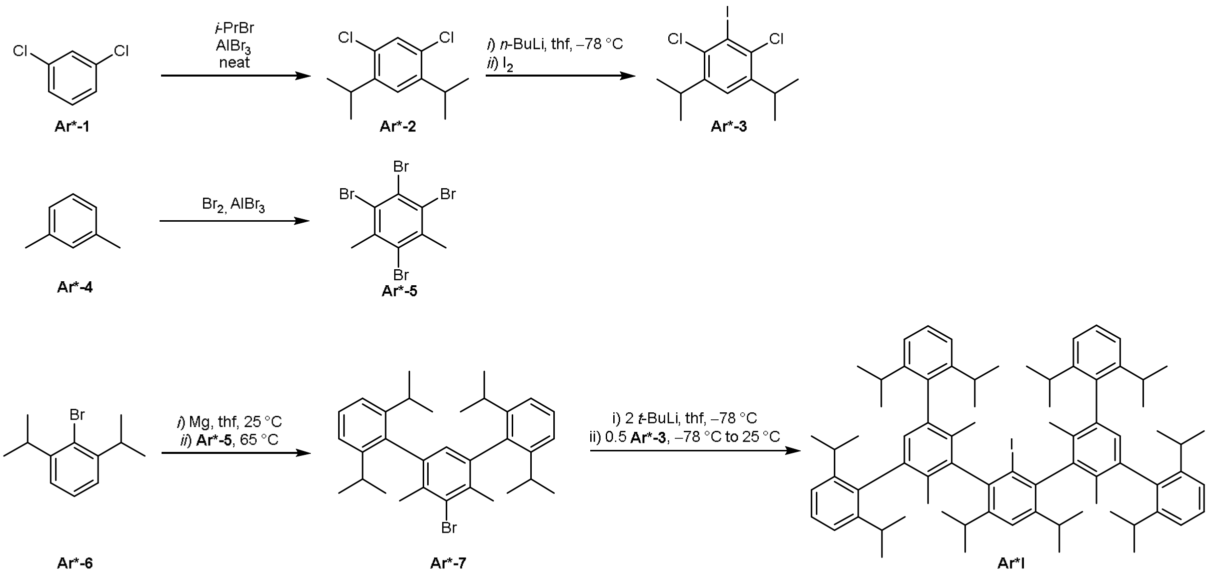

Figure S1: Synthesis of Ar*I

Synthesis of Ar*-2. 3.7 mmol (1 g) of $AlBr_3$ were dissolved in 349 mmol (51.3 g) of 1,3-dichlorobenzene (**Ar*-1**) and 747 mmol (91.9 g) *i*-PrBr were added dropwise within 20 min at 25 °C. The resulting orange solution was stirred for 30 min and then poured on 100 g of ice. The mixture was stirred at room temperature until thawed and diluted with 400 mL 1 M hydrochloric acid. The aqueous phase was discarded and the organic phase was dried over K_2CO_3. All volatiles were removed at 60 °C/10 mbar, yielding a colorless liquid.

Yield: 74.5 g (322 mmol, 92 %). Melting point: < 25 °C

^{1}H NMR (400 MHz, C_6D_6) δ = 7.36 (s, 1H, Ar*H*), 7.08 (s, 1H, Ar*H*), 3.29 (sept, 2H, $^3J_{HH}$=6.9 Hz, ArC*H*(CH_3)$_2$), 1.07 (d, 12H, $^3J_{HH}$=6.9 Hz, ArCH(C*H*$_3$)$_2$) ppm.

^{13}C NMR (101 MHz, C_6D_6) δ = 144.7 (Ar), 131.5 (Ar), 130.1 (Ar), 124.4 (Ar), 30.3 Ar*C*H(CH_3)$_2$, 22.6 ArCH(*C*H$_3$)$_2$ ppm.

ATR-IR: ν = 2964, 2928, 2871, 1594, 1549, 1470, 1445, 1364, 1391, 1343, 1251, 1226, 1131, 1097, 1066, 935, 890, 865, 849, 815, 698, 572, 513, 477 cm^{-1}.

Elemental analysis calcd. for $C_{12}H_{16}Br_2$: C 45.0, H 5.04, found: C 45.4, H 5.13.

Comment: Corrosive HBr gas is evolved during the reaction. The exclusion of O_2 is not necessary.

Synthesis of Ar*-3. 3 mmol (693 mg) of **Ar*-2** were dissolved in 30 mL THF at 25 °C and then cooled to -78 °C. 4 mmol of *n*-BuLi (1.6 mL of a 2.5 M solution in hexanes) were diluted with 10 mL of *n*-hexane and added dropwise to the solution of **Ar*-2** in the course of 20 min. The combined solutions were stirred for 20 min. 5 mmol of I_2 (1.27 g), dissolved in 10 mL THF were added dropwise within 20 min. In the beginning, the fast decolorization of every drop of I_2 solution was observed. The decolorization became slower and at the end of the addition the color of iodine persisted. The formation of a colorless precipitate was observed. After the addition, the solution was warmed to 25 °C, during which the precipitate redissolved. 10 mmol of Na_2SO_3 (1.26 g), 50 mL of water, and 50 mL of *n*-hexane were added and the biphasic solution was stirred until colorless. After separation, the organic phase was washed with 50 mL of aqueous HCl (37 %) and dried with $MgSO_4$. **Ar*-3** was obtained after removal of all volatiles at 60 °C/10 mbar as colorless liquid.

Yield: 927 mg (2,60 mmol, 87 %). Melting point: < 25 °C

^{1}H NMR (400 MHz, C_6D_6) δ = 7.00 (s, 1H, Ar*H*), 3.26 (sept, 2H, $^3J_{HH}$=6.9 Hz, ArC*H*(CH_3)$_2$), 0.98 (d, 12H, $^3J_{HH}$=6.9 Hz, ArCH(C*H*$_3$)$_2$) ppm.

^{13}C NMR (101 MHz, C_6D_6) δ = 145.6 (Ar), 136.8 (Ar), 124.0 (Ar), 107.3 (Ar), 32.7 (Ar*C*H(CH_3)$_2$), 22.6 (ArCH(*C*H$_3$)$_2$) ppm.

ATR-IR: ν = 2962, 2928, 2869, 1531, 1459, 1409, 1386, 1362, 1330, 1219, 1161, 1106, 1059, 1000, 946, 924, 885, 849, 813, 757, 732, 700, 585, 542, 531, 411 cm^{-1}.

Elemental analysis calcd. for $C_{12}H_{16}Br_2I$: C 32.3, H 3.39, found: C 32.6, H 3.41.

Synthesis of Ar*-5. 10 mmol (2.67 g) of $AlBr_3$ were suspended in 200 mmol (21.2 g) of 1,3-dimethylbenzene. The suspension was cooled to 0 °C and 1000 mmol (160 g) Br_2 were added dropwise in the course of 3 h. The suspension was warmed to 25 °C within 12 h. All volatiles were removed *in vacuo* (water aspirator, cold trap cooled with dry ice/*i*-PrOH), and the resulting solid was heated to reflux with 500 mL 1M aqueous HCl for 30 min. The suspension was cooled to room temperature and the product was isolated by filtration and washed with 500 mL methanol in several portions. **Ar*-5** was then freed from all volatiles *in vacuo*, dissolved in 600 mL boiling toluene, filtered while hot and crystallized at 25 °C.

Yield: 64.6 g (153 mmol, 77 %). Melting point: 248 – 254 °C (Lit: 246 – 248 °C)[1]

^{1}H NMR (400 MHz, C_6D_6) δ = 2.35 (s, ArCH_3) ppm.

^{13}C NMR (101 MHz, C_6D_6) δ = 139.0 (Ar), 128.5 (Ar), 126.7 (Ar), 125.8 (Ar), 27.5 (ArCH$_3$) ppm.

ATR-IR: ν = 2919, 1535, 1510, 1431, 1371, 1334, 1264, 1228, 1059, 994, 964, 894, 829, 660, 628, 454 cm^{-1}.

Elemental analysis calcd. for $C_8H_6Br_4$: C 22.8, H 1.43, found: C 22.8, H 1.45.

Comment: Corrosive HBr gas is evolved during the reaction.

Synthesis of Ar*-7. 100 mmol (24.3 g) of magnesium turnings were rapidly stirred for 24 h under argon to activate the metal surface.[2] 200 mL of THF were added and the suspension was cooled to 0 °C. 100 mmol (24.1 g) of **Ar*-6** were added and the suspension was warmed to 25 °C within of 24 h. 33 mmol (13.9 g) **5** were then added to the resulting black suspension, which still contained a small amount of unreacted magnesium turnings. The resulting suspension was heated to 65 °C and stirred for 24 h. After cooling to 0 °C, 20 mL of water were added dropwise, and the suspension was then diluted with 250 mL 37 % aqueous HCl and 500 mL DCM. The phases were separated, and the organic phase was dried with $MgSO_4$. It was then diluted with an equal volume of MeOH and concentrated at the rotary evaporator until a substantial amount of colorless crystals separated. The suspension was then cooled to room temperature. The product was isolated by filtration in a Büchner funnel, washed with methanol and freed of adhering solvents by passing a stream of air through the Büchner funnel for a few hours.

Yield: 11.3 g (22.4 mmol; 68 %). Melting point: 230 – 231 °C

^{1}H NMR (400 MHz, C_6D_6) δ = 7.29 (t, 2H, $^{3}J_{HH}$=7.7 Hz, iPr$_2$ArH), 7.15 (overlapping with C_6D_5H signal), 6.78 (s, 1H,Me$_2$ArH), 2.71 (sept, 4H, $^{3}J_{HH}$=6.8 Hz, ArCH(CH$_3$)$_2$), 2.31 (s, 6H, ArCH_3), 1.07 (d, 24H, $^{3}J_{HH}$=6.8 Hz, ArCH(CH_3)$_2$) ppm.

^{13}C NMR (101 MHz, C_6D_6) δ = 146.8 (Ar), 139.1 (Ar), 138.3 (Ar), 136.1 (Ar), 130.8 (Ar), 130.0 (Ar), 128.6 (Ar), 123.2 (Ar), 30.9 (ArCH(CH$_3$)$_2$), 24.9 (ArCH(CH$_3$)$_2$), 24.0 (ArCH(CH$_3$)$_2$), 22.1 (ArCH$_3$) ppm.

ATR-IR: ν = 3066, 2959, 2921, 2865, 1594, 1578, 1531, 1461, 1443, 1386, 1362, 1328, 1307, 1249, 1197, 1174, 1111, 1102, 1052, 1014, 989, 942, 901, 809, 784, 757, 725, 673, 646, 612, 581, 556, 502, 450, 441 cm^{-1}.

Elemental analysis calcd. for $C_{32}H_{41}Br$: C 76.0, H 8.17, found: C 75.6, H 8.27.

Synthesis of Ar*I. 1 mmol (506 mg) of **Ar*-7** was dissolved in 10 mL THF at 25 °C. The solution was cooled to -78 °C and 2 mmol (128 mg) *t*-BuLi in 0.8 mL *n*-hexane were added dropwise. The solution was stirred for 30 min at -78 °C. 0.5 mmol (179 mg) of **Ar*-3**, dissolved in 0.8 ml THF, were added and the solution was slowly warmed to 25 °C over night. The light beige suspension was diluted with 20 mL aqueous 37 % HCl and 20 mL DCM. The (upper) aqueous phase was discarded and the (lower) organic phase was dried with $MgSO_4$. All volatiles were removed at the rotary evaporator. The residue was dissolved in 10 mL of hot toluene and the product was crystallized at -30 °C. The crystals contain 1 eq. of toluene.

An analytical sample for NMR analysis was further re-crystallized from C_6D_6.

Yield: 185 mg (0.150 mmol; 30 %). Melting point: 325 °C (turns brown without melting)

^{1}H NMR (400 MHz, C_6D_6) δ = 7.50 (s, 1H, ArH, *para* to I), 7.33 (t, 4H, $^{3}J_{HH}$=7.7 Hz, iPr$_2$ArH), 7.24 – 7.19 (m, 8H, iPr$_2$ArH), 6.94 (s, 2H, Me$_2$ArH) 3.14 (sept, 4H, $^{3}J_{HH}$=6.8 Hz, ArCH(CH$_3$)$_2$) 3.00 – 2.87 (m, 6H, ArCH(CH$_3$)$_2$), 1.88 (s, 12H, ArCH_3), 1.27 (d, 12H, $^{3}J_{HH}$=6.8 Hz, ArCH(CH_3)$_2$) 1.23 – 1.14 (m, 48H, ArCH(CH_3)$_2$) ppm.

^{13}C NMR (101 MHz, C_6D_6) δ = 147.2 (Ar), 147.1 (Ar), 146.5 (Ar), 145.3 (Ar), 143.3 (Ar), 138.9 (Ar), 138.5 (Ar), 133.5 (Ar), 130.9 (Ar), 123.5 (Ar), 123.2 (Ar), 123.0 (Ar), 32.7 (Ar*C*H(CH_3)$_2$), 31.0 (Ar*C*H(CH_3)$_2$), 30.8 (Ar*C*H(CH_3)$_2$), 24.8 (ArCH($\mathit{C}H_3$)$_2$), 24.2 (ArCH($\mathit{C}H_3$)$_2$), 24.0 (ArCH($\mathit{C}H_3$)$_2$), 24.0 (ArCH($\mathit{C}H_3$)$_2$), 18.5 (Ar$\mathit{C}H_3$) ppm.

ATR-IR: ν = 3059, 3023, 2957, 2923, 2865, 1578, 1526, 1495, 1461, 1441, 1380, 1362, 1325, 1253, 1179, 1104, 1055, 991, 899, 813, 775, 754, 723, 689, 623, 576, 497, 468, 450 cm^{-1}.

Elemental analysis calcd. for $C_{76}H_{97}I$: C 80.3, H 8.60, found: C 80.1, H 8.37.

Synthesis of Ar*Li·Li*t*-Bu. 1 mmol (1.23 g) of **Ar*I** was suspended in 50 mL of benzene. 10 mmol (5.88 mL of a 1.7M solution in *n*-pentane) of *t*-BuLi were added and the suspension was stirred for 1 h at 25 °C. The suspension was freeze-dried. The residue was extracted at 25 °C with 60 mL of toluene in two portions. The extracts were filtered through a syringe filter (PTFE, 0.22 µm pore size) to remove residual suspended solid (probably finely divided LiI). The extract was concentrated *in vacuo* until incipient crystallization (approx. 50 mL). The solids were re-dissolved by slight warming and the product was crystallized at -30 °C. The crystals contain 2 eq. of toluene and varying but small amounts of **Ar*H**.

Yield: 950 mg (0.950 mmol; 75 %). Melting point: approx. 210 °C (turns brown without melting)

^{1}H NMR (400 MHz, C_6D_6) δ = 7.40 (s, 1H, Ar*H*, *para* to Li), 7.30 (t, 4H, $^{3}J_{HH}$=7.7 Hz, iPr$_2$Ar*H*), 7.20 – 7.17 (m, 8H, iPr$_2$Ar*H*), 7.01 (s, 2H, Me$_2$Ar*H*), 2.91 (sept, 4H, $^{3}J_{HH}$=6.8 Hz, ArC*H*(CH_3)$_2$) , 2.78 (sept, 4H, $^{3}J_{HH}$=6.8 Hz, ArC*H*(CH_3)$_2$) , 2.63 (sept, 2H, $^{3}J_{HH}$=6.8 Hz, ArC*H*(CH_3)$_2$), 2.05 (s, 12H, ArCH_3), 1.30 (d, 12H, $^{3}J_{HH}$=6.8 Hz, ArCH(CH_3)$_2$) , 1.26 (d, 12H, $^{3}J_{HH}$=6.8 Hz, ArCH(CH_3)$_2$), 1.20 (d, 12H, $^{3}J_{HH}$=6.8 Hz, ArCH(CH_3)$_2$), 1.16 (d, 12H, $^{3}J_{HH}$=6.8 Hz, ArCH(CH_3)$_2$), 1.11 (d, 12H, $^{3}J_{HH}$=6.8 Hz, ArCH(CH_3)$_2$), 1.04 (s, 9H, Li$_2$C(CH_3)$_3$) ppm.

^{13}C NMR (101 MHz, C_6D_6) δ = 147.9 (Ar), 147.0 (Ar), 146.3 (Ar), 144.6 (Ar), 141. 9 (Ar), 140.2 (Ar), 139.8 (Ar), 139.3 (Ar), 138.1 (Ar), 137.3 (Ar), 135.6 (Ar), 132.0 (Ar), 123.4 (Ar), 123.4 (Ar), 33.4 (Li$_2$C($\mathit{C}H_3$)$_3$), 31.6 (Ar*C*H(CH_3)$_2$), 30.8 (Ar*C*H(CH_3)$_2$), 25.4 (ArCH($\mathit{C}H_3$)$_2$), 25.4 (ArCH($\mathit{C}H_3$)$_2$), 25.0 (ArCH($\mathit{C}H_3$)$_2$), 24.3 (ArCH($\mathit{C}H_3$)$_2$), 24.2 (ArCH($\mathit{C}H_3$)$_2$), 19.3 (Ar$\mathit{C}H_3$) ppm. We could not identify the ^{13}C signals of the Li$_2$-*C* carbon atoms, possibly due to broadening by coupling with the ^{6}Li and ^{7}Li nuclei.

ATR-IR: ν = 3029, 2957, 2923, 2862, 2783, 2691, 1580, 1463, 1382, 1362, 1314, 1055, 989, 903, 813, 775, 752, 727, 569, 461 cm^{-1}.

Comment: **Ar*H** is probably formed via the reaction of **Ar*Li·Li*t*-Bu** with isobutylene. *In situ* ^{1}H NMR showed a decrease of the concentration of **Ar*Li·Li*t*-Bu** and isobutylene and an increase of the concentration of **Ar*H** in the reaction solution over time. It is therefore not advisable to use longer reaction times.

When the reaction is performed in toluene instead of benzene, the obtained product is yellow, probably due to contamination with benzyllithium. The use of toluene for work-up and crystallization is less problematic.

Synthesis of Ar*H. 10 µmol (12.7 mg) of **Ar*Li·Li*t*-Bu** were suspended in 1 mL of degassed water and stirred for 30 min at 25 °C. All volatiles were removed *in vacuo*. The residue was dissolved in 1 mL DCM, filtered, diluted with 1 mL of methanol, concentrated on the rotary evaporator until incipient crystallization, redissolved by slight warming, and the product was crystallized at -30 °C.

Yield: 7.8 mg (7.7 µmol, 77 %). Melting point: 315 – 330 °C (melting under decomposition)

^{1}H NMR (400 MHz, C_6D_6) δ = 7.60 (s, 1H, terphenyl$_2$Ar*H*), 7.32 (t, 4H, $^{3}J_{HH}$=7.7 Hz, iPr$_2$Ar*H*), 7.29 (s, 1H, terphenyl$_2$Ar*H*), 7.20 (t, 8H, $^{3}J_{HH}$=7.7 Hz, iPr$_2$Ar*H*), 6.91 (s, 2H, Me$_2$Ar*H*), 2.96 –

2.82 (m, 10H, ArCH(CH_3)$_2$), 1.96 (s, 12H, ArCH_3), 1.26 (d, 12H, $^3J_{HH}$=6.8 Hz, ArCH(CH_3)$_2$), 1.17 – 1.12 (m, 24H, ArCH(CH_3)$_2$), 1.06 (d, 12H, $^3J_{HH}$=6.8 Hz, ArCH(CH_3)$_2$) ppm.

^{13}C NMR (101 MHz, C_6D_6) δ = 146.8 (Ar), 146.8 (Ar), 145.8 (Ar), 141.8 (Ar), 139.0 (Ar), 138.2 (Ar), 138.1 (Ar), 134.0 (Ar), 130.5 (Ar), 130.0 (Ar), 123.2 (Ar), 123.1 (Ar), 132.1 (Ar), 31.0 (Ar*C*H(CH_3)$_2$), 30.8 (Ar*C*H(CH_3)$_2$), 30.5 (Ar*C*H(CH_3)$_2$), 25.1 (ArCH(CH_3)$_2$), 24.1 (ArCH(CH_3)$_2$), 23.8 (ArCH(CH_3)$_2$), 19.1 (ArCH_3) ppm.

ATR-IR: ν = 3061, 2955, 2923, 2865, 1578, 1463, 1384, 1362, 1251, 1055, 991, 897, 813, 750, 567, 441 cm^{-1}.

Elemental analysis calcd. for $C_{76}H_{98}$: C 90.2, H 9.76, found: C 90.0, H 9.71.

Synthesis of 1. 16 µmol (20 mg) of **Ar*Li·Li*t*-Bu** and 59 µmol (20 mg) Bi(NMe_2)$_3$ were dissolved in 0.5 mL C_6D_6 at 25 °C and a ^{1}H NMR spectrum was recorded, showing the clean formation of a single new ligand species. The suspension was freeze-dried and re-dissolved in 0.5 mL C_6D_6. A second ^{1}H NMR spectrum was recorded, showing that this procedure removed most, but not all Bi(NMe_2)$_3$. The solution was concentrated to approx. 100 µL and stored at 6 °C for 24 h, yielding a small amount of yellow crystals suitable for sc-XRD.

^{1}H NMR (400 MHz, C_6D_6) δ = 7.57 (s, 1H, Ar*H*, *para* to Bi), 7.34 (t, 4H, $^3J_{HH}$=7.7 Hz, iPr$_2$Ar*H*), 7.27 – 7.19 (m, 8H, iPr$_2$Ar*H*), 6.98 (s, 2H, Me$_2$Ar*H*), 3.39 (s, Bi(N(CH_3)$_2$)$_2$), 3.14 (sept, 4 H, $^3J_{HH}$=6.8 Hz, ArCH(CH_3)$_2$), 2.85 (sept, 4H, $^3J_{HH}$=6.8 Hz, ArCH(CH_3)$_2$), 2.61 (sept, 2 H, $^3J_{HH}$=6.8 Hz, ArCH(CH_3)$_2$) 2.01 (s, 12H, ArCH_3), 1.31 (d, 12H, $^3J_{HH}$=6.8 Hz, ArCH(CH_3)$_2$) 1.24 – 1.17 (m, 36 H, ArCH(CH_3)$_2$) 1.12 (d, 12H, $^3J_{HH}$=6.8 Hz, ArCH(CH_3)$_2$) ppm.

Comment: **1** decomposes both in solution and in the solid state when stored at 25 °C. No further analysis besides ^{1}H NMR and sc-XRD was performed. In the following procedures it was prepared *in situ* and used immediately.

Synthesis of 2. 474 µmol (600 mg) **Ar*Li·Li*t*-Bu** and 1.76 mmol (600 mg) Bi(NMe_2)$_3$ were dissolved in 10 mL toluene and stirred for 15 min at 25 °C. 33.3 mmol (600 mg) of H_2O were added and the mixture was stirred for 5 min at 25 °C. All volatiles were removed *in vacuo*. The residue was extracted with 100 mL of DCM and 100 mL of *n*-heptane. The combined extracts were concentrated on a rotary evaporator until incipient crystallization. 100 µL of water were added. The crystals were redissolved by warming and the product was crystallized at -20 °C, yielding colorless needles.

Yield: 530 mg (423 mmol, 89 %). Melting point: 318 – 322 °C (turns black while melting)

^{1}H NMR (400 MHz, C_6D_6) δ = 7.61 (s, 1H, Ar*H*, *para* to Bi), 7.32 (t, 4H, $^3J_{HH}$=7.7 Hz, iPr$_2$Ar*H*), 7.23 – 7.17 (m, 8H, iPr$_2$Ar*H*), 6.99 (s, 2H, Me$_2$Ar*H*), 3.04 (sept, 4 H, $^3J_{HH}$=6.8 Hz, ArCH(CH_3)$_2$), 2.93 – 2.69 (m, 8H, ArCH(CH_3)$_2$), 2.25 (s, 2H, Bi(O*H*)$_2$), 2.04 (s, 12H, ArCH_3), 1.27 (d, 12H, $^3J_{HH}$=6.8 Hz, ArCH(CH_3)$_2$), 1.22 (d, 12H, $^3J_{HH}$=6.8 Hz, ArCH(CH_3)$_2$) 1.19 (d, 12H, $^3J_{HH}$=6.8 Hz, ArCH(CH_3)$_2$) 1.17 – 1.12 (m, 24 H, ArCH(CH_3)$_2$) ppm.

^{13}C NMR (101 MHz, C_6D_6) δ = 149.5 (Ar), 147.2 (Ar), 146.5 (Ar), 144.7 (Ar), 139.5 (Ar), 139.1 (Ar), 138.2 (Ar), 135.6 (Ar), 132.1 (Ar), 124.3 (Ar), 123.4 (Ar), 123.1 (Ar), 30.9 (Ar*C*H(CH_3)$_2$), 30.9 (Ar*C*H(CH_3)$_2$), 30.6 (Ar*C*H(CH_3)$_2$), 25.1 (ArCH(CH_3)$_2$), 25.0 (ArCH(CH_3)$_2$), 24.4 (ArCH(CH_3)$_2$), 24.3 (ArCH(CH_3)$_2$), 24.0 (ArCH(CH_3)$_2$), 10.7 (ArCH_3) ppm.

ATR-IR: ν = 3610, 3560, 2957, 2923, 2867, 1576, 1459, 1386, 1359, 1343, 1323, 1231, 1052, 996, 967, 899, 813, 775, 752, 567, 531, 506, 445 cm^{-1}.

Elemental analysis calcd. for $C_{76}H_{99}BiO_2$: C 90.2, H 7.96, found: C 89.9, H 7.85.

Comment: When **2** is re-crystallized in the absence of water, a second, more soluble product with broad ^{1}H NMR signals is also formed. This can be re-converted to **12** by the addition of water. This is possibly an oxygen-bridged dimer.

Synthesis of 3. 1.6 µmol (2 mg) **2** were dissolved in 0.5 mL toluene-d_8 and a ^{1}H NMR spectrum was recorded. 10.7 µmol (0.8 mg, 1 µL) $BH_3 \cdot SMe_2$ was added at −78 °C. The sample was transferred into an NMR spectrometer and ^{1}H NMR spectra of the yellow solution were recorded at −60 °C, −40 °C, −20 °C, 0 °C, 20 °C, and 40 °C (Figure S27), respectively, with 5 min waiting time between different temperatures.

At −60 °C, no meaningful results were obtained, probably due to the low solubility of the sample at this temperature, and due to the high viscosity of the solution, resulting in line broadening. At −40 °C, the nearly quantitative formation of **3** was observed. **3** exhibits a characteristic Bi-H signal at 9.64 ppm with an integral of 2. With increasing temperature, **3** was gradually converted to **4**, accompanied by the evolution of dihydrogen. At 40 °C, this reaction was nearly complete (Figure S27).

In a preliminary experiment, 1.6 µmol (2 mg) **2** and a drop of $BH_3 \cdot SMe_2$ were dissolved in 0.5 mL C_6D_6 and a ^{1}H NMR spectrum was measured at 25 °C after approx. 5 min (Figure S28), also showing the formation of **3** and **4**.

3 was only characterized by in situ ^{1}H NMR spectroscopy due to its limited stability.

Synthesis of 4. A mixture of 207 µmol (260 mg) of **2**, 527 µmol (40 mg, 50 µL) of $BH_3 \cdot SMe_2$ and 3 mL of toluene was stirred for 24 h at 25 °C, forming an almost colorless to very light pink suspension containing a small amount of suspended metal particles. All volatiles were removed *in vacuo*. The residue was extracted successively with 10 mL and 2 mL of toluene at 110 °C. The filtrate was stored at -30 °C for 24 h, resulting in the formation of faint pink needle-shaped crystals. These were re-crystallized from 5 mL of benzene at 25 °C.

Yield: 183 mg (150 µmol, 72 %). Melting point: 227 °C (partial melting under decomposition; turns yellow)

^{1}H NMR (400 MHz, C_6D_6) δ = 7.63 (t, 4H, $^{3}J_{HH}$=7.7 Hz iPr$_2$Ar*H*), 7.51 – 7.46 (m, 4H, iPr$_2$Ar*H*), 7.43 – 7.38 (m, 4H, iPr$_2$Ar*H*), 6.84 (s, 2H, Me$_2$Ar*H*), 4.82 (sept, 4H, $^{3}J_{HH}$=6.9 Hz, ArC*H*(CH$_3$)$_2$), 3.54 (s, 12H, ArC*H*$_3$), 3.22 (sept, 4H, $^{3}J_{HH}$=6.8 Hz, ArC*H*(CH$_3$)$_2$), 2.55 (d, 12H, $^{3}J_{HH}$=6.9 Hz, ArCH(C*H*$_3$)$_2$), 1.36 (d, 12H, $^{3}J_{HH}$=6.8 Hz, ArCH(C*H*$_3$)$_2$), 1.28 (d, 12H, $^{3}J_{HH}$=6.8 Hz, ArCH(C*H*$_3$)$_2$), 1.17 (d, 12H, $^{3}J_{HH}$=6.8 Hz, ArCH(C*H*$_3$)$_2$), 0.65 (d, 12H, $^{3}J_{HH}$=6.8 Hz, ArCH(C*H*$_3$)$_2$), -1.41 (s, 1H, Ar*H*, *para* to Bi) ppm. A septet (ArC*H*(CH$_3$)$_2$) is overlapped by the doublet at 1.36 ppm.

^{13}C NMR (101 MHz, C_6D_6) δ = 231.6 (Bi-C-*C*), 169.7 (Ar), 158.9(Ar), 148.7(Ar), 148.0(Ar), 147.1(Ar), 141.0(Ar), 139.0(Ar), 133.7(Ar), 129.2(Ar), 126.3(Ar), 123.5(Ar), 91.2(Ar), 55.6, 42.3, 37.5, 34.0, 31.6, 25.5, 25.1, 24.2, 19.0, -194.7 (broad, Bi-*C*) ppm.

ATR-IR: ν = 3061, 3034, 2957, 2923, 2865, 1580, 1461, 1425, 1380, 1362, 1321, 1251, 1174, 1102, 1052, 1039, 994, 939, 899, 881, 813, 799, 777, 754, 673, 621, 567, 533, 508, 447 cm^{-1}.

Elemental analysis calcd. for $C_{76}H_{97}Bi$: C 74.9, H 8.02, found: C 75.1, H 8.12.

General Synthesis of 5 – 7. 20 µmol (24.4 mg) of **4** and 200 µmol of MeI (28.4 mg, 12.4 µL), EtBr (21.8 mg, 14.9 µL), or *i*-PrBr (24.6 mg, 18.8 µL) were suspended/dissolved in 0.5 mL C_6D_6 and heated to 110 °C until all solids dissolved and *in situ* NMR spectroscopy indicated complete conversion (15 min (MeI), 30 min (EtBr), 1 h (*i*-PrBr)). The solutions were then freeze-dried, yielding **5** − **7** in sufficient purity. Compound **5** is an orange solid, while compounds **6** and **7** are yellow solids.

Compounds 5 − 7 can be crystallized from *n*-hexane at -30°C. Unfortunately, this process *reduces* their purity because the solubility of **Ar*H**, which is the main impurity, is lower than that of **5 − 7** and therefore it is enriched in the crystals. In addition, **Ar*H** and compounds **5 − 7** tend to co-crystallize.

Comment. No reaction was observed in analogous reactions with *i*-PrCl and *t*-BuCl (1 h 110 °C). After a prolonged reaction time (24 h), compound **4** and *t*-BuCl quantitatively reacted to **Ar*H**. The reaction of **4** and *t*-BuBr at 110 °C (10 min) resulted in complete, but unselective conversion to at least 3 different products and bismuth metal. At 25 °C, complete conversion to mainly one product was observed after 65 min. This product could not be isolated, because it decomposed when standing over night at 6 °C.

Analytical Data of 5. Yield: 8.7 mg (6.4 µmol, 91 %). Melting point: gradually turning colorless above 150 °C

^{1}H NMR (400 MHz, C_6D_6) δ = 7.59 (s, 1H, Ar*H*, *para* to Bi), 7.33 (t, 4H, $^{3}J_{HH}$=7.7 Hz, iPr$_2$Ar*H*), 7.25 – 7.18 (m, 8H, iPr$_2$Ar*H*), 6.99 (s, 2H, Me$_2$Ar*H*), 3.23 – 3.11 (m, 4H, ArC*H*(CH$_3$)$_2$), 2.85 – 2.67 (m, 6H, ArC*H*(CH$_3$)$_2$), 2.02 (s, 6H, ArC*H*$_3$), 2.01 (s, 6H, ArC*H*$_3$), 1.74 (s, 3H, BiCH$_3$) 1.36 (d, 6H, $^{3}J_{HH}$=6.8 Hz, ArCH(C*H*$_3$)$_2$), 1.27 (d, 6H, $^{3}J_{HH}$=6.8 Hz, ArCH(C*H*$_3$)$_2$), 1.22 – 1.10 (m, 48H, overlapped by byproducts, ArCH(C*H*$_3$)$_2$) ppm.

^{13}C NMR (101 MHz, C_6D_6) δ = 146.8 (Ar), 146.8 (Ar), 146.7 (Ar), 142.3 (Ar), 138.9 (Ar), 138.5 (Ar), 138.4 (Ar), 135.3 (Ar), 135.2 (Ar), 132.0 (Ar), 125.2 (Ar), 123.6 (Ar), 123.4 (Ar), 123.2 (Ar), 123.1 (Ar), 31.0 (Ar*C*H(CH$_3$)$_2$), 30.8 (Ar*C*H(CH$_3$)$_2$), 30.8 (Ar*C*H(CH$_3$)$_2$), 30.7 (Ar*C*H(CH$_3$)$_2$), 25.2 (ArCH(*C*H$_3$)$_2$), 24.7 (ArCH(*C*H$_3$)$_2$), 24.2 (ArCH(*C*H$_3$)$_2$), 24.1 (ArCH(*C*H$_3$)$_2$), 20.2 (Ar*C*H$_3$) ppm.

ATR-IR: ν = 3057, 2957, 2923, 2865, 1576, 1461, 1382, 1359, 1323, 1260, 1102, 1055, 901, 802, 754, 626, 569, 443 cm^{-1}.

Elemental analysis calcd. for $C_{77}H_{100}BiI$: C 68.9, H 7.80, found: C 69.5, H 7.97.

Analytical Data of 6. Yield: 8.0 mg (6.0 µmol, 85 %). Melting point: gradually turning brown above 262 °C

^{1}H NMR (400 MHz, C_6D_6) δ = 7.61 (s, 1H, Ar*H*, *para* to Bi), 7.32 (t, 4H, $^{3}J_{HH}$=7.7 Hz, iPr$_2$Ar*H*), 7.24 – 7.18 (m, 8H, iPr$_2$Ar*H*), 6.99 (s, 2H, Me$_2$Ar*H*), 3.37 – 3.27 (m, 1H, BiC*H*$_2$CH$_3$), 3.25 – 3.08 (m, 4H, ArC*H*(CH$_3$)$_2$), 2.86- 2.69 (m, 9H, ArC*H*(CH$_3$)$_2$, BiCH$_2$C*H*$_3$) 2.01 (s, 12H, ArC*H*$_3$), 1.47 (dq, 1H, $^{2}J_{HH}$=11.4 Hz, $^{3}J_{HH}$=7.9 Hz, BiC*H*$_2$CH$_3$), 1.34 (d, 6H, $^{3}J_{HH}$=6.8 Hz, ArCH(C*H*$_3$)$_2$), 1.30 (d, 6H, $^{3}J_{HH}$=6.8 Hz, ArCH(C*H*$_3$)$_2$), 1.24 – 1.16 (m, 36H, ArCH(C*H*$_3$)$_2$), 1.14 – 1.10 (m, 12H, ArCH(C*H*$_3$)$_2$) ppm.

^{13}C NMR (101 MHz, C_6D_6) δ = 149.0 (Ar), 147.1 (Ar), 146.9 (Ar), 146.8 (Ar), 146.8 (Ar), 138.9 (Ar), 138.5 (Ar), 138.4 (Ar), 135.2 (Ar), 135.2 (Ar), 131.9 (Ar), 125.1 (Ar), 123.6 (Ar), 123.4 (Ar), 123.2 (Ar), 123.1 (Ar), 48.9 (Bi*C*H$_2$CH$_3$), 31.1 (Ar*C*H(CH$_3$)$_2$), 31.0 (Ar*C*H(CH$_3$)$_2$), 30.9 (Ar*C*H(CH$_3$)$_2$), 30.8 (Ar*C*H(CH$_3$)$_2$), 30.7 (Ar*C*H(CH$_3$)$_2$), 25.5 (ArCH(*C*H$_3$)$_2$), 25.4 (ArCH(*C*H$_3$)$_2$), 25.4 (ArCH(*C*H$_3$)$_2$), 24.7 (ArCH(*C*H$_3$)$_2$), 24.7 (ArCH(*C*H$_3$)$_2$), 24.2 (ArCH(*C*H$_3$)$_2$), 19.8 (Ar*C*H$_3$)), 19.6 (Ar*C*H$_3$)) 11.0 (BiCH$_2$*C*H$_3$) ppm.

ATR-IR: ν = 3070, 2957, 2923, 2865, 1576, 1461, 1427, 1382, 1362, 1323, 1249, 1179, 1102, 1057, 994, 944, 901, 815, 804, 777, 754, 675, 621, 567, 495, 468, 445 cm^{-1}.

Elemental analysis calcd. for $C_{78}H_{102}BiBr$: C 69.5, H 7.97, found: C 69.4, H 8.08.

Analytical Data of 7. Yield: 7.5 mg (5.6 µmol, 80 %). Melting point: 143 – 148 °C

^{1}H NMR (400 MHz, C_6D_6) δ = 7.57 (s, 1H, Ar*H*, *para* to Bi), 7.35 – 7.30 (m, 4H, iPr$_2$Ar*H*), 7.26 – 7.18 (m, 8H, iPr$_2$Ar*H*), 7.01 (s, 2H, Me$_2$Ar*H*), 3.63 (d, $^{3}J_{HH}$=7.0 Hz, 3H, BiCH(C*H*$_3$)$_2$), 3.30 (sept, $^{3}J_{HH}$=6.8 Hz, 2H, ArC*H*(CH$_3$), 3.21 (sept, $^{3}J_{HH}$=6.8 Hz, 2H, ArC*H*(CH$_3$) 2.85 – 2.62

(m, 6 H, ArCH(CH$_3$)$_2$), 2.60 -2.49 (m, 1H BiCH(CH$_3$)$_2$), 2.39 (d, $^3J_{HH}$=7.0 Hz, 3H, BiCH(CH_3)$_2$), 2.06 (s, 6H, ArCH_3), 1.96 (s, 6H, ArCH_3), 1.35 (d, $^3J_{HH}$=6.9 Hz ArCH(CH_3)$_2$), 1.32 (d, $^3J_{HH}$=6.9 Hz ArCH(CH_3)$_2$), 1.23 – 1.17 (m, 30H ArCH(CH_3)$_2$), 1.16 – 1.09 (m, 18H ArCH(CH_3)$_2$) ppm.

^{13}C NMR (101 MHz, C_6D_6) δ = 150.0 (Ar), 147.3 (Ar), 146.9 (Ar), 146.7 (Ar), 139.2 (Ar), 138.9 (Ar), 138.4 (Ar), 138.3 (Ar), 135.4 (Ar), 132.1 (Ar), 124.1 (Ar), 123.6 (Ar), 123.6 (Ar), 123.2 (Ar), 123.2 (Ar), 58.9 (BiCH(CH$_3$)$_2$), 31.2 (ArCH(CH$_3$)$_2$), 31.2 (ArCH(CH$_3$)$_2$), 30.7 (ArCH(CH$_3$)$_2$), 30.7 (ArCH(CH$_3$)$_2$), 30.5 (ArCH(CH$_3$)$_2$), 25.6 (ArCH(CH$_3$)$_2$), 25.6 (ArCH(CH$_3$)$_2$), 24.8 (ArCH(CH$_3$)$_2$), 24.8 (ArCH(CH$_3$)$_2$), 24.3 (BiCH(CH$_3$)$_2$), 20.3 ((BiCH(CH$_3$)$_2$)) ppm.

ATR-IR: ν = 3061, 2959, 2923, 2867, 1574, 1465, 1386, 1362, 1323, 1249, 1179, 1106, 1052, 996, 899, 813, 802, 775, 752, 675, 619, 567, 441 cm^{-1}.

Elemental analysis calcd. for $C_{79}H_{104}BiBr$: C 70.7, H 7.51, found: C 69.8, H 8.47.

General Synthesis of 8-13. 7.0 µmol of **5** (9.5 mg), **6** (9.3 mg), or **7** (9.4 mg) were dissolved in 0.5 mL C_6D_6 and 10 µL of a 1 M solution of $LiAlH_4$ or $LiAlD_4$ in Et_2O were added. This resulted in immediate decolorization and *in situ* NMR spectroscopy showed complete conversion to a single product. The solutions were freeze-dried, dissolved in 0.5 ml benzene, filtered, and again freeze-dried, yielding the products in sufficient purity.

Unfortunately, meaningful elemental analyses of the bismuth hydrides/deuterides **8-13** were not obtained most likely due to the decomposition of the compounds, which was also observed during melting point measurements. The decomposition typically resulted in the formation of elemental bismuth, which was found as residues in all measurements.

Analytical Data of 8. Yield: 8.6 mg (6.5 µmol, 93 %). Melting point: gradually turning brown above 151 °C and grey above 280 °C.

^{1}H NMR (400 MHz, C_6D_6) δ = 11.5 (br s, 1H, BiH), 7.49 (s, 1H, ArH, *para* to Bi), 7.37 – 7.31 (m, 4H, iPr$_2$ArH), 7.25 – 7.19 (m, 8H, iPr$_2$ArH), 6.95 (s, 2H, Me$_2$ArH), 3.21 – 3.09 (m, 4H, ArCH(CH$_3$)$_2$), 2.96 – 2.79 (m, 6H, ArCH(CH$_3$)$_2$), 1.96 (s, 6H, ArCH_3), 1.88 (s, 6H, ArCH_3)1.31 – 1.12 (m, 63 H, ArCH(CH_3)$_2$, BiCH_3) ppm.

^{13}C NMR (101 MHz, C_6D_6) δ = 147.0 (Ar), 146.9 (Ar), 146.9 (Ar), 146.8 (Ar), 145.7 (Ar), 138.9 (Ar), 138.9 (Ar), 138.6 (Ar), 134.1 (Ar), 133.8 (Ar), 130.8 (Ar), 123.3 (Ar), 123.2 (Ar), 123.2 (Ar), 123.2 (Ar), 32.0 (ArCH(CH$_3$)$_2$), 31.0 (ArCH(CH$_3$)$_2$), 31.0 (ArCH(CH$_3$)$_2$), 30.9 (ArCH(CH$_3$)$_2$), 30.8 (ArCH(CH$_3$)$_2$), 25.5, 25.4, 25.2, 25.1, 24.6, 24.5, 24.4, 24.1, 24.0, 19.8, 19.7 ppm. The ^{13}C NMR signals between 25.5 ppm and 19.7 ppm belong to the ArCH(CH$_3$)$_2$ and BiCH$_3$ groups, but could not be assigned more accurately.

ATR-IR: ν = 3038, 2957, 2923, 2865, 1709, 1576, 1461, 1382, 1359, 1325, 1264, 1106, 1057, 1025, 937, 899, 799, 752, 678, 569, 447 cm^{-1}.

Analytical Data of 9. Yield: 7.5 mg (5.7 µmol, 81 %). Melting point: gradually turning brown above 149 °C and grey above 270 °C.

^{1}H NMR (400 MHz, C_6D_6) δ = 7.49 (s, 1H, ArH, *para* to Bi), 7.37 – 7.31 (m, 4H, iPr$_2$ArH), 7.25 – 7.19 (m, 8H, iPr$_2$ArH), 6.95 (s, 2H, Me$_2$ArH), 3.21 – 3.09 (m, 4H, ArCH(CH$_3$)$_2$), 2.96 – 2.79 (m, 6H, ArCH(CH$_3$)$_2$), 1.96 (s, 6H, ArCH_3), 1.88 (s, 6H, ArCH_3)1.31 – 1.12 (m, 63 H, ArCH(CH_3)$_2$, BiCH_3) ppm.

^{2}H NMR (61 MHz, C_6H_6) δ = 11.56 (s, BiD) ppm.

^{13}C NMR (101 MHz, C_6D_6) δ = 147.0 (Ar), 146.9 (Ar), 146.9 (Ar), 146.8 (Ar), 145.7 (Ar), 138.9 (Ar), 138.9 (Ar), 138.6 (Ar), 134.1 (Ar), 133.8 (Ar), 130.8 (Ar), 123.3 (Ar), 123.2 (Ar),

123.2 (Ar), 123.2 (Ar), 32.0 (Ar*C*H(CH_3)$_2$), 31.0 (Ar*C*H(CH_3)$_2$), 31.0 (Ar*C*H(CH_3)$_2$), 30.9 (Ar*C*H(CH_3)$_2$), 30.8 (Ar*C*H(CH_3)$_2$), 25.5, 25.4, 25.2, 25.1, 24.6, 24.5, 24.4, 24.1, 24.0, 19.8, 19.7 ppm. The ^{13}C NMR signals between 25.5 ppm and 19.7 ppm belong to the ArCH(*C*H_3)$_2$, Ar*C*H_3 and Bi*C*H_3 groups, but could not be assigned more accurately.

ATR-IR: ν = 3063, 2957, 2926, 2865, 1576, 1459, 1380, 1359, 1321, 1226, 1104, 1055, 899, 815, 799, 754, 675, 569, 443 cm^{-1}.

Analytical Data of 10. Yield: 7.2 mg (5.8 μmol, 83 %). Melting point: turns black above 120 °C

^{1}H NMR (400 MHz, C_6D_6) δ = 11.97 (br s, 1H, Bi*H*), 7.51 (s, 1H, Ar*H*, *para* to Bi), 7.37 – 7.31 (m, 4H, iPr$_2$Ar*H*), 7.26 – 7.19 (m, 8H, iPr$_2$Ar*H*), 6.96 (s, 2H, Me$_2$Ar*H*), 3.23 – 3.08 (m, 4H, ArC*H*(CH_3)$_2$),2.96 – 2.79 (m, 7H, ArC*H*(CH_3)$_2$, BiC*H*HCH$_3$), 2.75 – 2.64 (m, 1H, BiCH-*H*CH$_3$), 2.18 (t, 3H, $^3J_{HH}$=7.8 Hz, BiCHHC*H*$_3$), 2.00 (s, 6H, ArC*H*$_3$), 1.90 (s, 6H, ArC*H*$_3$), 1.32 (d, 6H, $^3J_{HH}$=6.8 Hz, ArCH(C*H*$_3$)$_2$), 1.29 – 1.13 (m, 54H, overlapped by impurities, ArCH(C*H*$_3$)$_2$) ppm.

^{13}C NMR (101 MHz, C_6D_6) δ = 146.9 (Ar), 146.8 (Ar), 146.8 (Ar), 146.8 (Ar), 146.4 (Ar), 146.3 (Ar), 146.0 (Ar), 144.9 (Ar), 139.0 (Ar), 138.9 (Ar), 138.8 (Ar), 138.6 (Ar), 134.4 (Ar), 133.9 (Ar), 130.9 (Ar), 123.3 (Ar), 123.2 (Ar), 123.2 (Ar), 31.8 (Ar*C*H(CH_3)$_2$), 31.1 (Ar*C*H(CH_3)$_2$), 31.0 (Ar*C*H(CH_3)$_2$), 30.8 (Ar*C*H(CH_3)$_2$), 30.8 (Ar*C*H(CH_3)$_2$), 25.5, 25.4, 25.3, 25.1, 24.6, 24.5, 24.3, 24.2, 24.1, 19.7 (Ar*C*H_3), 19.6 (Ar*C*H_3), 18.9, (BiCH$_2$*C*H$_3$), 18.3 ppm. We could not identify the Bi*C*H$_2$CH$_3$ ^{13}C NMR signal. The other unassigned signals are those of the ArCH(*C*H_3)$_2$ groups.

ATR-IR: ν = 2957, 2926, 2867, 1705, 1574, 1463, 1380, 1359, 1323, 1258, 1102, 1052, 1023, 901, 797, 752, 569, 497, 447 cm^{-1}.

Analytical Data of 11. Yield: 8.4 mg (6.7 μmol, 96 %). Melting point: turns black above 120 °C

^{1}H NMR (400 MHz, C_6D_6) δ = 7.51 (s, 1H, Ar*H*, *para* to Bi), 7.37 – 7.31 (m, 4H, iPr$_2$Ar*H*), 7.26 – 7.19 (m, 8H, iPr$_2$Ar*H*), 6.96 (s, 2H, Me$_2$Ar*H*), 3.23 – 3.08 (m, 4H, ArC*H*(CH_3)$_2$),2.96 – 2.79 (m, 7H, ArC*H*(CH_3)$_2$, BiC*H*HCH$_3$), 2.75 – 2.64 (m, 1H, BiCH*H*CH$_3$), 2.18 (t, 3H, $^3J_{HH}$=7.8 Hz, BiCHHC*H*$_3$), 2.00 (s, 6H, ArC*H*$_3$), 1.90 (s, 6H, ArC*H*$_3$), 1.32 (d, 6H, $^3J_{HH}$=6.8 Hz, ArCH(C*H*$_3$)$_2$), 1.29 – 1.13 (m, 54H, overlapped by impurities, ArCH(C*H*$_3$)$_2$) ppm.

^{2}H NMR (61 MHz, C_6H_6) δ = 12.04 (s, Bi*D*) ppm.

^{13}C NMR (101 MHz, C_6D_6) δ = 146.9 (Ar), 146.8 (Ar), 146.8 (Ar), 146.8 (Ar), 146.4 (Ar), 146.3 (Ar), 146.0 (Ar), 144.9 (Ar), 139.0 (Ar), 138.9 (Ar), 138.8 (Ar), 138.6 (Ar), 134.4 (Ar), 133.9 (Ar), 130.9 (Ar), 123.3 (Ar), 123.2 (Ar), 123.2 (Ar), 31.8 (Ar*C*H(CH_3)$_2$), 31.1 (Ar*C*H(CH_3)$_2$), 31.0 (Ar*C*H(CH_3)$_2$), 30.8 (Ar*C*H(CH_3)$_2$), 30.8 (Ar*C*H(CH_3)$_2$), 25.5, 25.4, 25.3, 25.1, 24.6, 24.5, 24.3, 24.2, 24.1, 19.7 (Ar*C*H_3), 19.6 (Ar*C*H_3), 18.9, (BiCH$_2$*C*H$_3$), 18.3 ppm. We could not identify the Bi*C*H$_2$CH$_3$ ^{13}C NMR signal. The other unassigned signals are those of the ArCH(*C*H_3)$_2$ groups.

ATR-IR: ν = 2959, 2926, 2862, 1580, 1463, 1382, 1359, 1321, 1251, 1219, 1104, 1057, 991, 944, 897, 802, 752, 673, 621, 567, 499, 452 cm^{-1}.

Analytical Data of 12. Yield: 8.7 mg (6.9 μmol, 98 %). Melting point: gradually turning brown above 131 °C

^{1}H NMR (400 MHz, C_6D_6) δ = 13.33 (br s, 1H, Bi*H*), 7.51 (s, 1H, Ar*H*, *para* to Bi), 7.38 – 7.31 (m, 4H, iPr$_2$Ar*H*), 7.27 – 7.19 (m, 8H, iPr$_2$Ar*H*), 6.96 (s, 2H, Me$_2$Ar*H*), 3.22 – 3.05 (m, 7H, ArC*H*(CH_3)$_2$, BiCH(C*H*$_3$)(CH_3)), 2.95 – 2.76 (m, 6H, ArC*H*(CH_3)$_2$), 2.42 – 2.29 (m, 1H, BiC*H*(CH_3)(CH_3)), 2.01 (s, 6H, ArC*H*$_3$), 1.91 (s, 6H, ArC*H*$_3$), 1.68 (d, 3H, $^3J_{HH}$=7.3 Hz,

BiCH(CH_3)(CH_3)), 1.35 (d, 6H, $^3J_{HH}$=6.9 Hz, ArCH(CH_3)$_2$) 1.29 -1.12(m, 54H, overlapped by impurities, ArCH(CH_3)$_2$) ppm.

^{13}C NMR (101 MHz, C_6D_6) δ =146.9 (Ar), 146.8 (Ar), 146.7 (Ar), 146.7 (Ar), 146.4 (Ar), 145.8 (Ar), 139.0 (Ar), 138.8 (Ar), 138.6 (Ar), 134.6 (Ar), 133.8 (Ar), 130.9 (Ar), 123.3 (Ar), 123.2 (Ar), 123.2 (Ar), 33.8 (BiCH(*C*H_3)(CH_3), Ar*C*H(CH_3)$_2$), 31.8 (Ar*C*H(CH_3)$_2$), 31.2 (Ar*C*H(CH_3)$_2$), 30.9 (Ar*C*H(CH_3)$_2$), 30.8 (Ar*C*H(CH_3)$_2$), 27.1 (Bi*C*H(CH_3)(CH_3), only observed in HSQC and HMBC spectra) 25.6 (ArCH(*C*H_3)$_2$), 25.6 (ArCH(*C*H_3)$_2$), 25.4 (ArCH(*C*H_3)$_2$), 25.2 (ArCH(*C*H_3)$_2$), 24.6 (BiCH(*C*H_3)(CH_3)), 24.5 (ArCH(*C*H_3)$_2$), 24.2 (ArCH(*C*H_3)$_2$), 24.1 (ArCH(*C*H_3)$_2$), 19.9 (Ar*C*H_3), 19.8 (Ar*C*H_3) ppm.

ATR-IR: ν = 2959, 2923, 2869, 1693, 1461, 1380, 1362, 1323, 1262, 1174, 1136, 1097, 1057, 989, 901, 813, 799, 754, 567, 452, 436 cm^{-1}.

Analytical Data of 13. Yield: 7.7 mg (6.1 µmol, 87 %). Melting point: gradually turning brown above 131 °C

^{1}H NMR (400 MHz, C_6D_6) δ = 7.51 (s, 1H, Ar*H*, *para* to Bi), 7.38 – 7.31 (m, 4H, iPr$_2$Ar*H*), 7.27 – 7.19 (m, 8H, iPr$_2$Ar*H*), 6.96 (s, 2H, Me$_2$Ar*H*), 3.22 – 3.05 (m, 7H, ArC*H*(CH_3)$_2$, BiCH(CH_3)(CH_3)), 2.95 – 2.76 (m, 6H, ArC*H*(CH_3)$_2$), 2.42 – 2.29 (m, 1H, BiC*H*(CH_3)(CH_3)), 2.01 (s, 6H, ArCH_3), 1.91 (s, 6H, ArCH_3), 1.68 (d, 3H, $^3J_{HH}$=7.3 Hz, BiCH(CH_3)(CH_3)), 1.35 (d, 6H, $^3J_{HH}$=6.9 Hz, ArCH(CH_3)$_2$) 1.29 -1.12(m, 54H, overlapped by impurities, ArCH(CH_3)$_2$) ppm.

^{2}H NMR (61 MHz, C_6H_6) δ = 13.36 (s, Bi*D*) ppm.

^{13}C NMR (101 MHz, C_6D_6) δ =146.9 (Ar), 146.8 (Ar), 146.7 (Ar), 146.7 (Ar), 146.4 (Ar), 145.8 (Ar), 139.0 (Ar), 138.8 (Ar), 138.6 (Ar), 134.6 (Ar), 133.8 (Ar), 130.9 (Ar), 123.3 (Ar), 123.2 (Ar), 123.2 (Ar), 33.8 (BiCH(*C*H_3)(CH_3), Ar*C*H(CH_3)$_2$), 31.8 (Ar*C*H(CH_3)$_2$), 31.2 (Ar*C*H(CH_3)$_2$), 30.9 (Ar*C*H(CH_3)$_2$), 30.8 (Ar*C*H(CH_3)$_2$), 27.1 (Bi*C*H(CH_3)(CH_3), only observed in HSQC and HMBC spectra) 25.6 (ArCH(*C*H_3)$_2$), 25.6 (ArCH(*C*H_3)$_2$), 25.4 (ArCH(*C*H_3)$_2$), 25.2 (ArCH(*C*H_3)$_2$), 24.6 (BiCH(*C*H_3)(CH_3)), 24.5 (ArCH(*C*H_3)$_2$), 24.2 (ArCH(*C*H_3)$_2$), 24.1 (ArCH(*C*H_3)$_2$), 19.9 (Ar*C*H_3), 19.8 (Ar*C*H_3) ppm.

ATR-IR: ν = 3063, 2955, 2926, 2865, 1578, 1463, 1384, 1357, 1323, 1262, 1215, 1172, 1134, 1104, 1052, 1023, 903, 799, 752, 565, 445, 429 cm^{-1}.

II. Spectroscopic Data

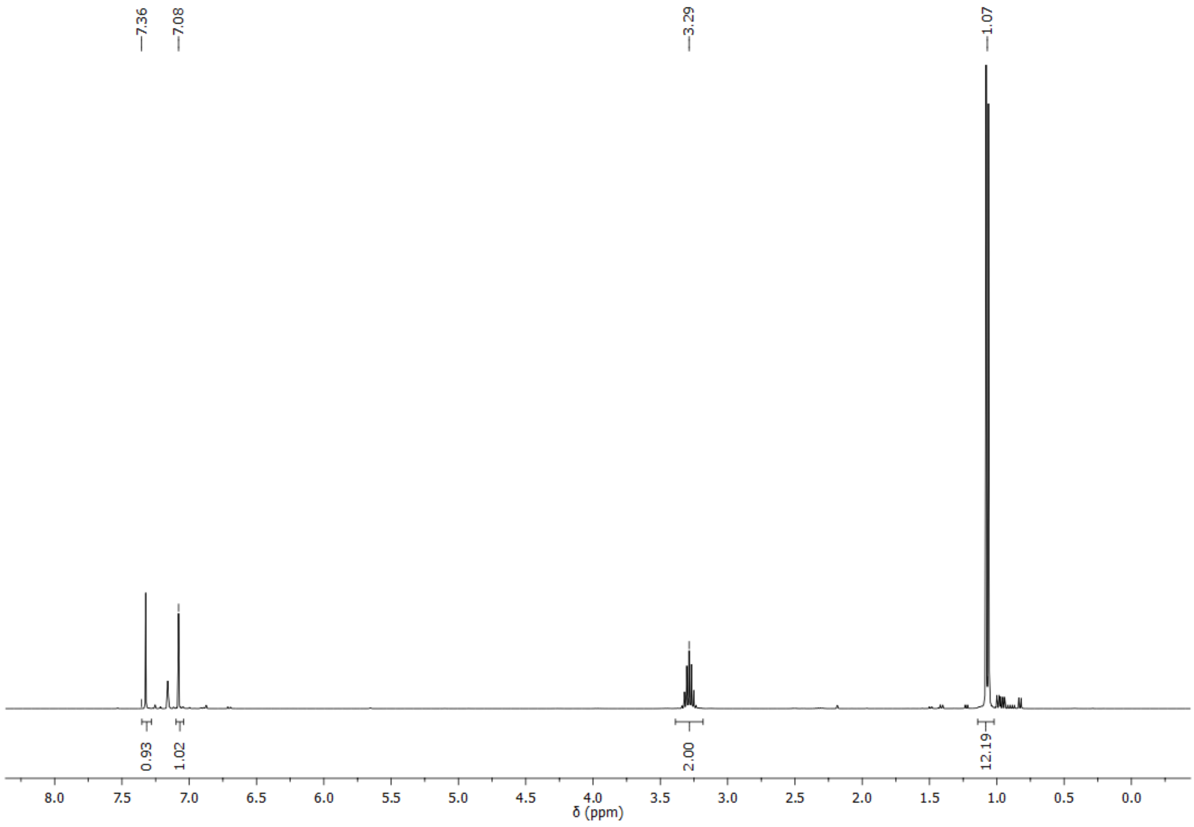

Figure S2: ^{1}H NMR spectrum of **Ar*-2** in C_6D_6 at 25 °C.

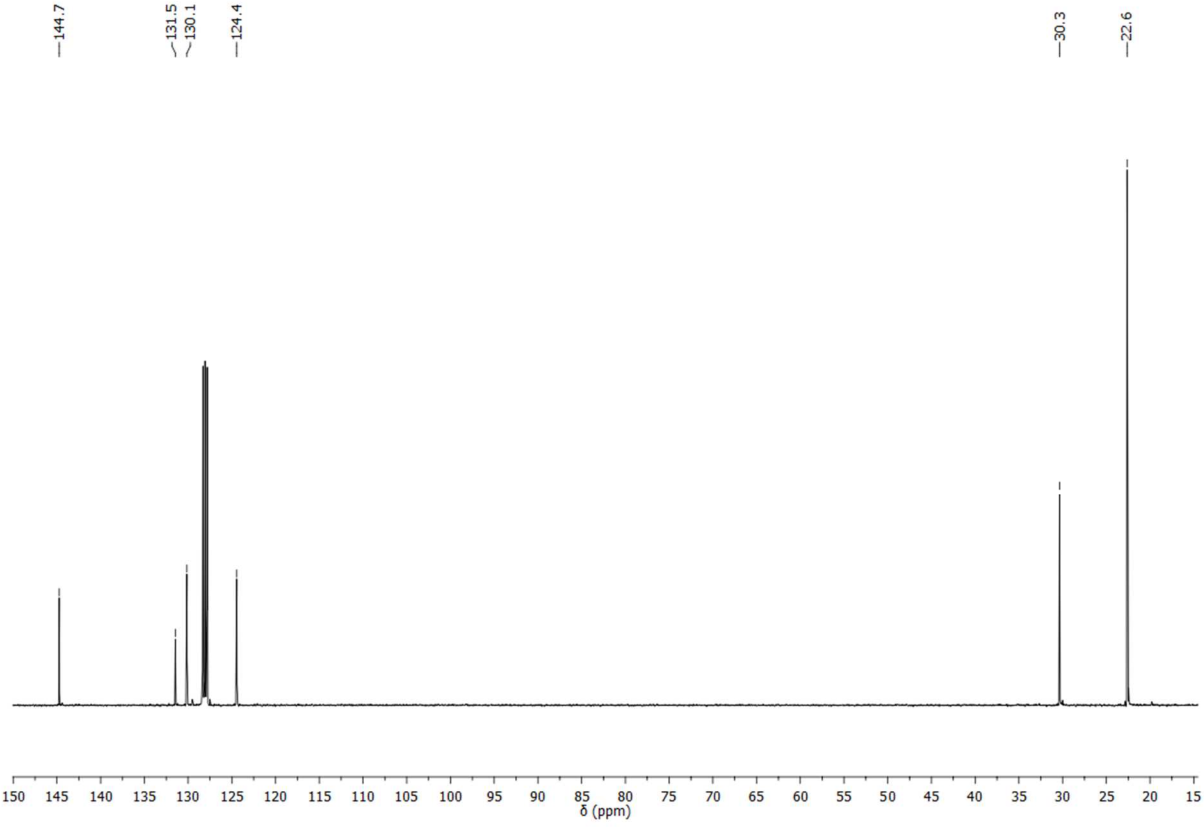

Figure S3: ^{13}C NMR spectrum of **Ar*-2** in C_6D_6 at 25 °C.

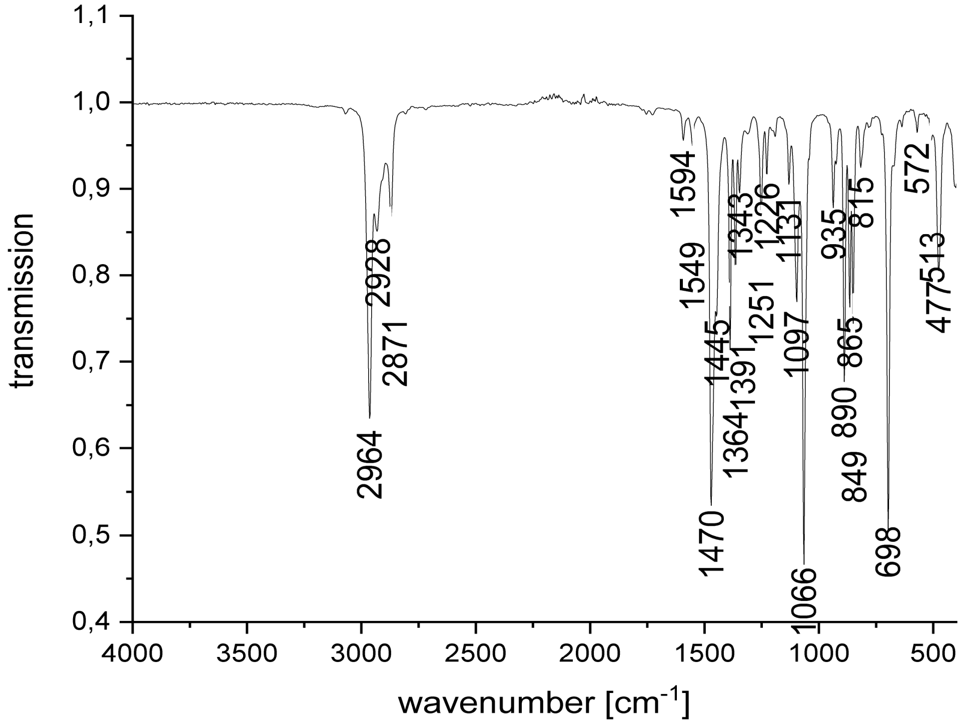

Figure S4: ATR-IR spectrum of **Ar*-2**.

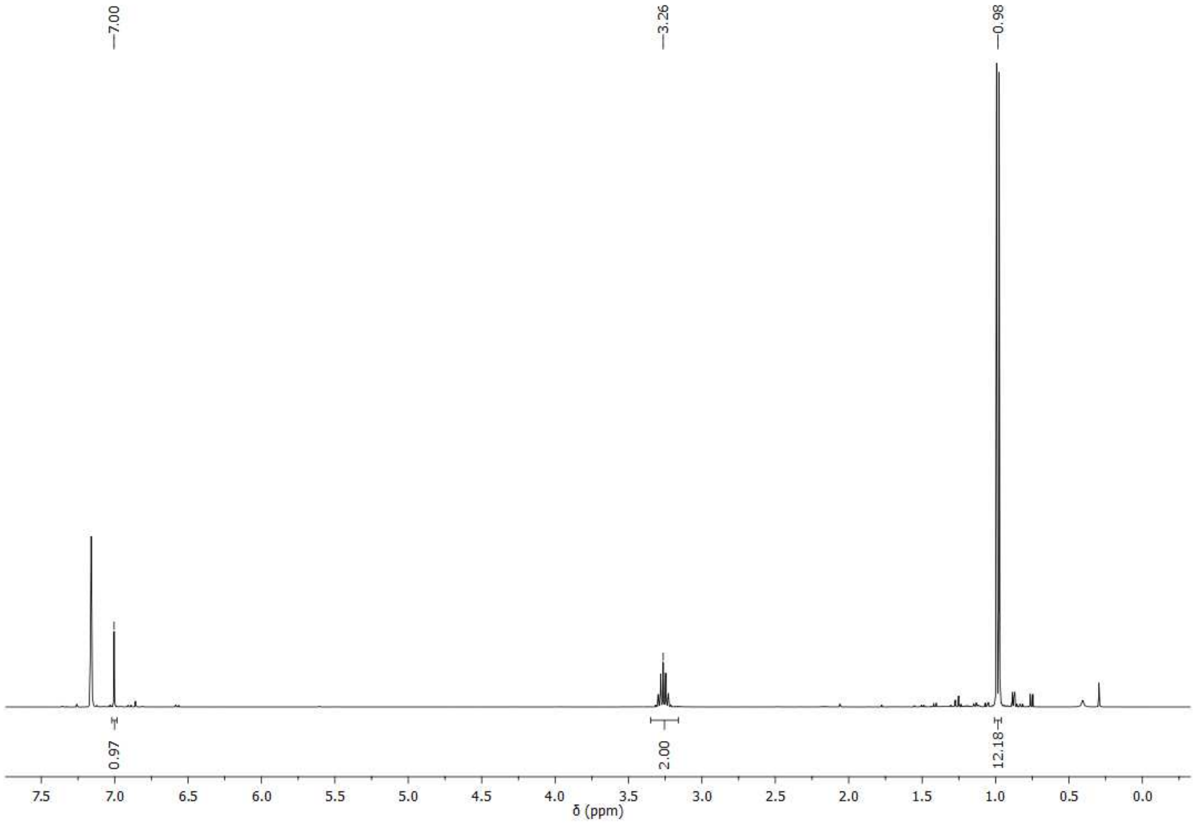

Figure S5: ^{1}H NMR spectrum of **Ar*-3** in C_6D_6 at 25 °C.

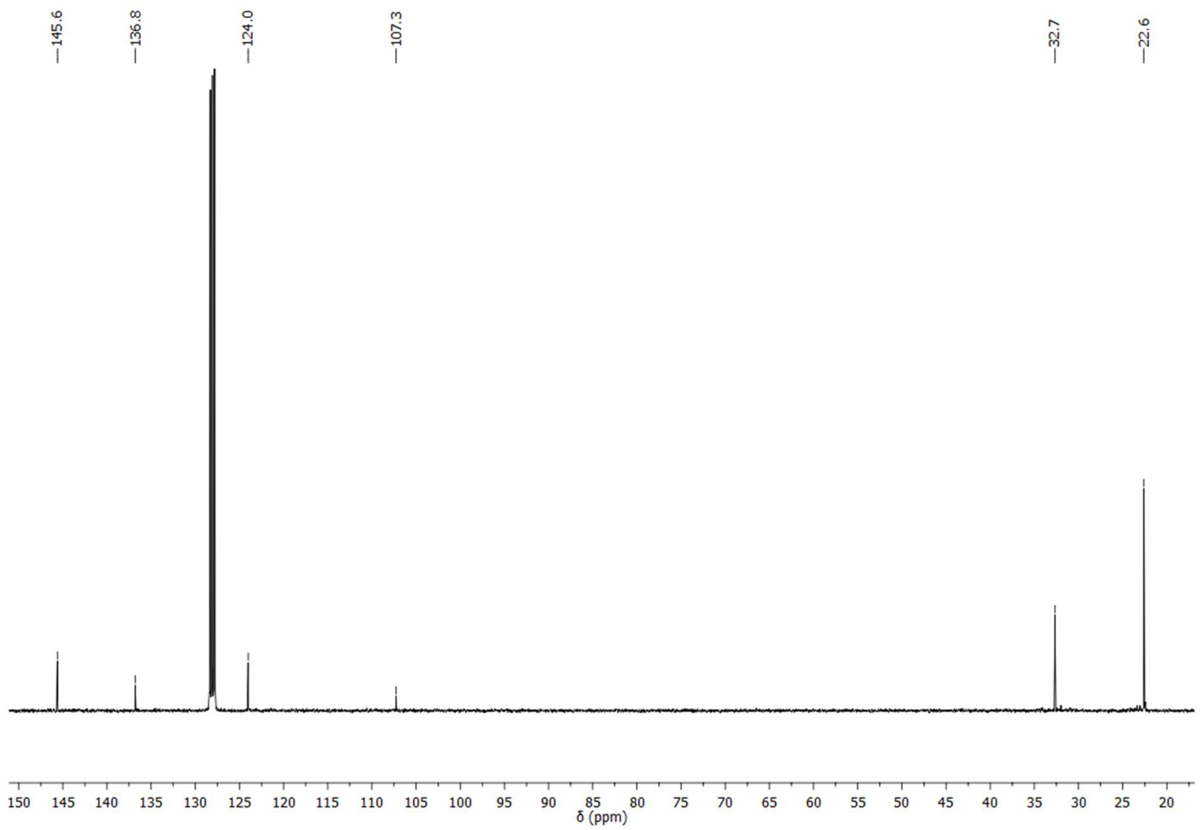

Figure S6: ^{13}C NMR spectrum of **Ar*-3** in C_6D_6 at 25 °C.

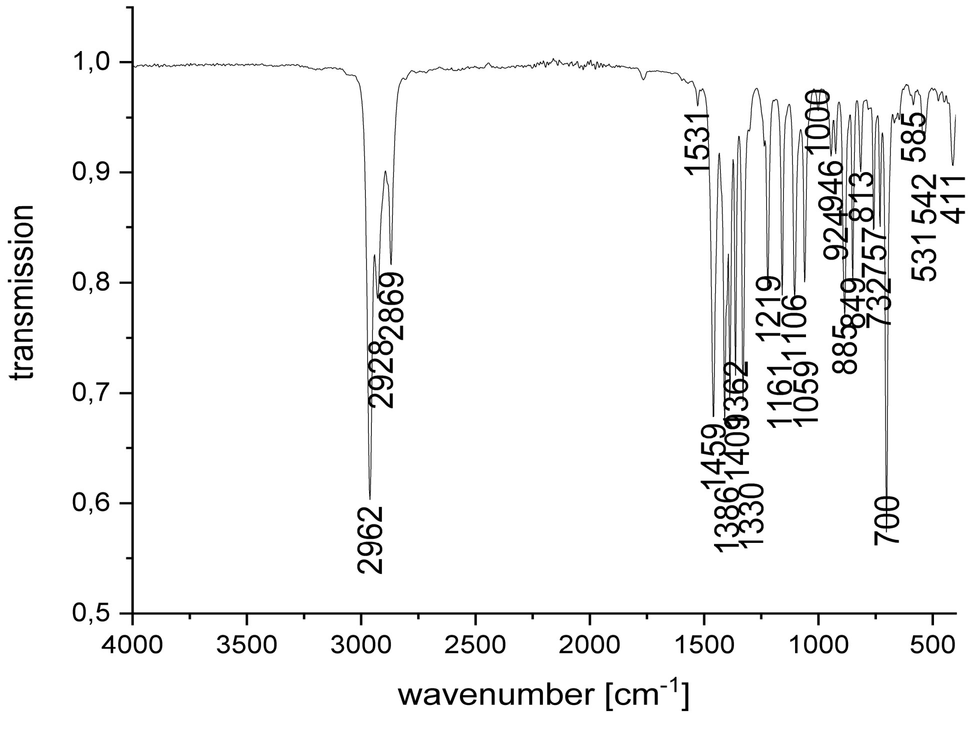

Figure S7: ATR-IR spectrum of **Ar*-3**.

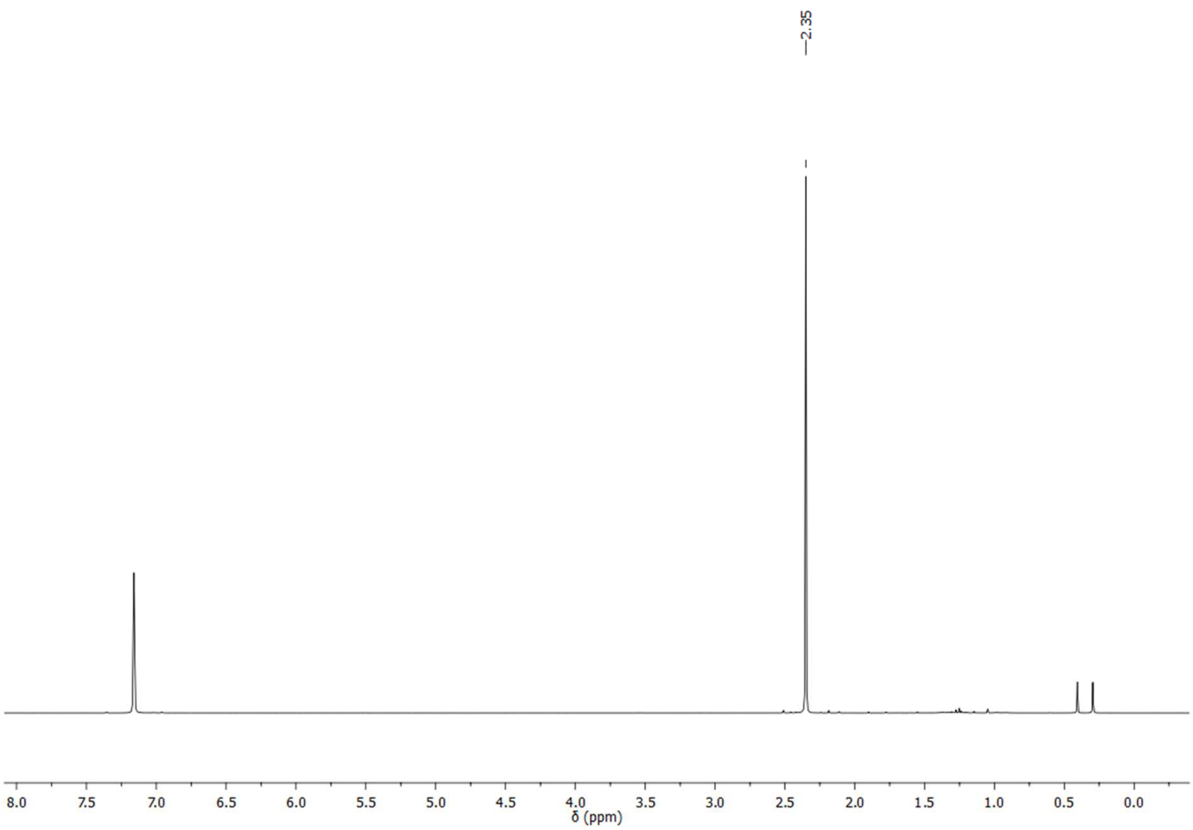

Figure S8: ^{1}H NMR spectrum of **Ar*-5** in C_6D_6 at 25 °C.

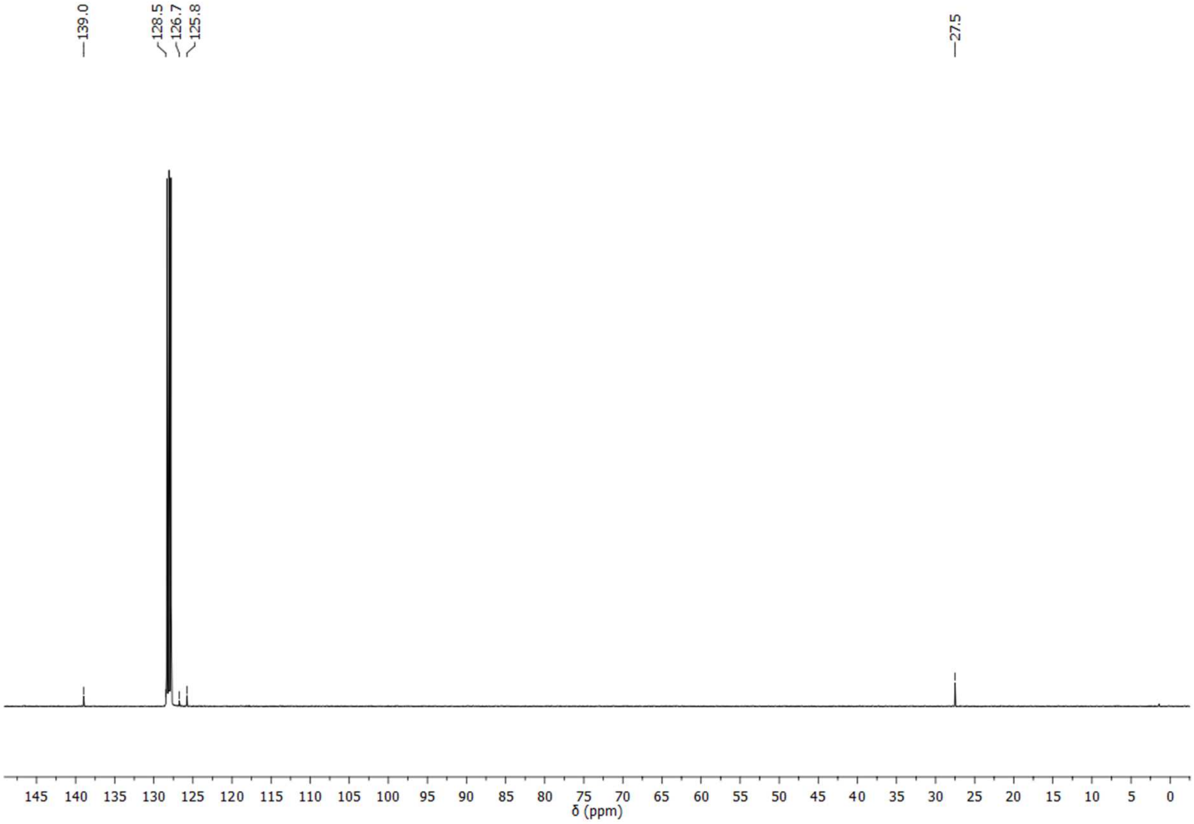

Figure S9: ^{13}C NMR spectrum of **Ar*-5** in C_6D_6 at 25 °C.

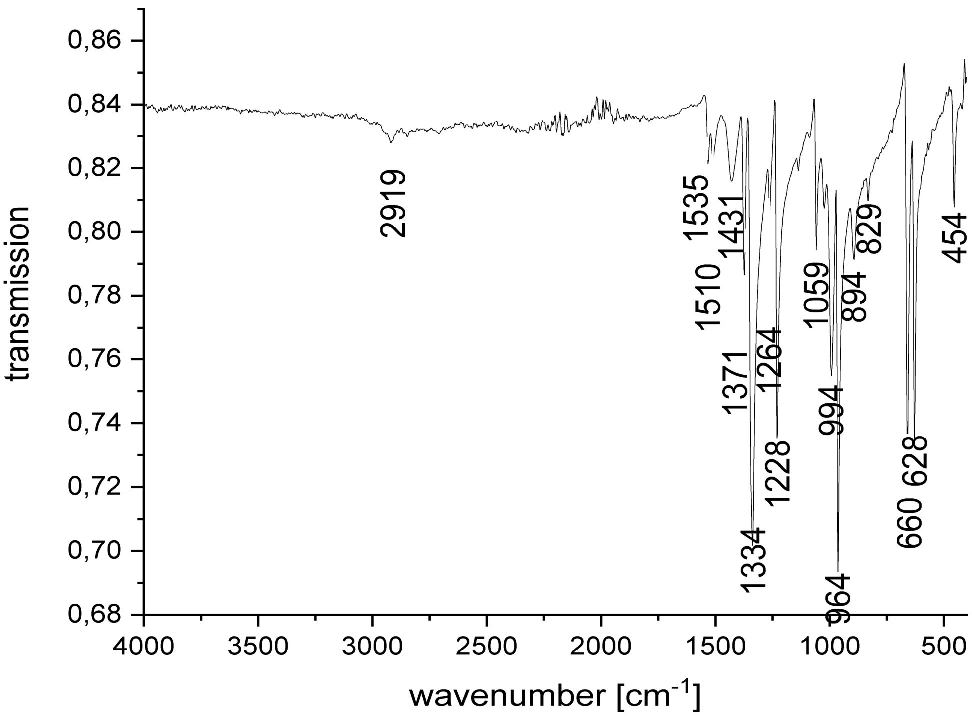

Figure S10: ATR-IR spectrum of **Ar*-5**.

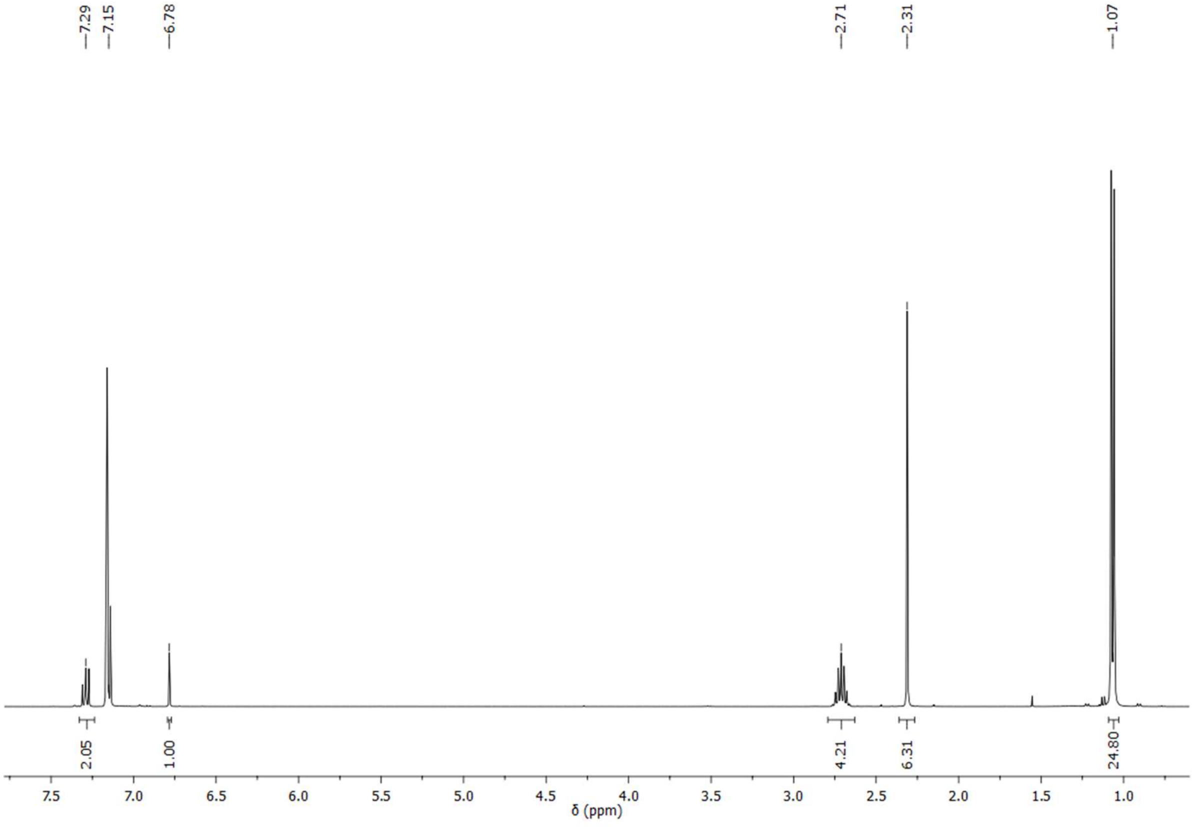

Figure S11: ^{1}H NMR spectrum of **Ar*-7** in C_6D_6 at 25 °C.

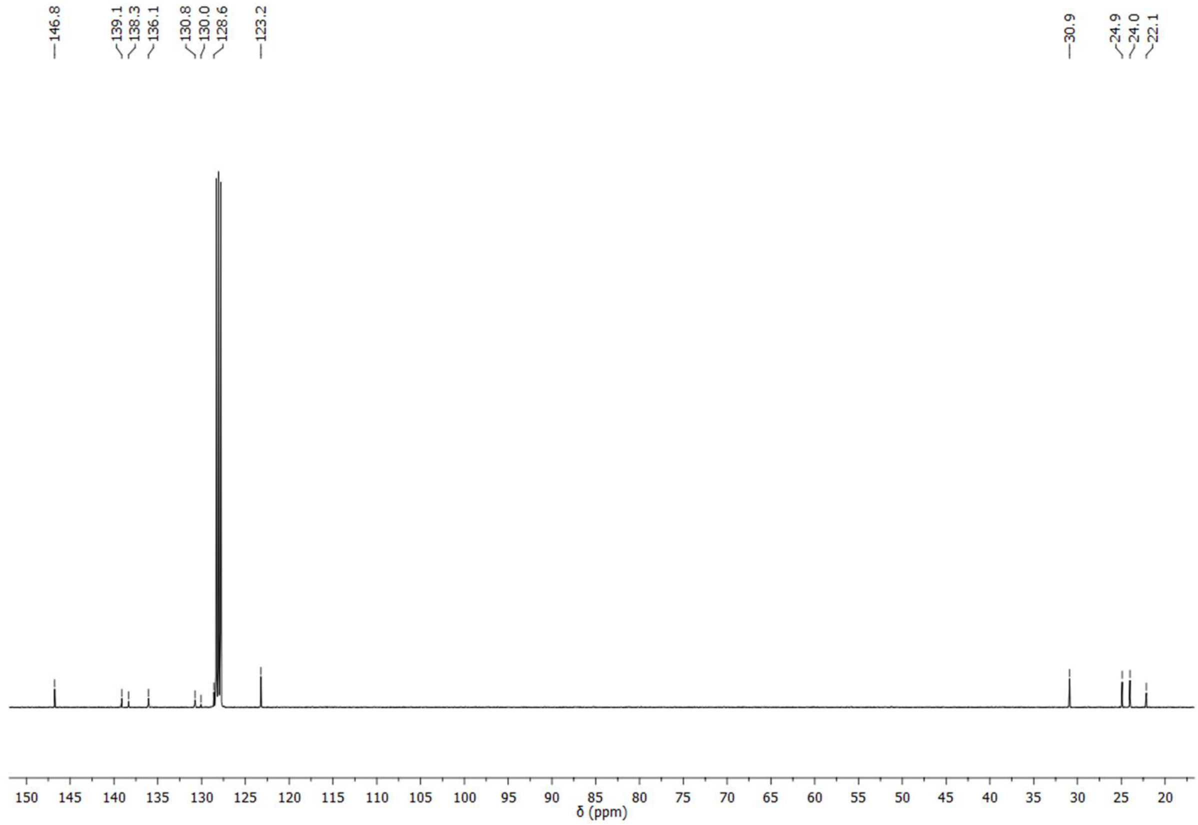

Figure S12: ^{13}C NMR spectrum of **Ar*-7** in C_6D_6 at 25 °C.

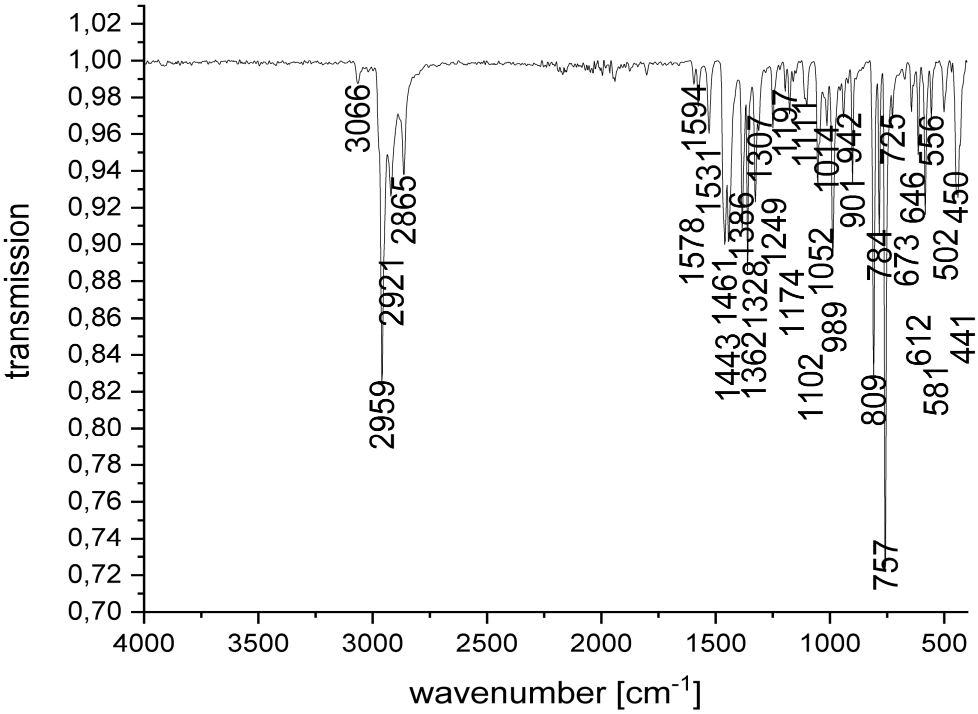

Figure S13: ATR-IR spectrum of **Ar*-7**.

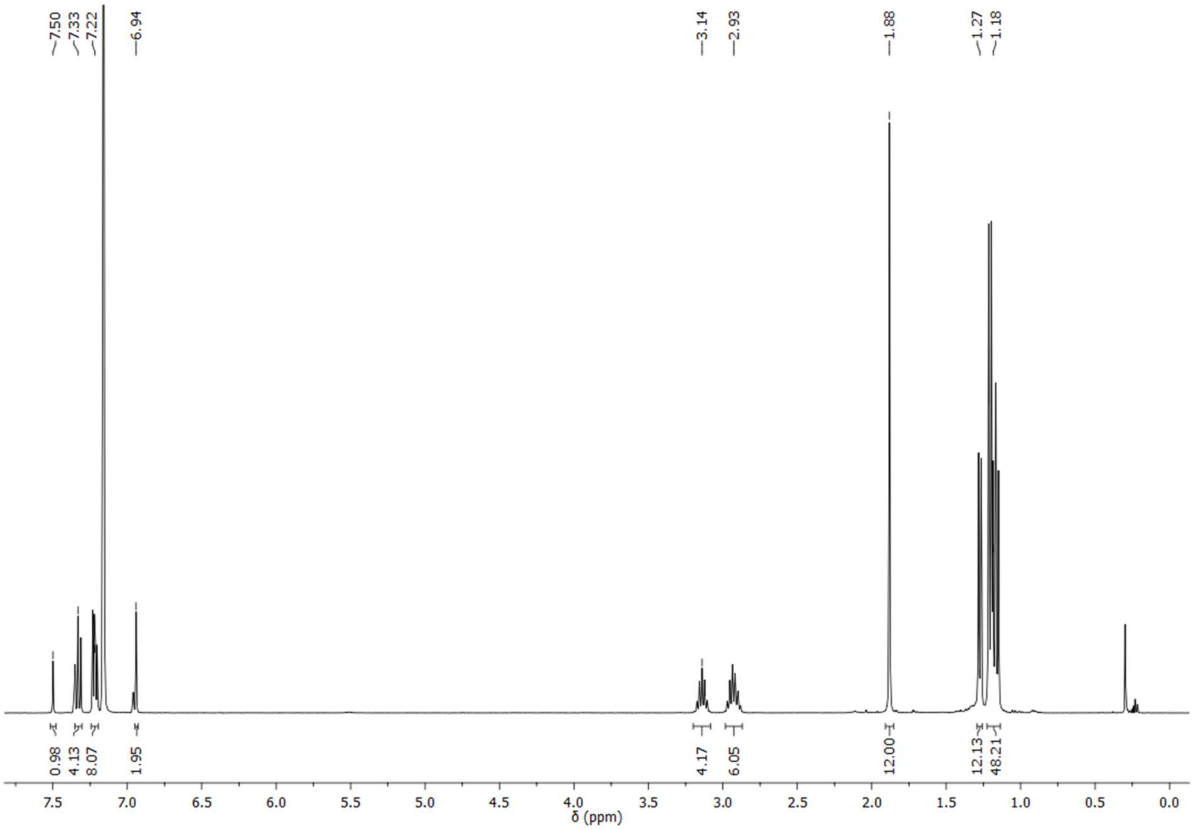

Figure S14: ^{1}H NMR spectrum of **Ar*I** in C_6D_6 at 25 °C.

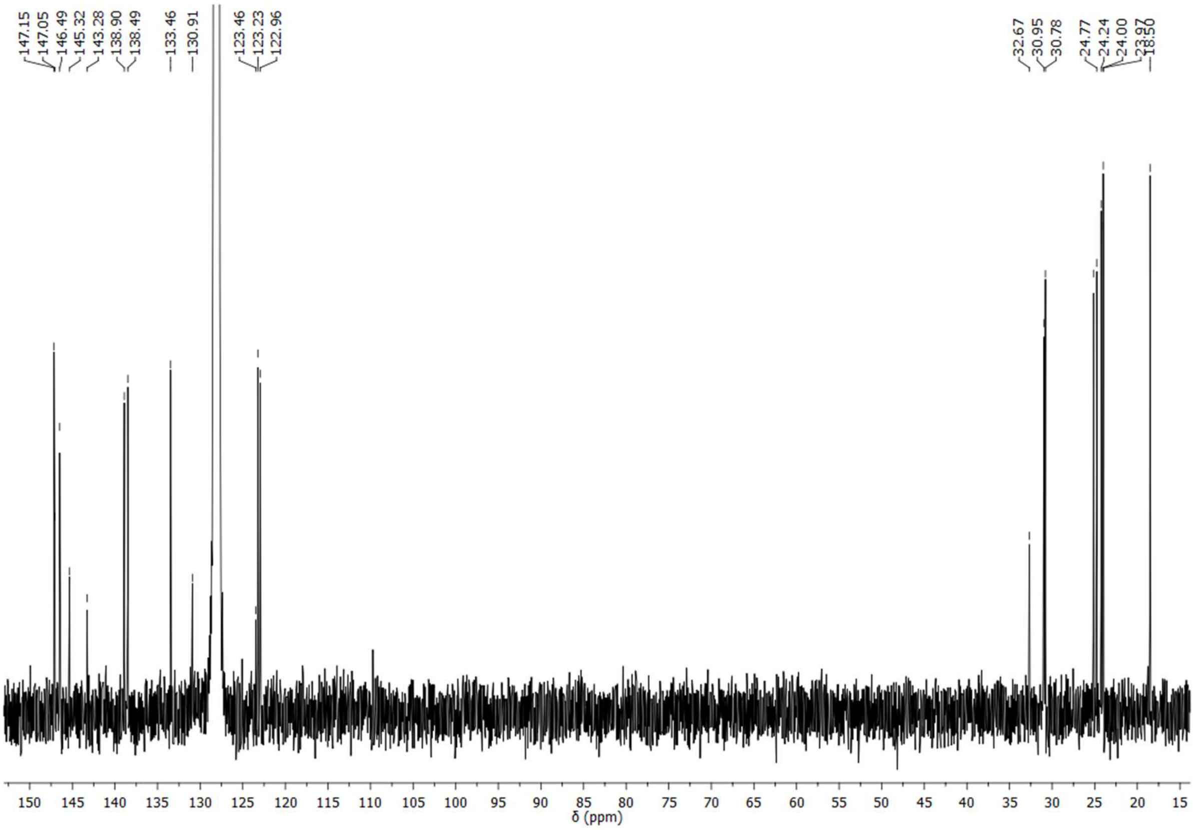

Figure S15: ^{13}C NMR spectrum of **Ar*I** in C_6D_6 at 25 °C.

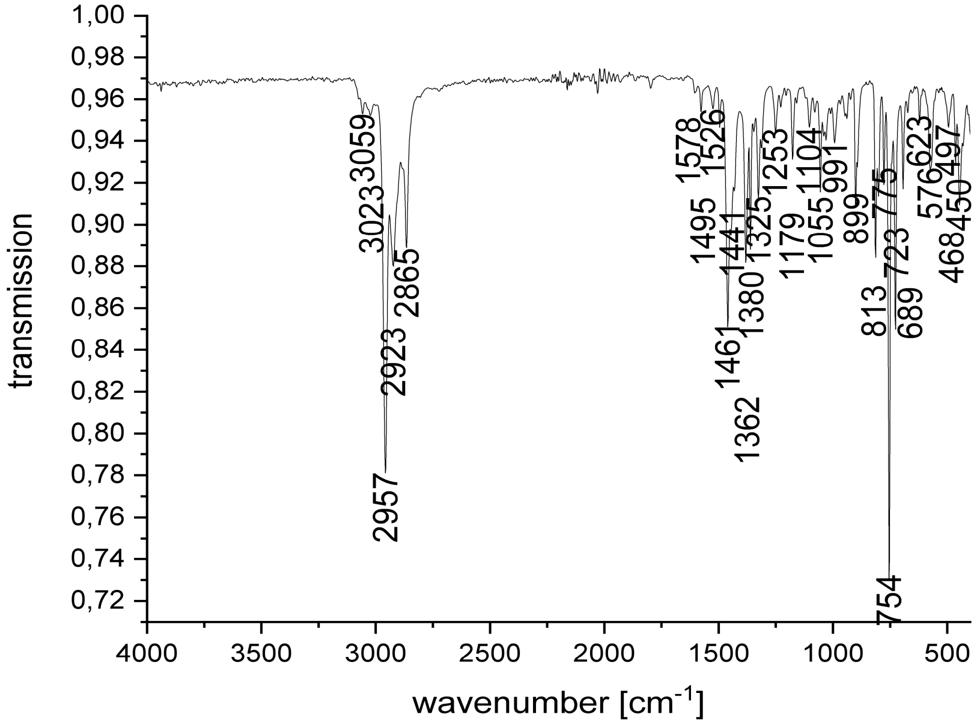

Figure S16: ATR-IR spectrum of **Ar*I**.

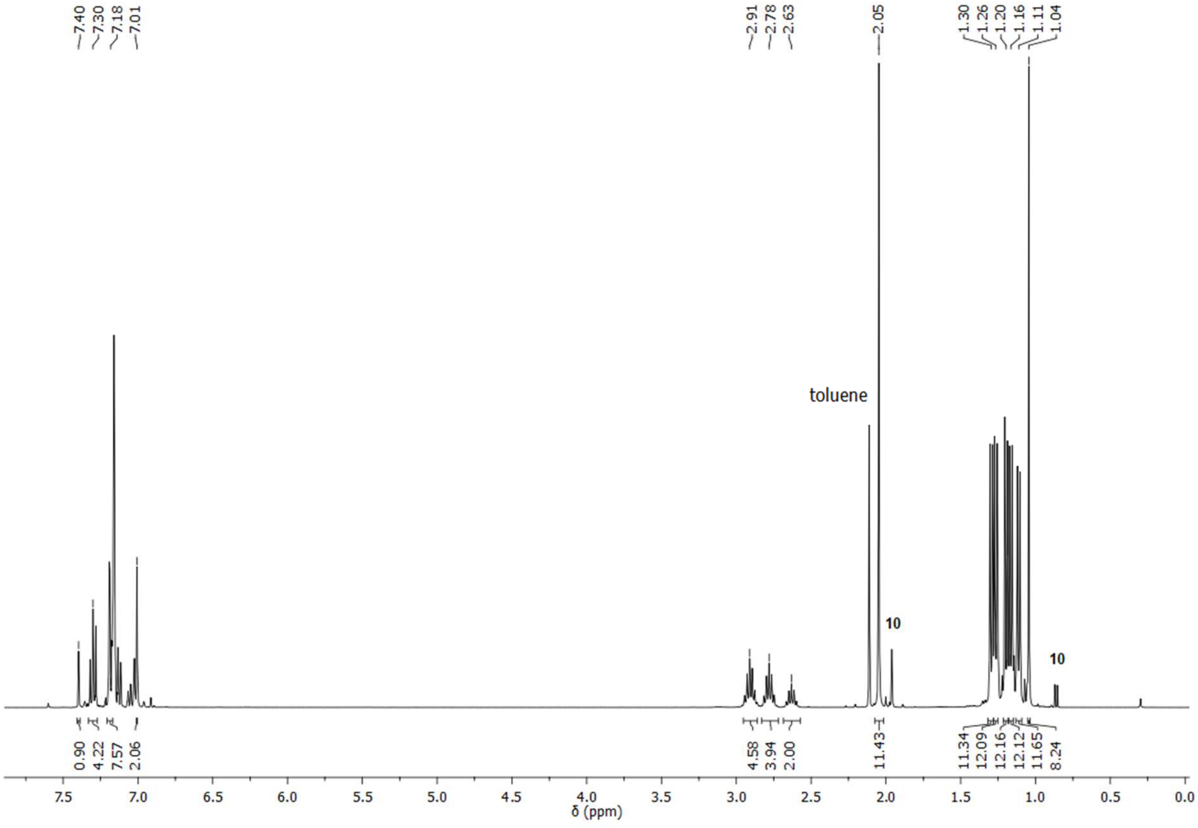

Figure S17: ^{1}H NMR spectrum of **Ar*Li·Li*t*-Bu** in C_6D_6 at 25 °C.

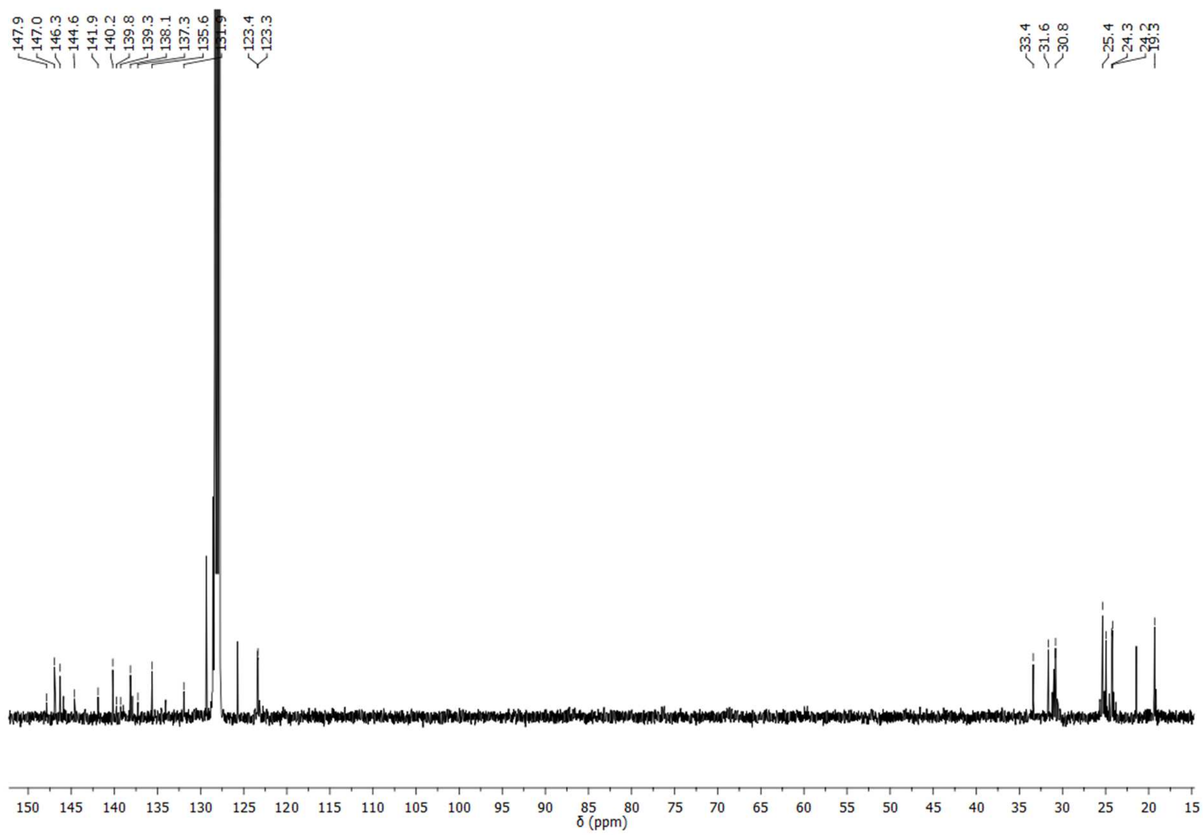

Figure S18: ^{13}C NMR spectrum of **Ar*Li·Li*t*-Bu** in C_6D_6 at 25 °C The peaks of toluene (137.9, 129.3, 128.6, 125.7, 21.4 ppm) and of **Ar*H** (*vide infra*) are not assigned here.

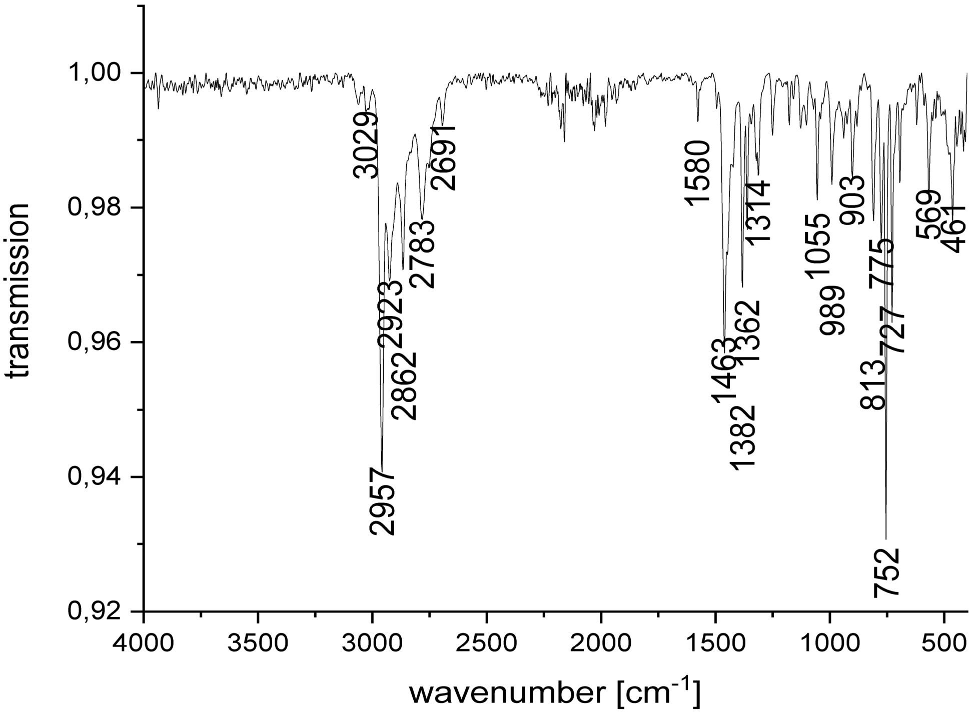

Figure S19: ATR-IR spectrum of **Ar*Li·Li*t*-Bu**.

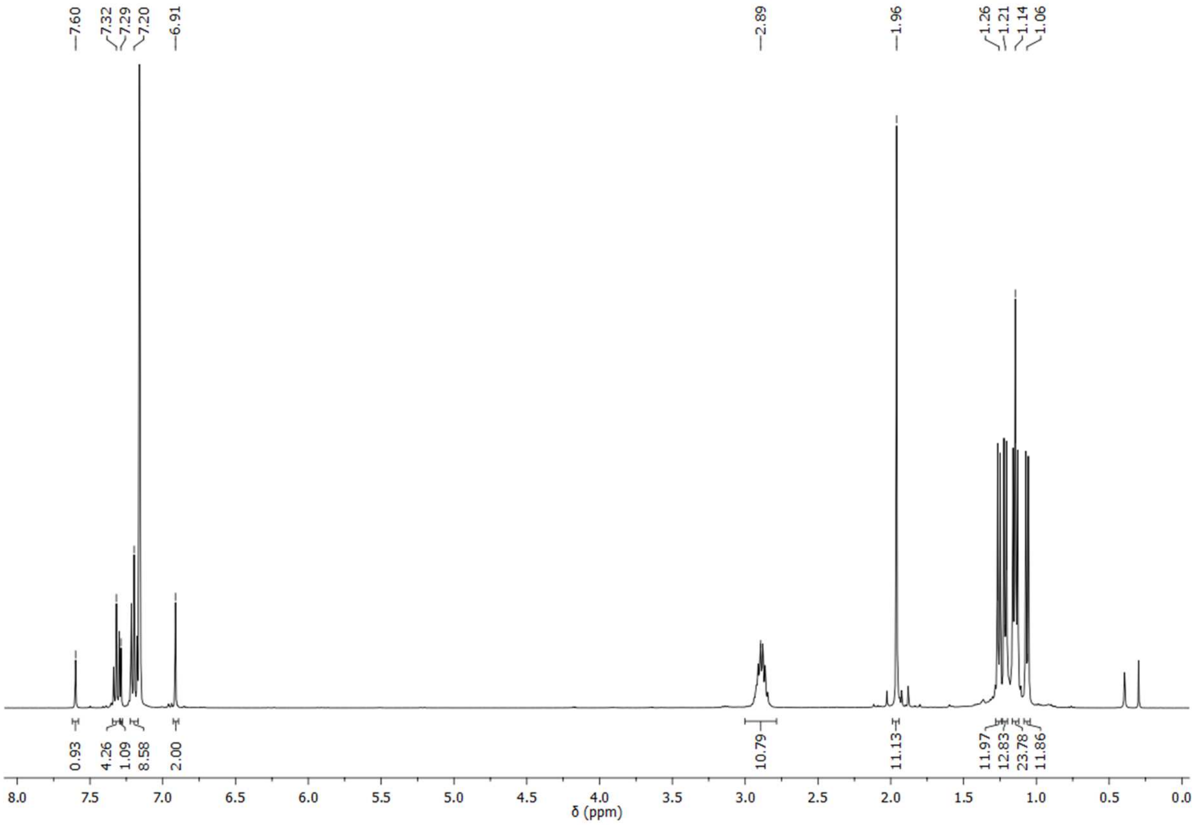

Figure S20: ^{1}H NMR spectrum of **Ar*H** in C_6D_6 at 25 °C.

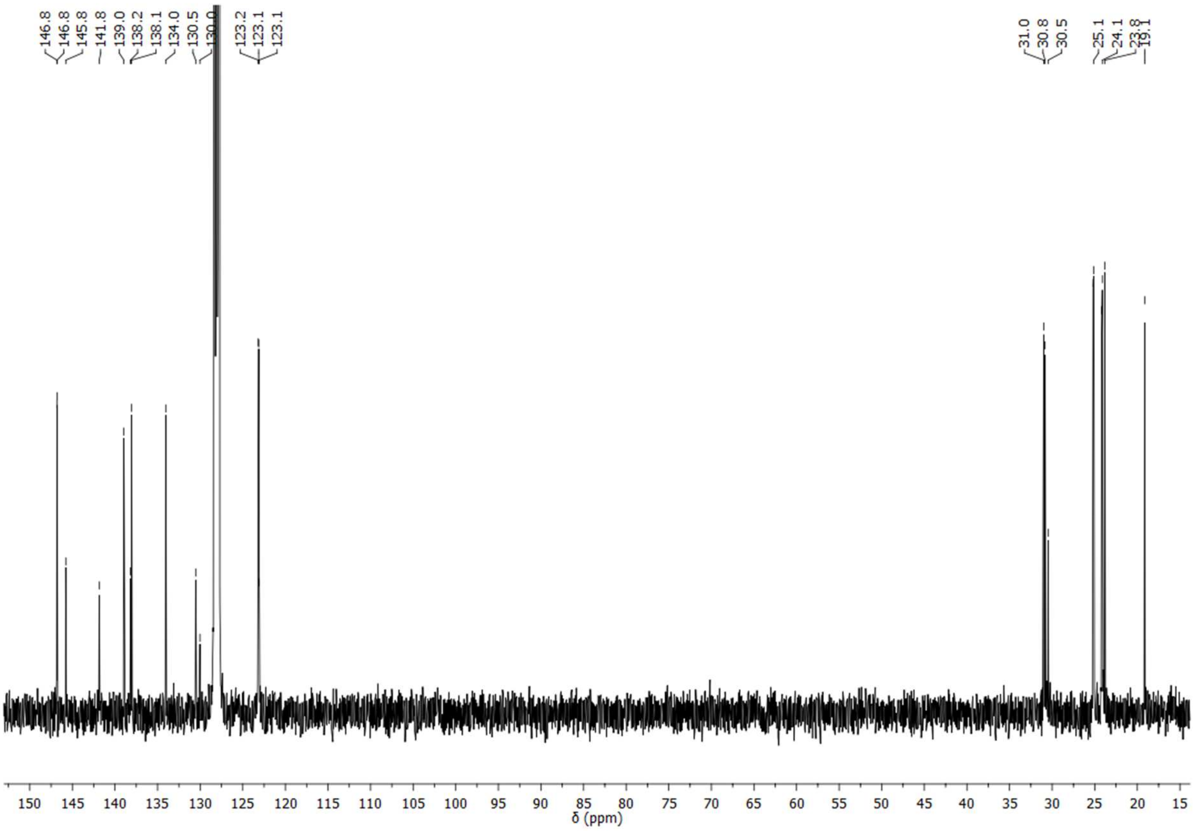

Figure S21: ^{13}C NMR spectrum of **Ar*H** in C_6D_6 at 25 °C.

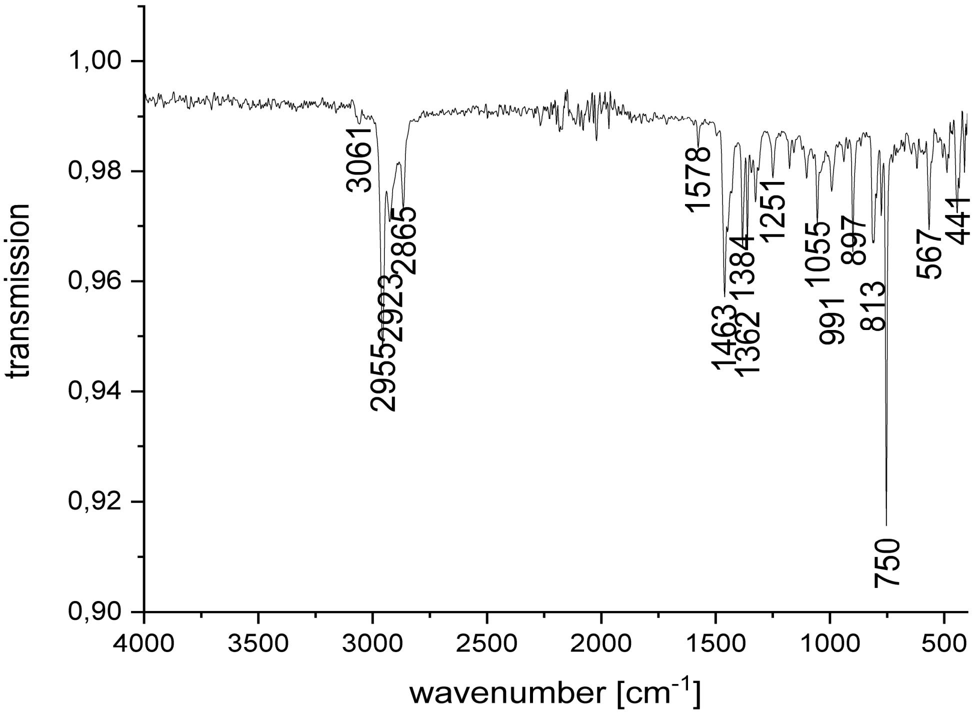

Figure S22: ATR-IR spectrum of **Ar*H**.

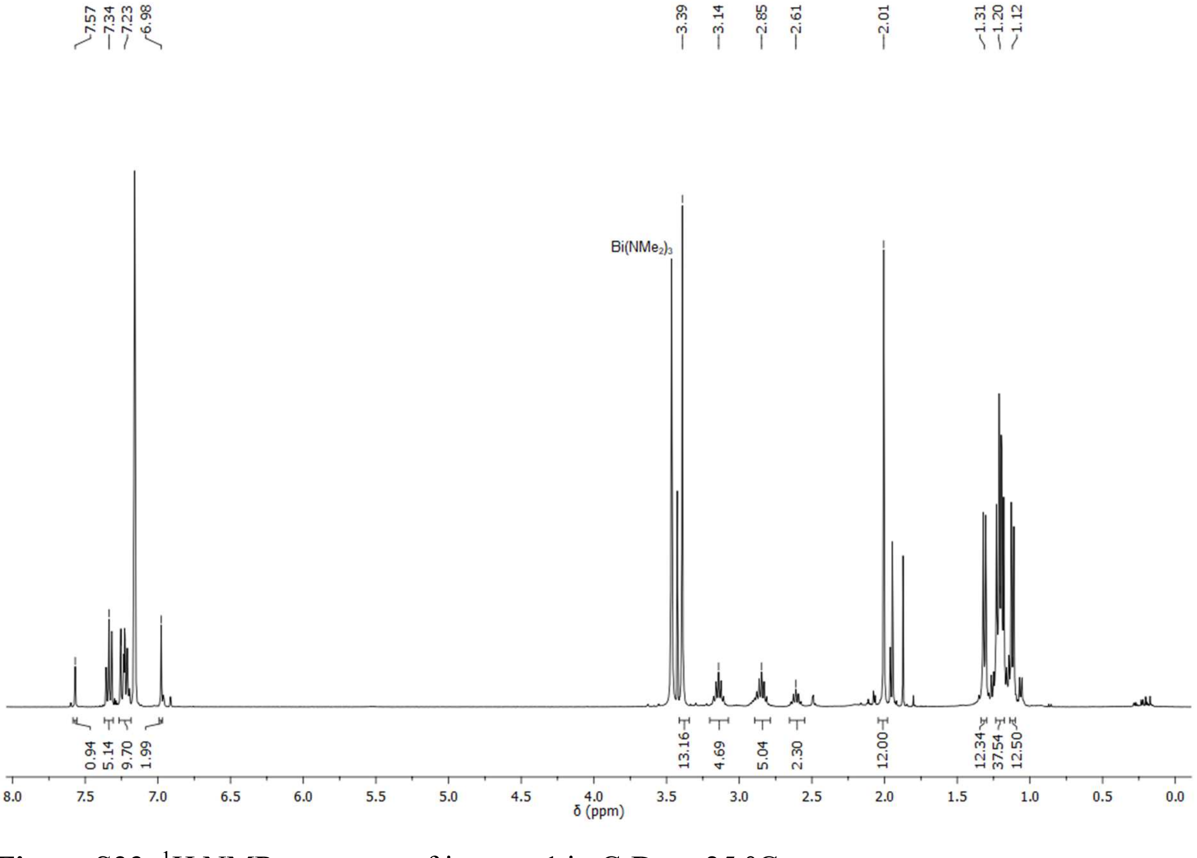

Figure S23: ^{1}H NMR spectrum of impure **1** in C_6D_6 at 25 °C.

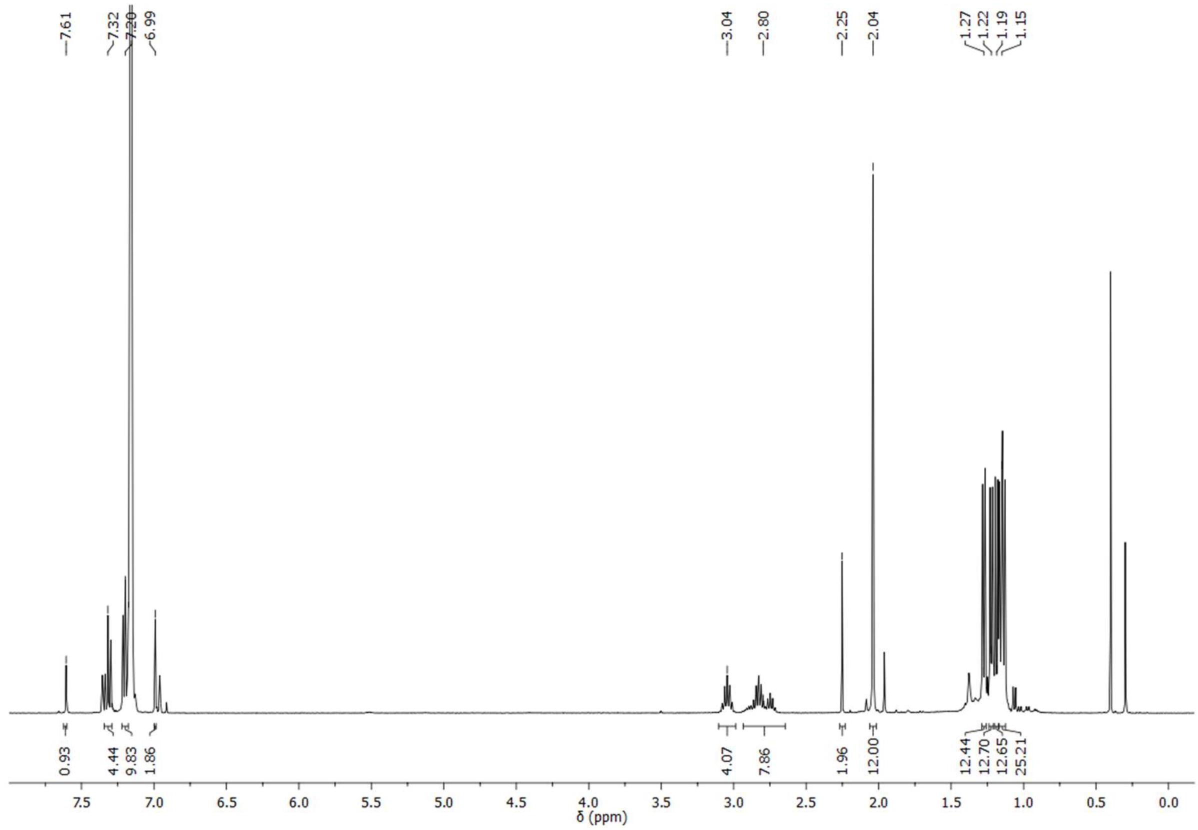

Figure S24: ^{1}H NMR spectrum of **2** in C_6D_6 at 25 °C.

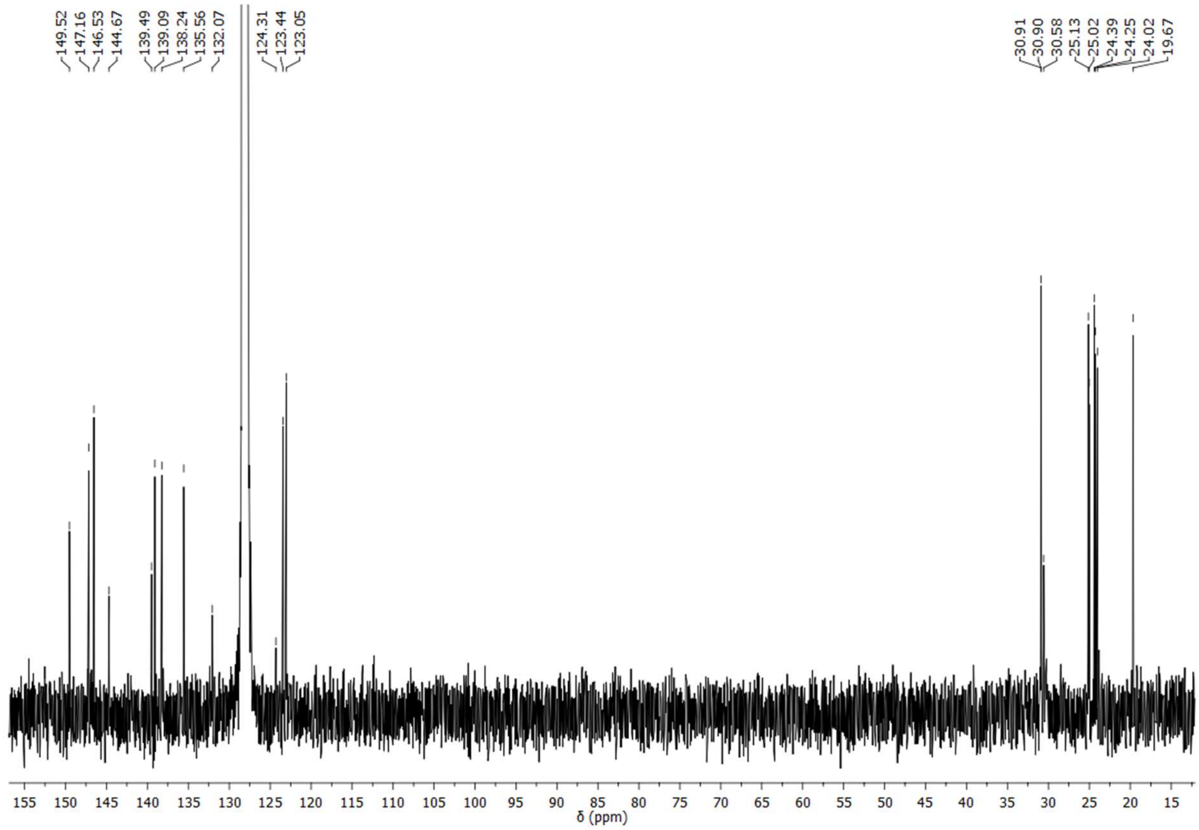

Figure S25: ^{13}C NMR spectrum of **2** in C_6D_6 at 25 °C.

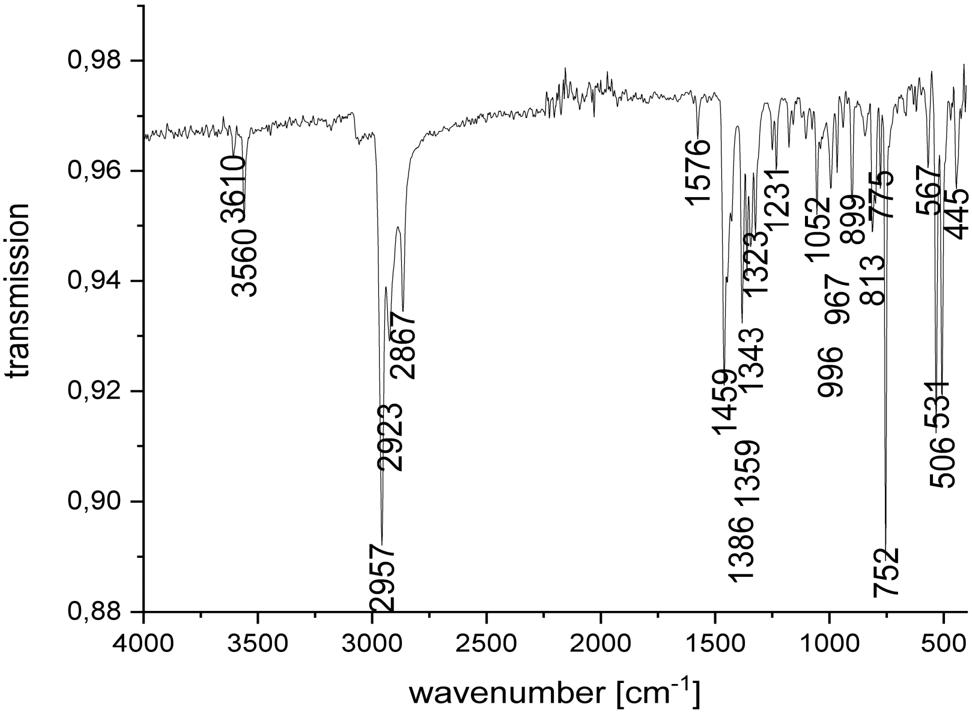

Figure S26: ATR-IR spectrum of **2**.

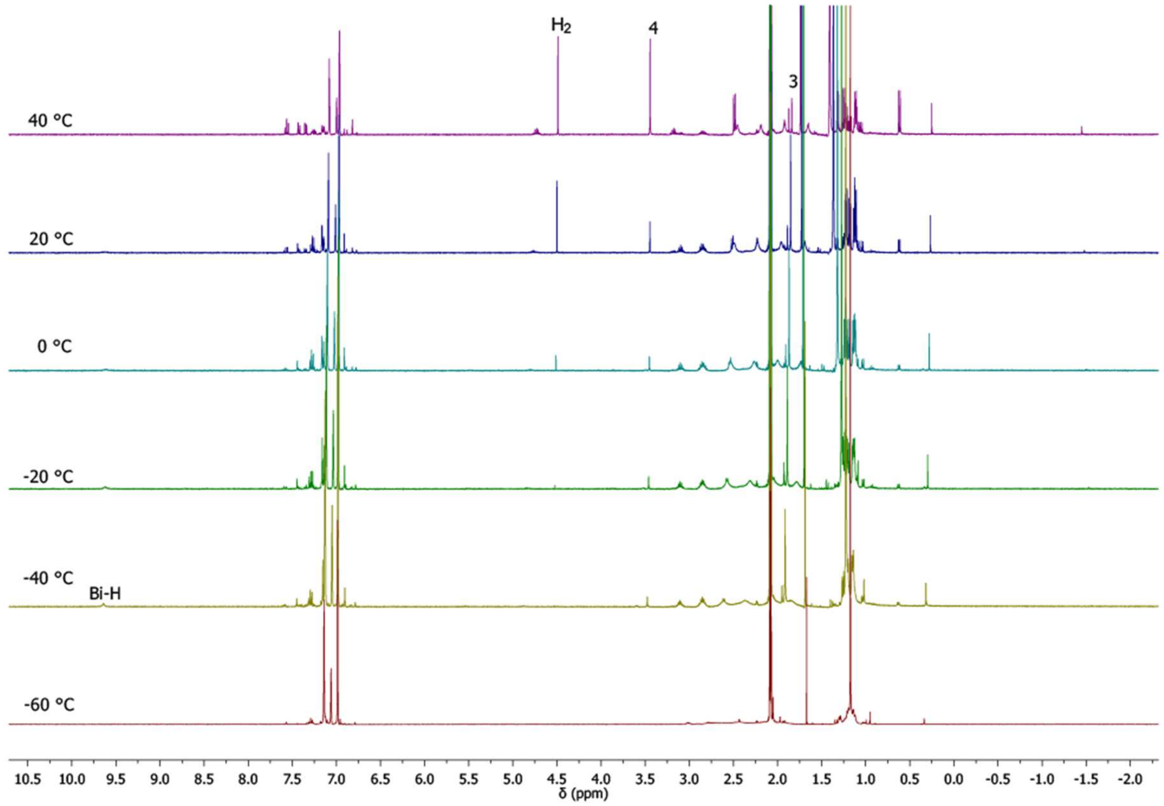

Figure S27: ^{1}H NMR spectra recorded at different temperatures, showing the conversion of **3** to **4** with elimination of H_2.

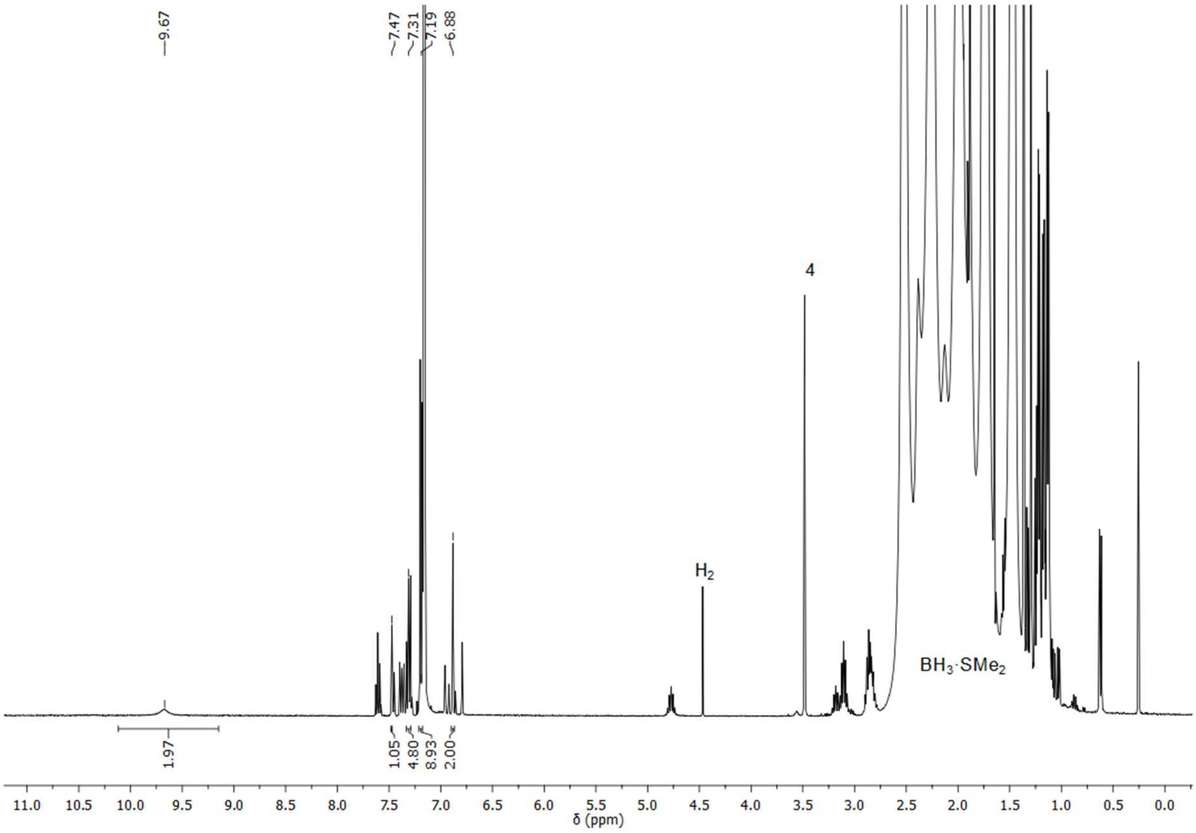

Figure S28: ^{1}H NMR spectrum of the reaction between **2** and $BH_3{\cdot}SMe_2$ after approx. 5 min at 25 °C, showing the intermediate formation of **3**, already accompanied by **4**.

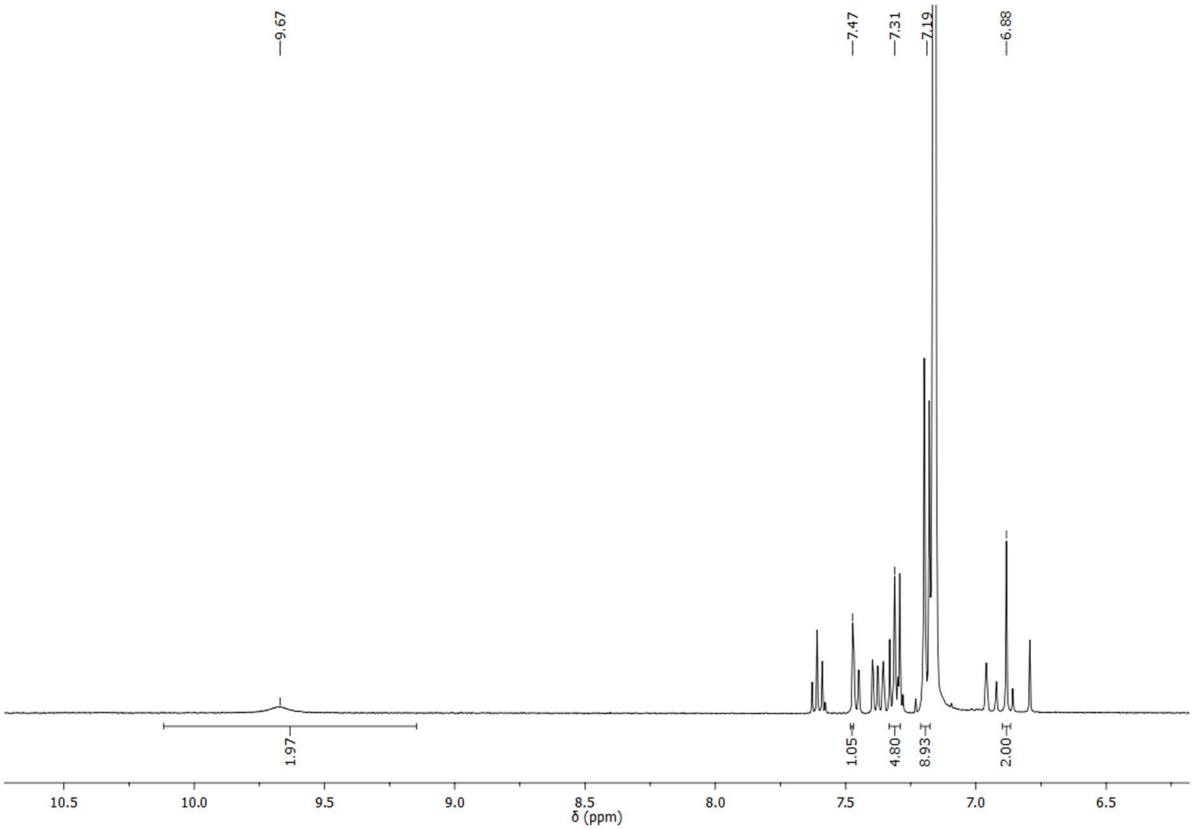

Figure S29: Low-field section of the ^{1}H NMR spectrum of **3** (excerpt from the same spectrum as in Figure S28). The unassigned signals are those of **4** which already formed by thermal decomposition of **3**. The high-field section was too badly resolved due to the presence of a large excess of $BH_3 \cdot SMe_2$ to be of analytical value.

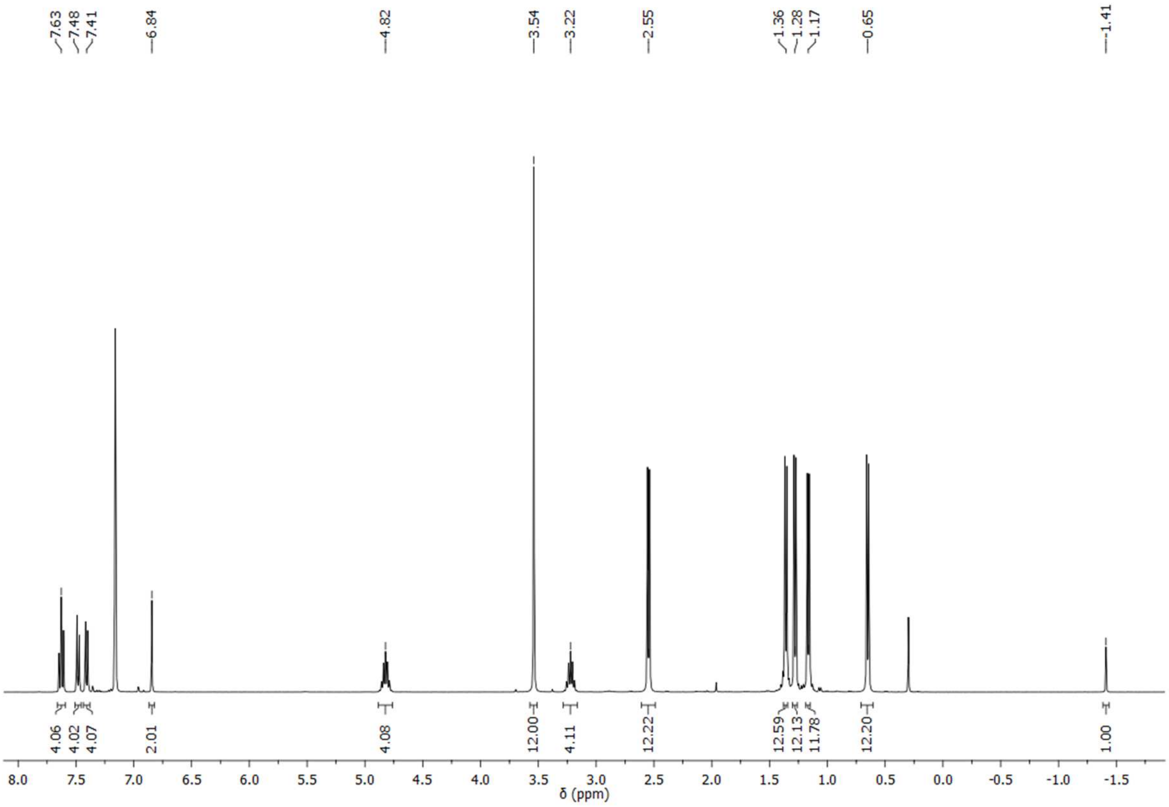

Figure S30: ^{1}H NMR spectrum of **4** in C_6D_6 at 25 °C.

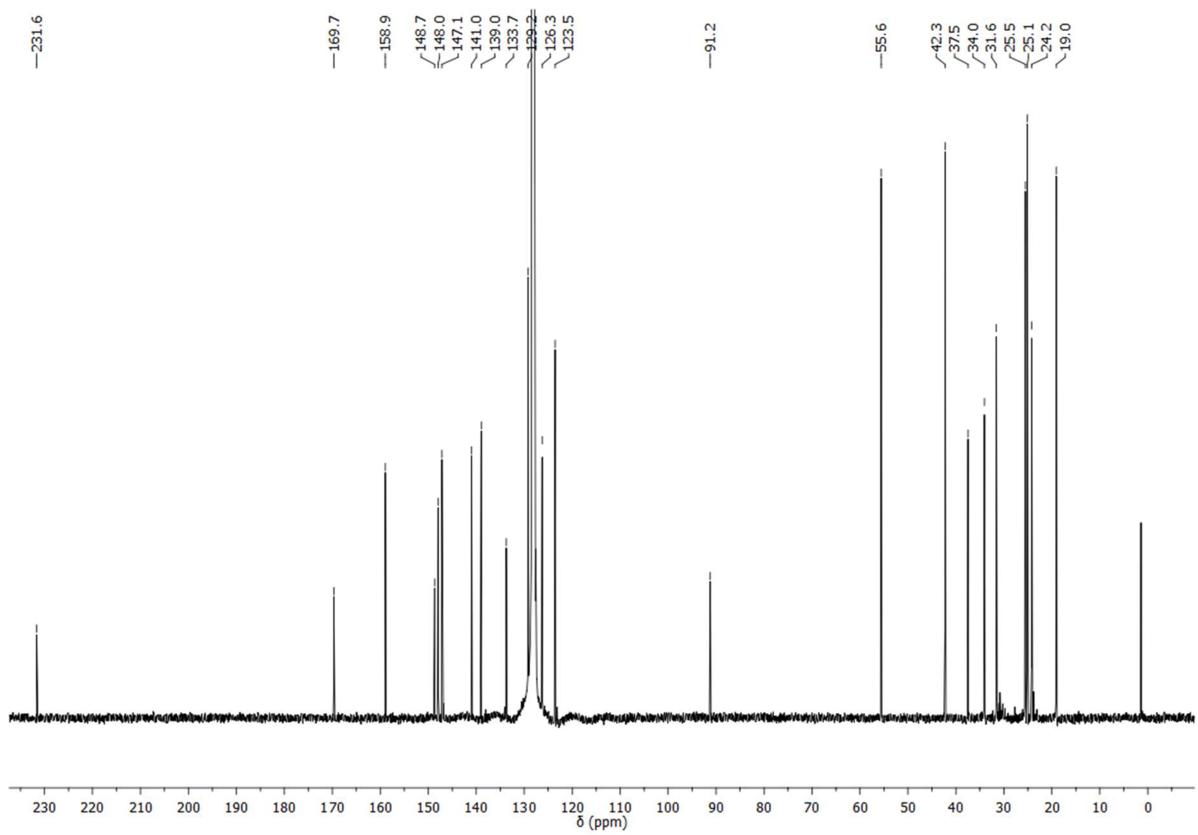

Figure S31: ^{13}C NMR spectrum of **4** in C_6D_6 at 25 °C.

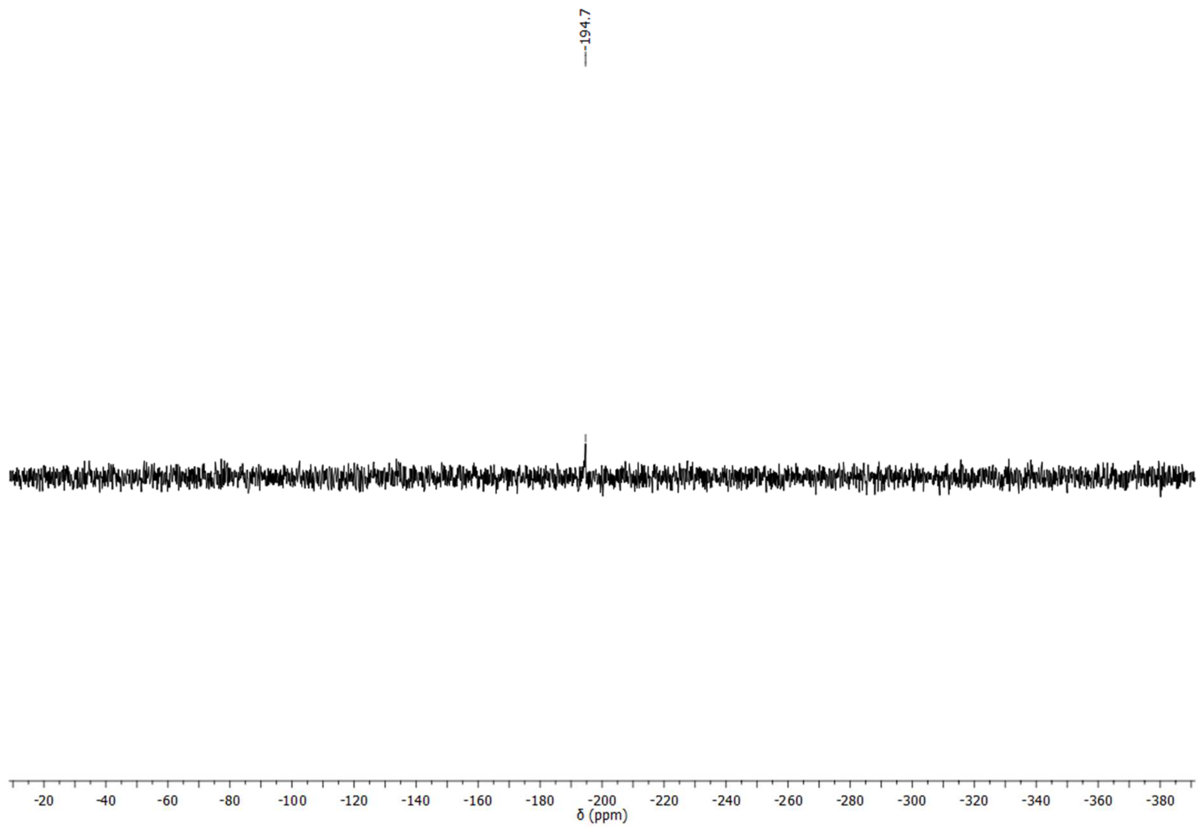

Figure S32: ^{13}C NMR spectrum (high field) of **4** in C_6D_6 at 25 °C.

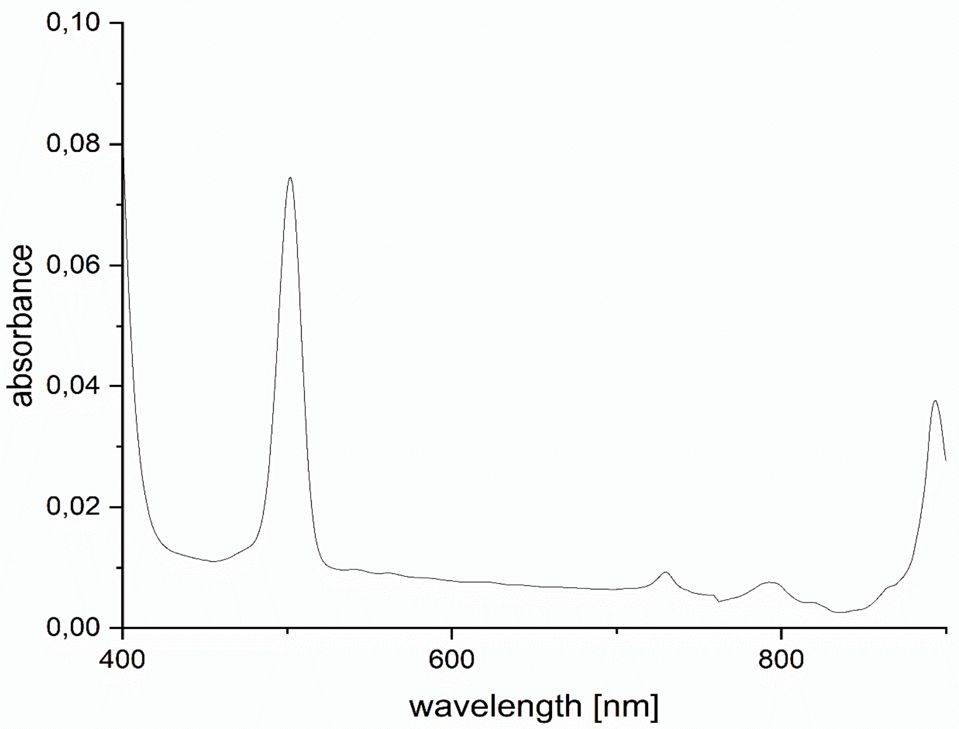

Figure S33: UV-vis spectrum of **4** in toluene (1 mmol/L) at 25 °C. The absorbance at lower wavelengths was very high due to absorption bands of the solvent and the ligand scaffold. At wavelengths higher than 900 nm, the data were noisy due to a change from a deuterium arc lamp to a tungsten filament light source.

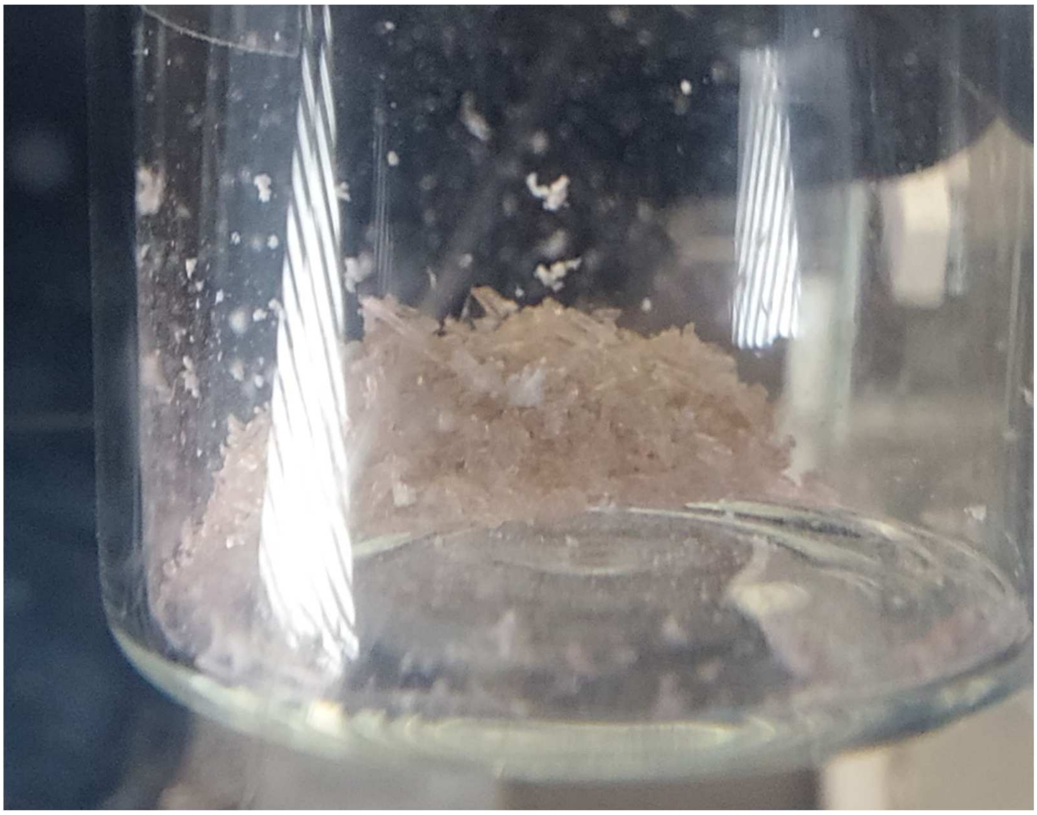

Figure S34: Picture of **4**.

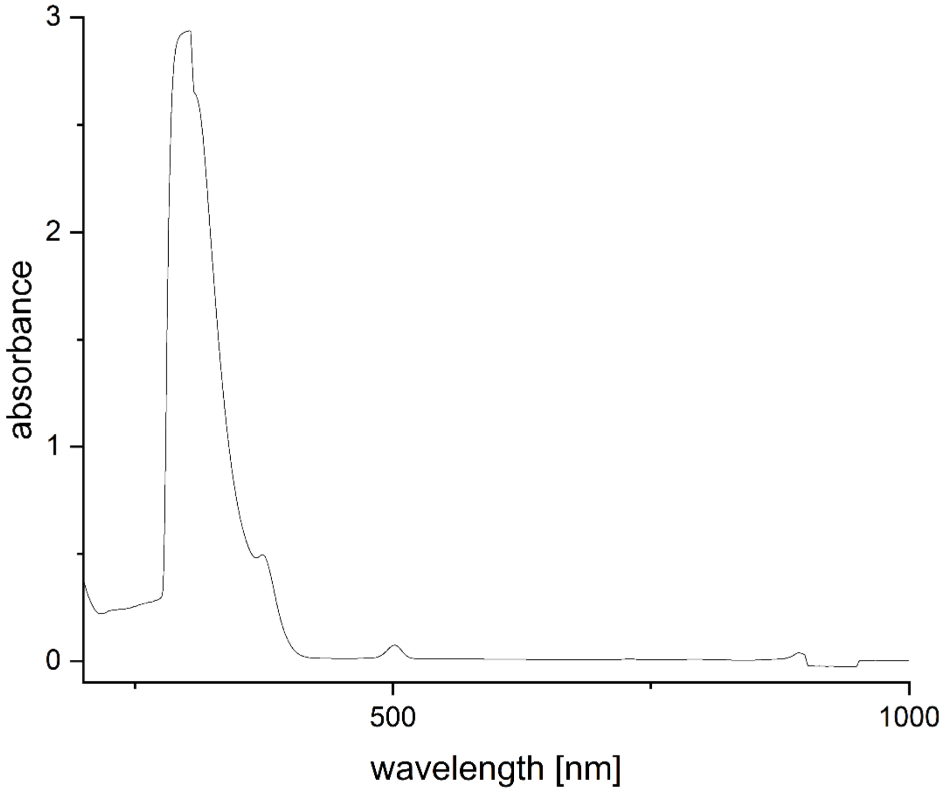

Figure S35: Complete UV-vis spectrum of **4** in toluene (1 mmol/L) at 25 °C.

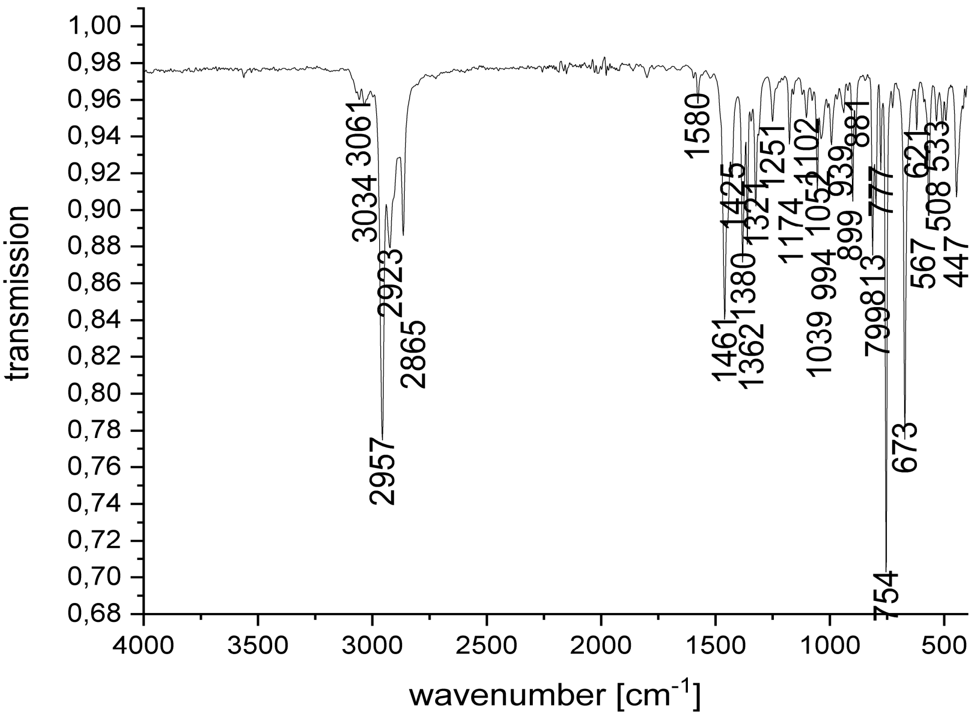

Figure S36: ATR-IR spectrum of **4**.

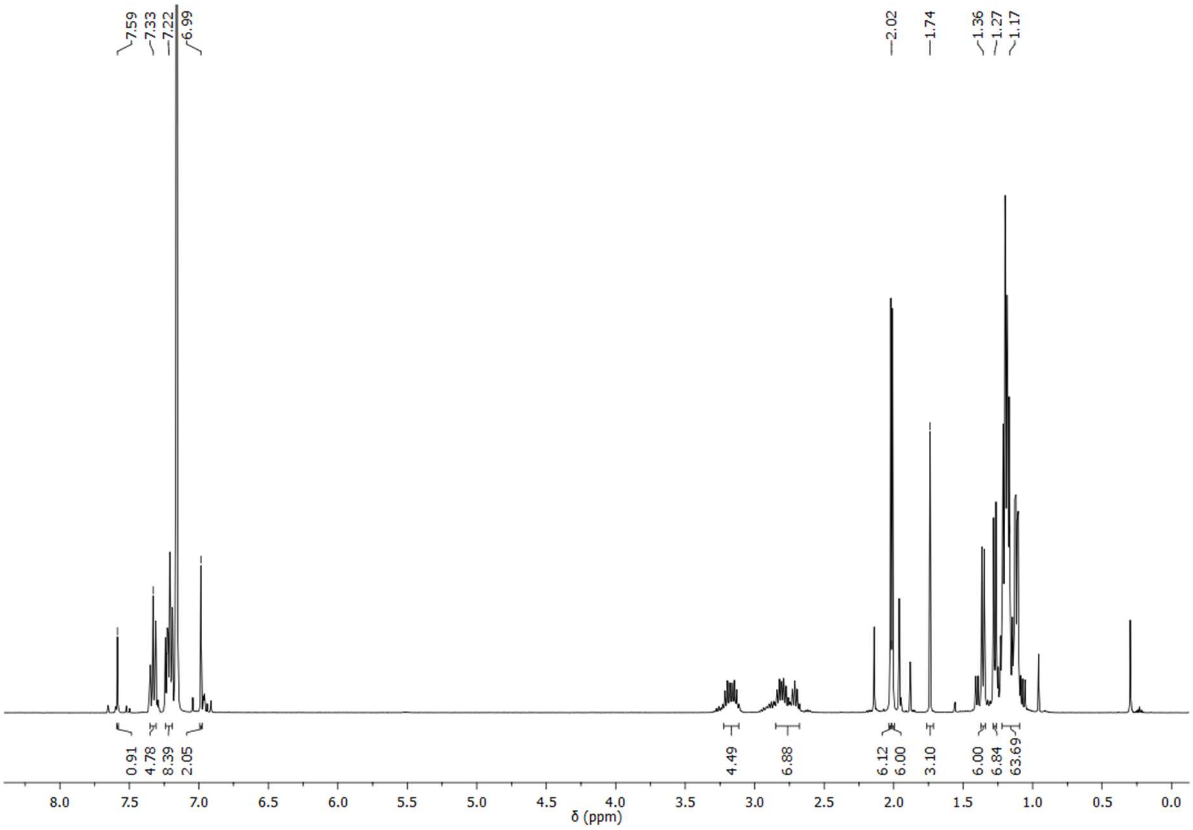

Figure S37: ^{1}H NMR spectrum of **5** in C_6D_6 at 25 °C.

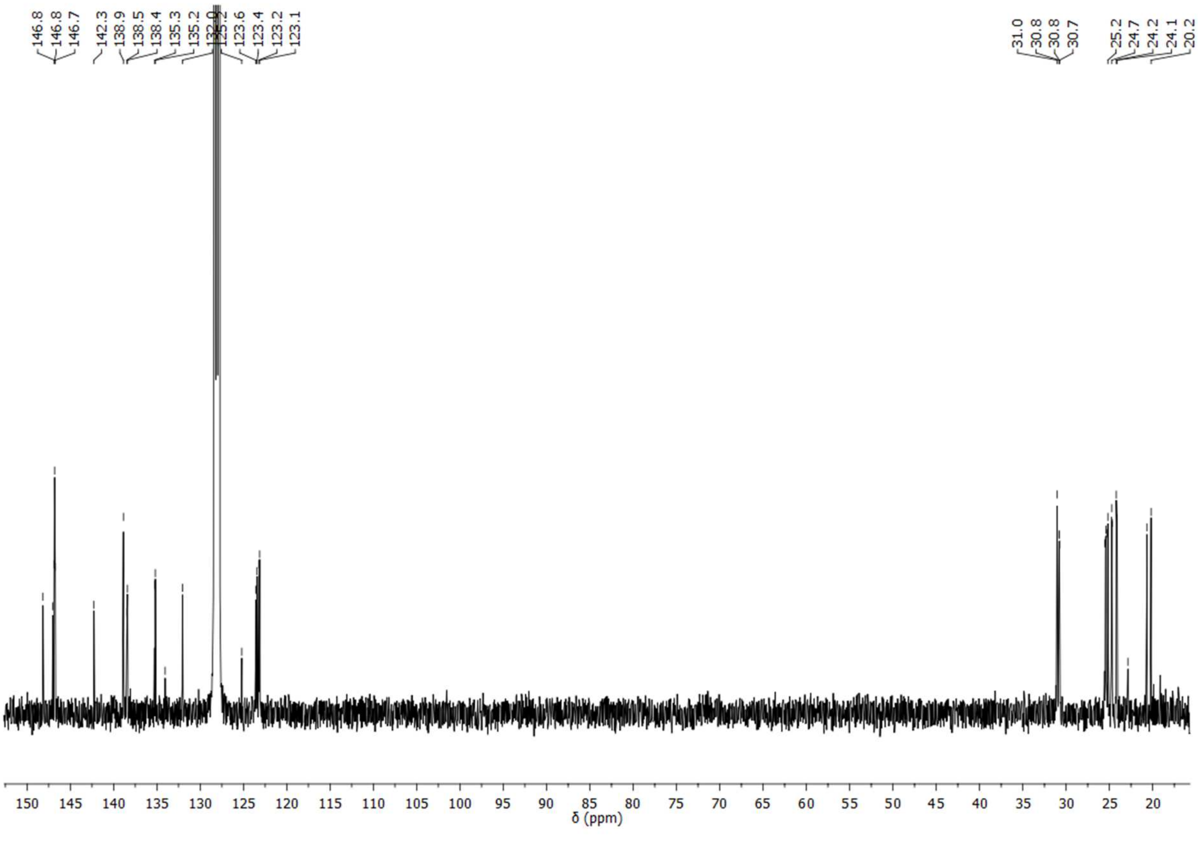

Figure S38: ^{13}C NMR spectrum of **5** in C_6D_6 at 25 °C.

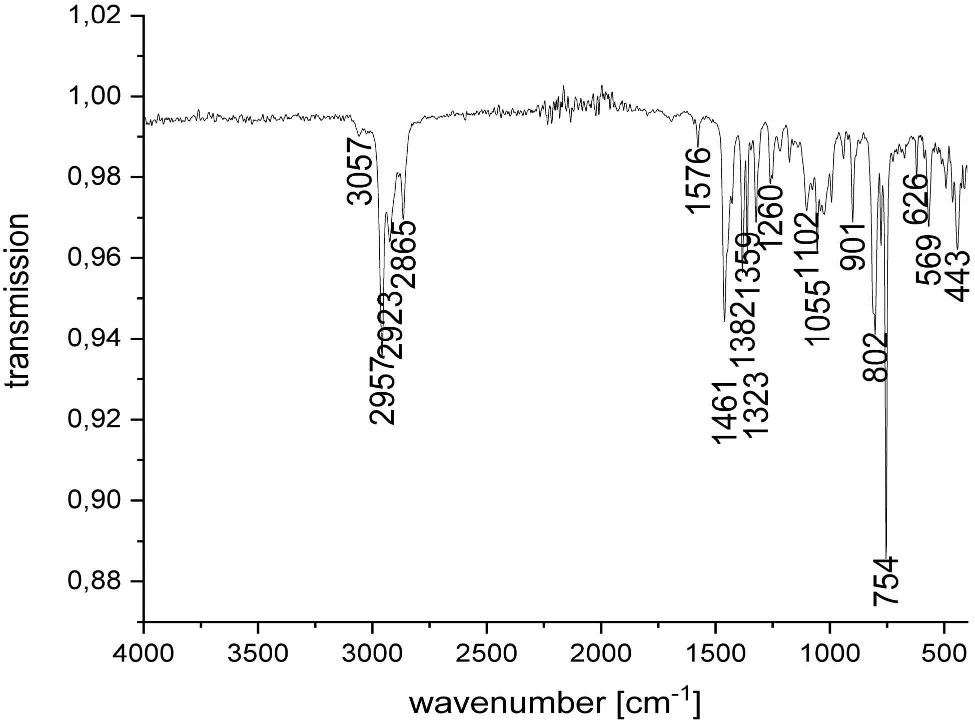

Figure S39: ATR-IR spectrum of **5**.

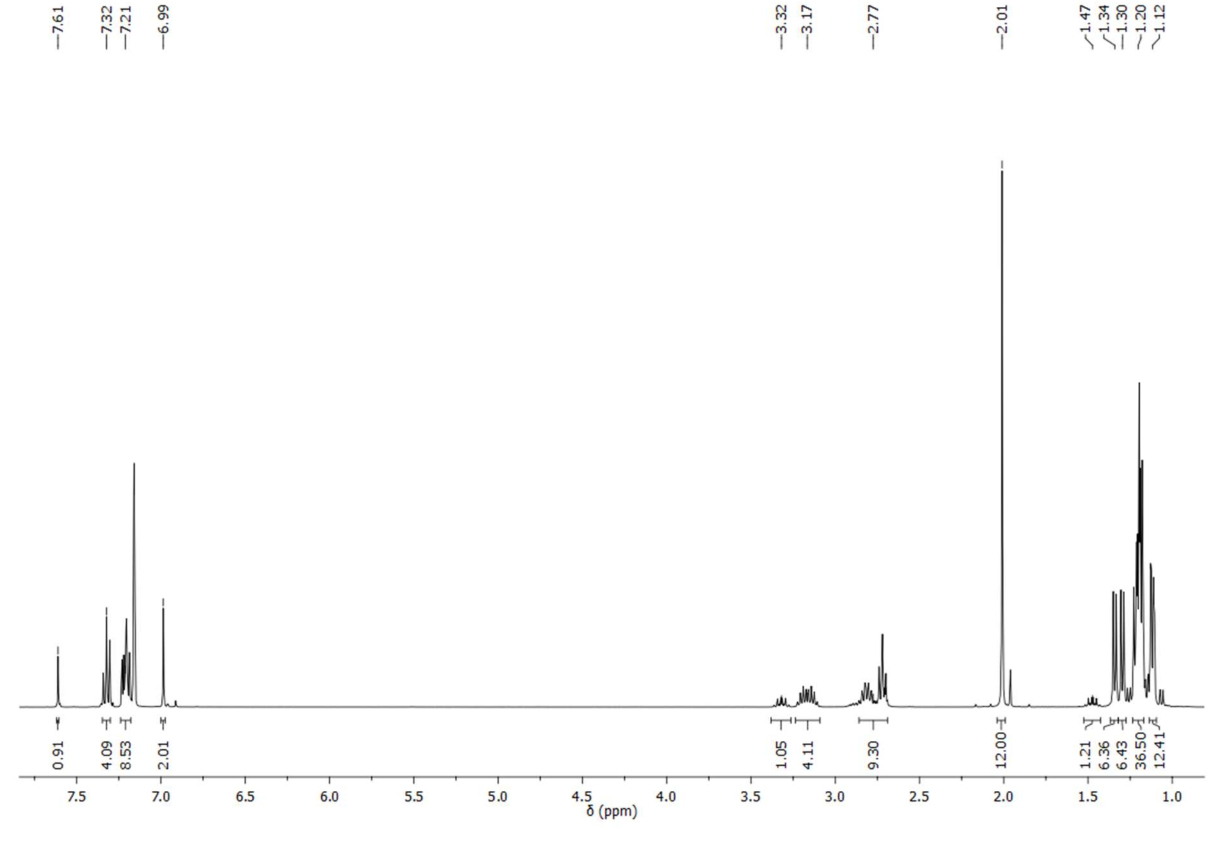

Figure S40: ^{1}H NMR spectrum of **6** in C_6D_6 at 25 °C.

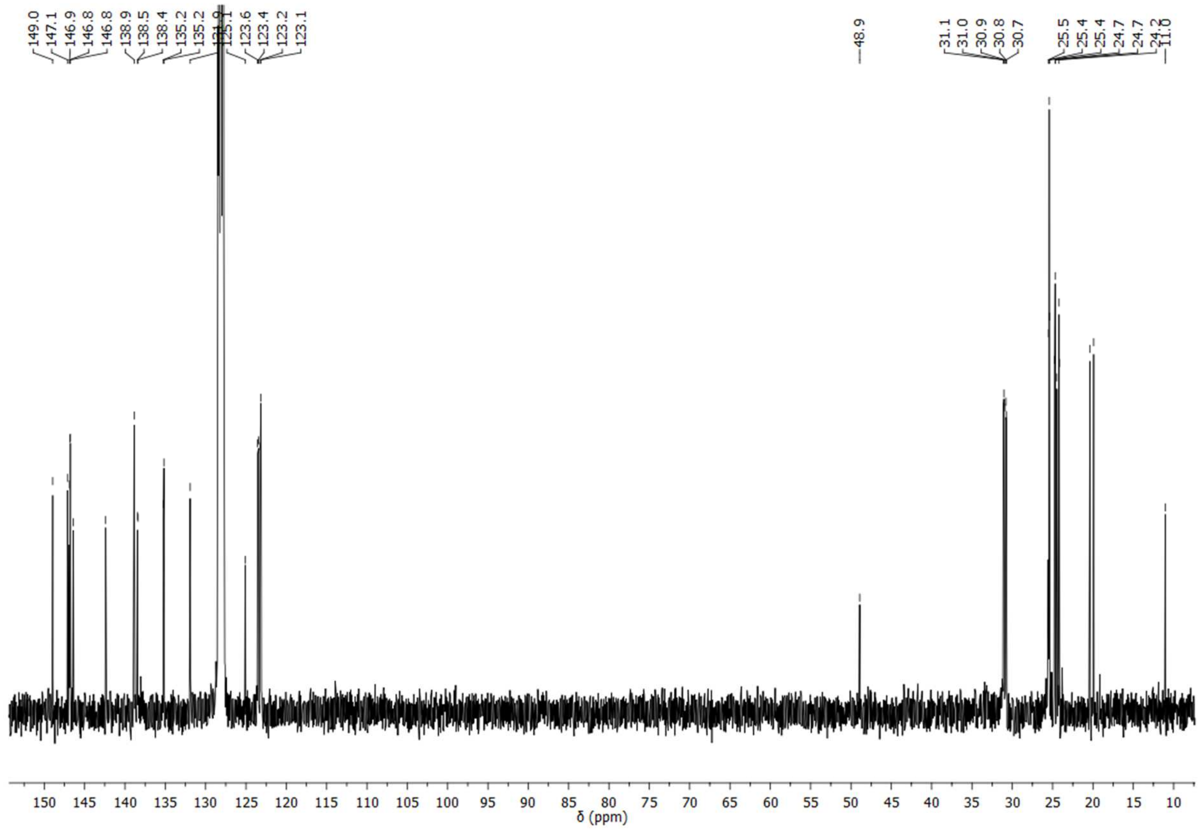

Figure S41: ^{13}C NMR spectrum of **6** in C_6D_6 at 25 °C.

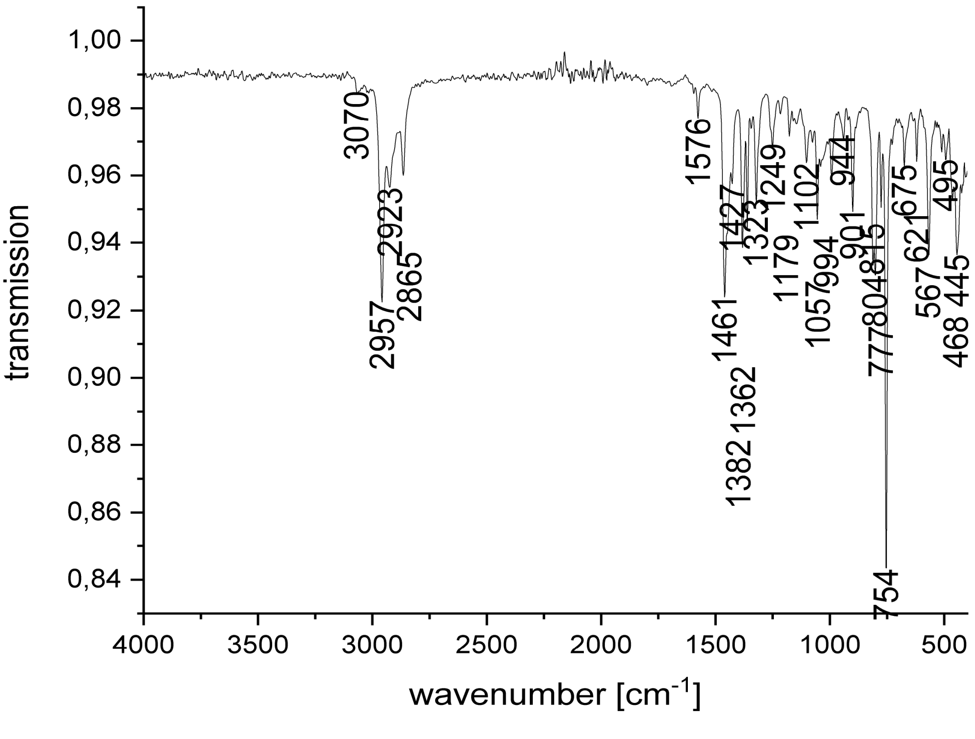

Figure S42: ATR-IR spectrum of **6**.

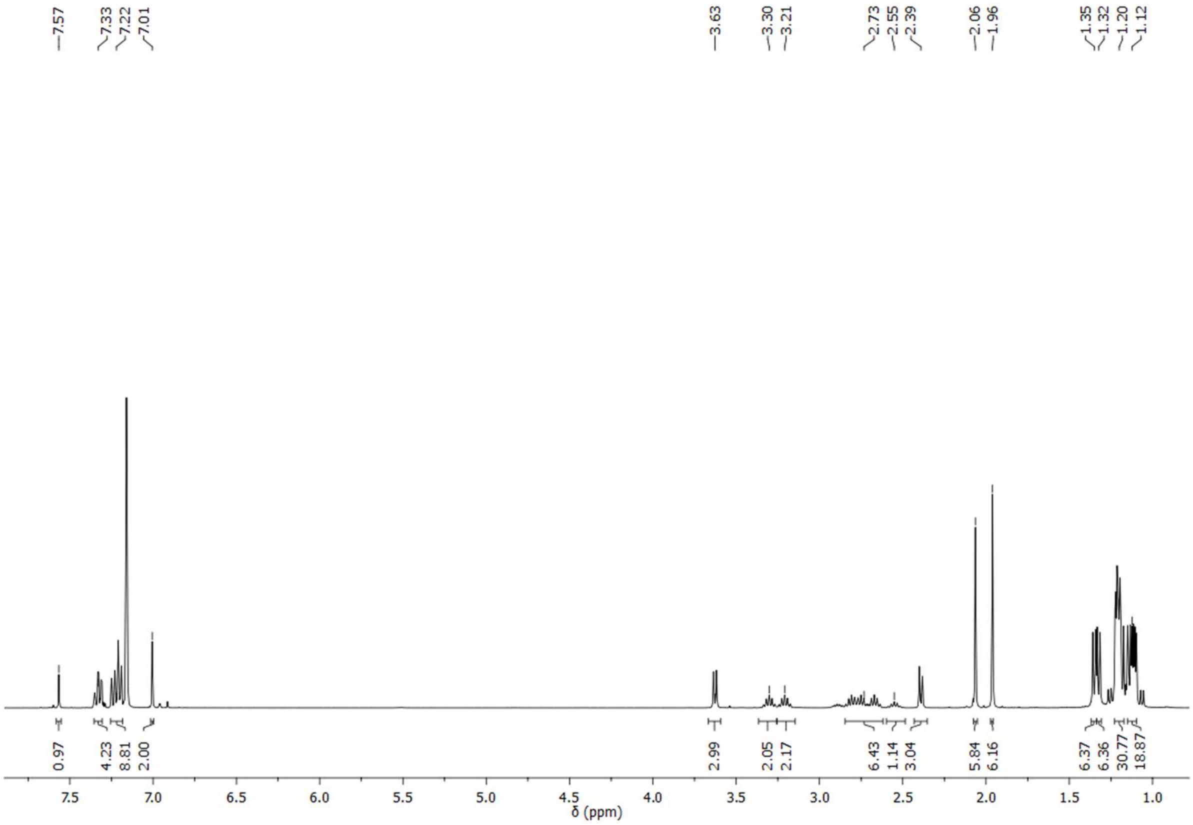

Figure S43: ^{1}H NMR spectrum of **7** in C_6D_6 at 25 °C.

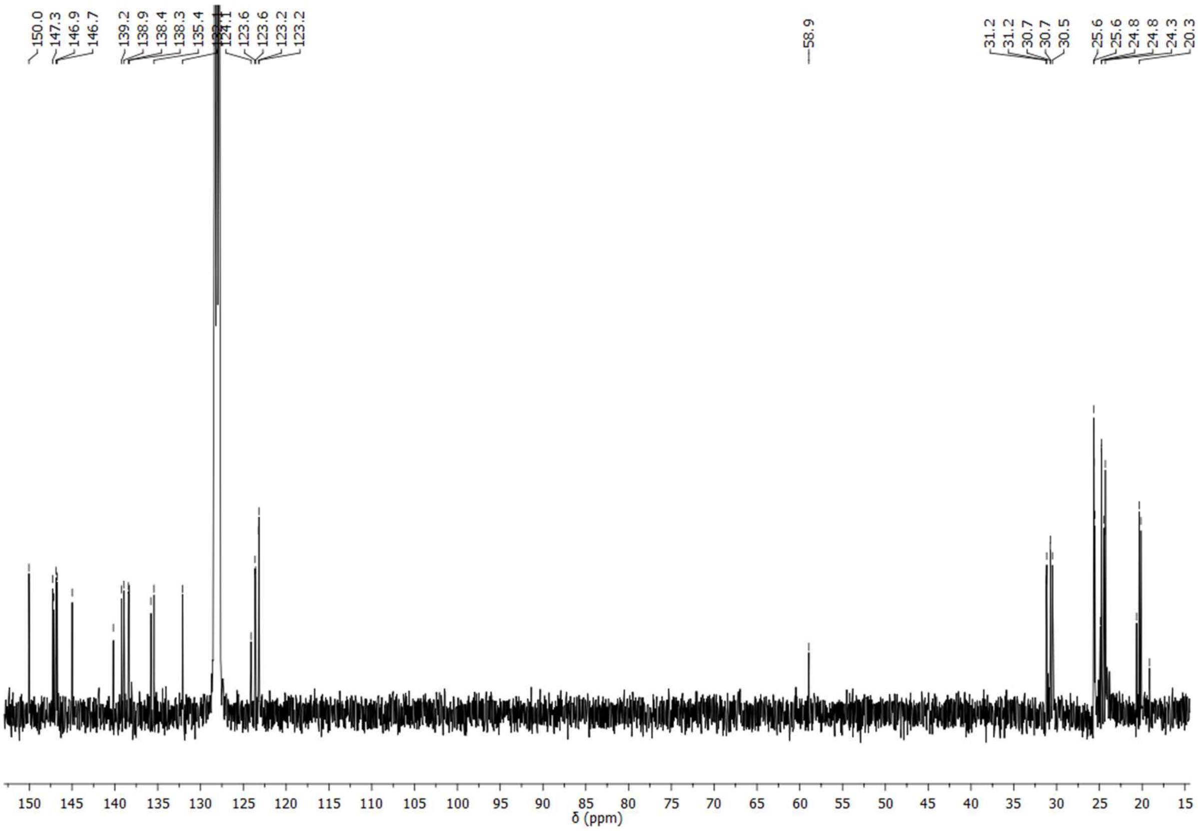

Figure S44: ^{13}C NMR spectrum of **7** in C_6D_6 at 25 °C.

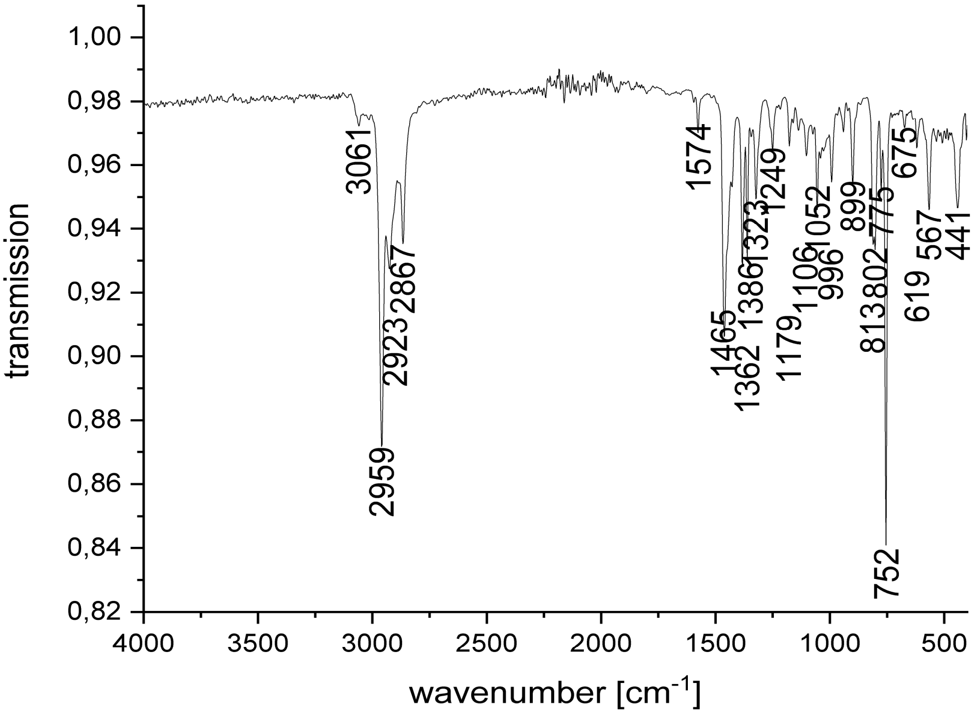

Figure S45: ATR-IR spectrum of **7**.

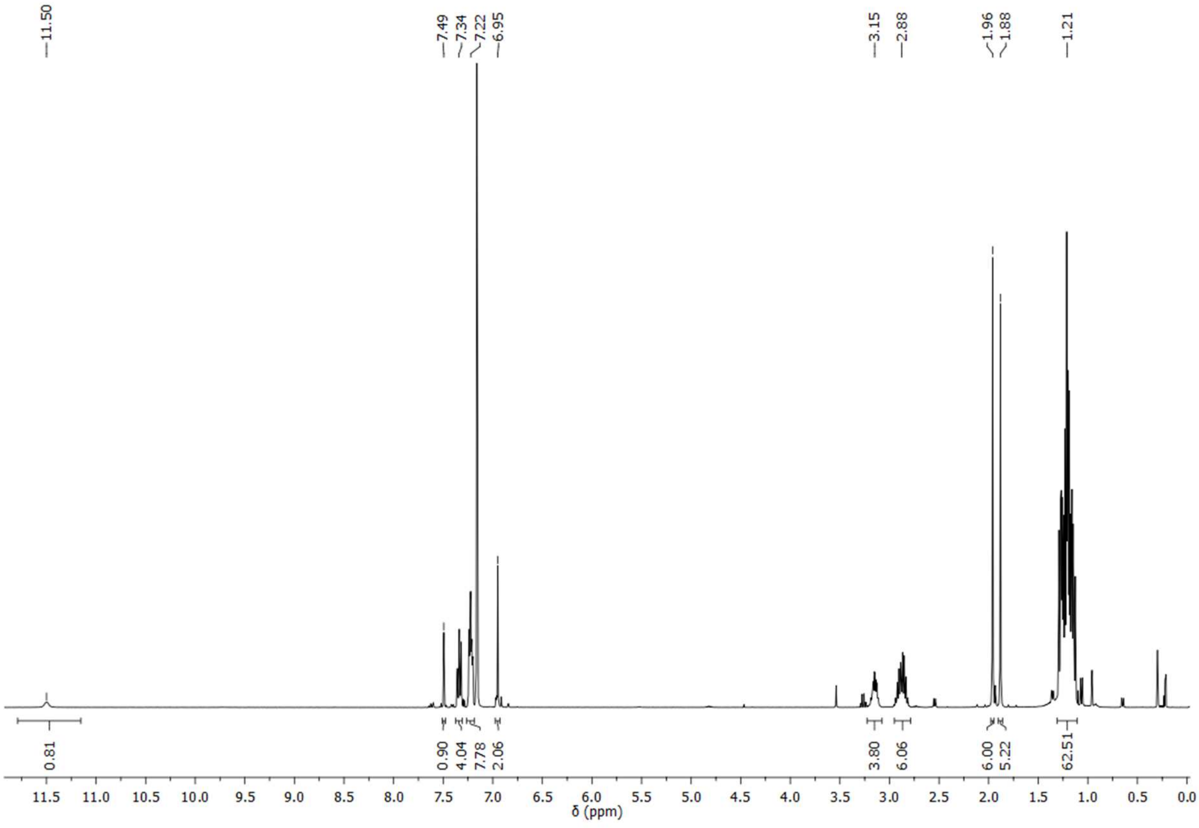

Figure S46: ^{1}H NMR spectrum of **8** in C_6D_6 at 25 °C.

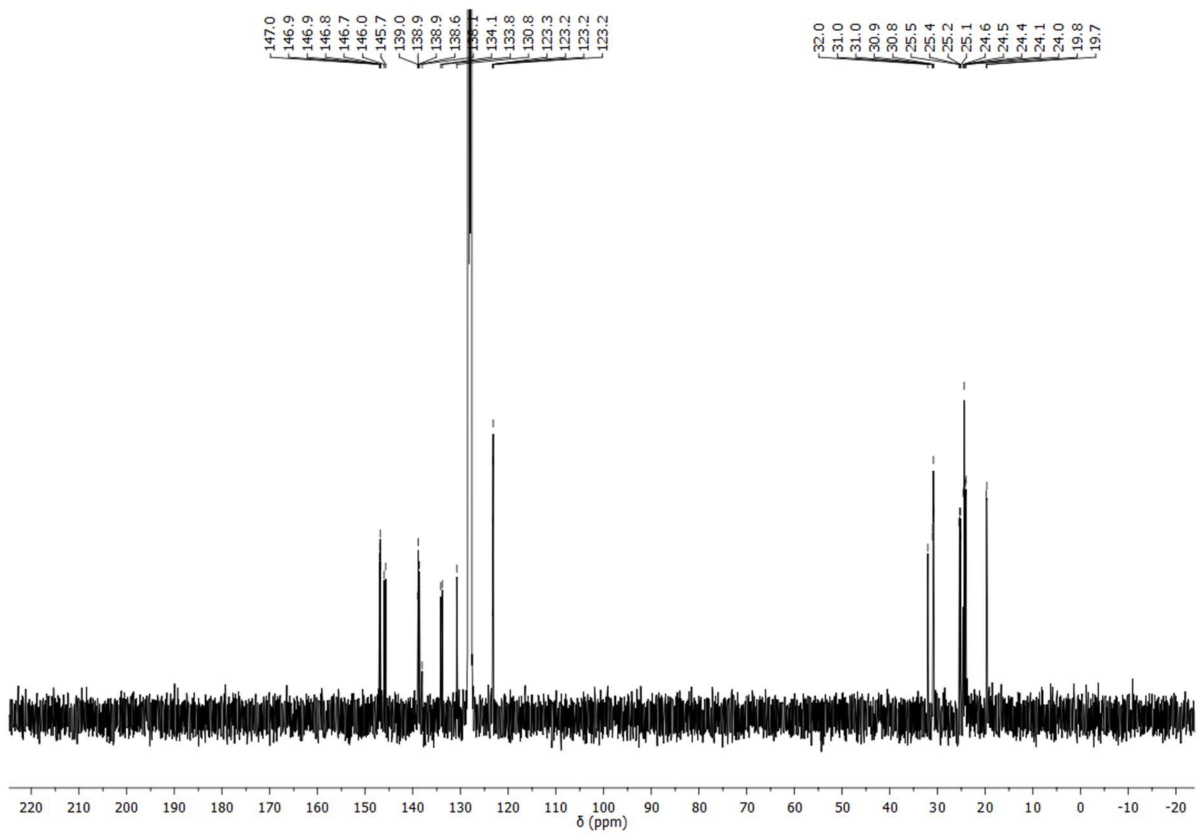

Figure S47: ^{13}C NMR spectrum of **8** in C_6D_6 at 25 °C.

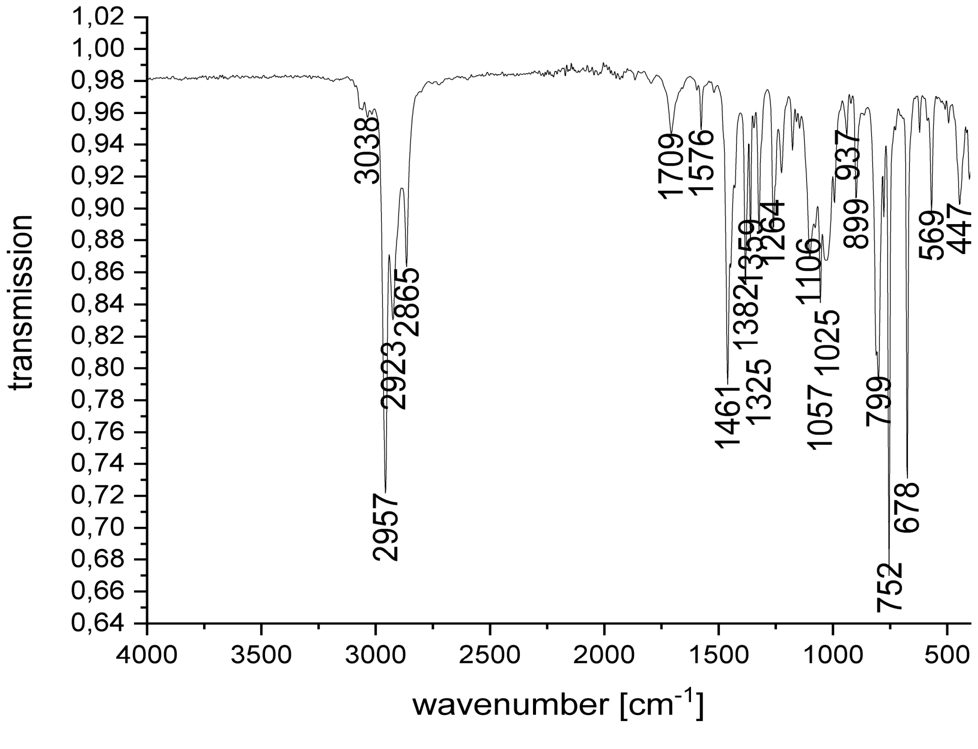

Figure S48: ATR-IR spectrum of **8**.

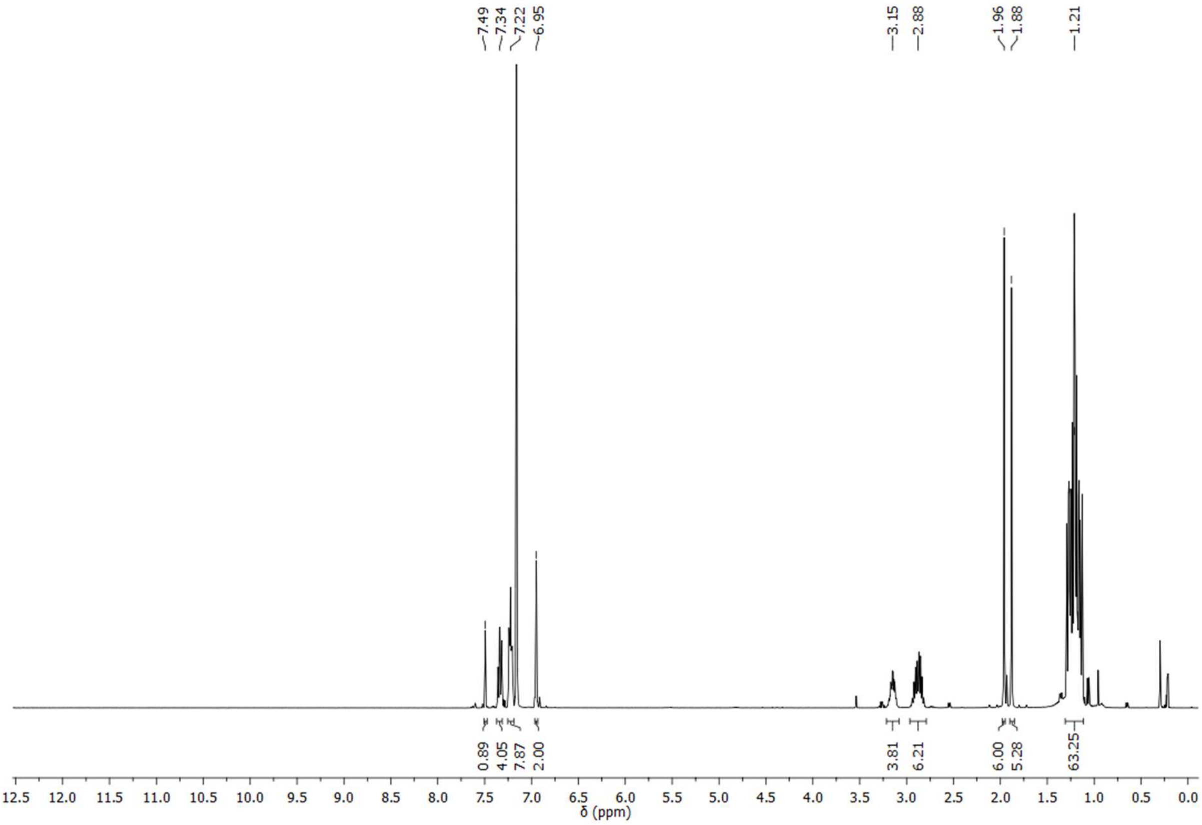

Figure S49: ^{1}H NMR spectrum of **9** in C_6D_6 at 25 °C.

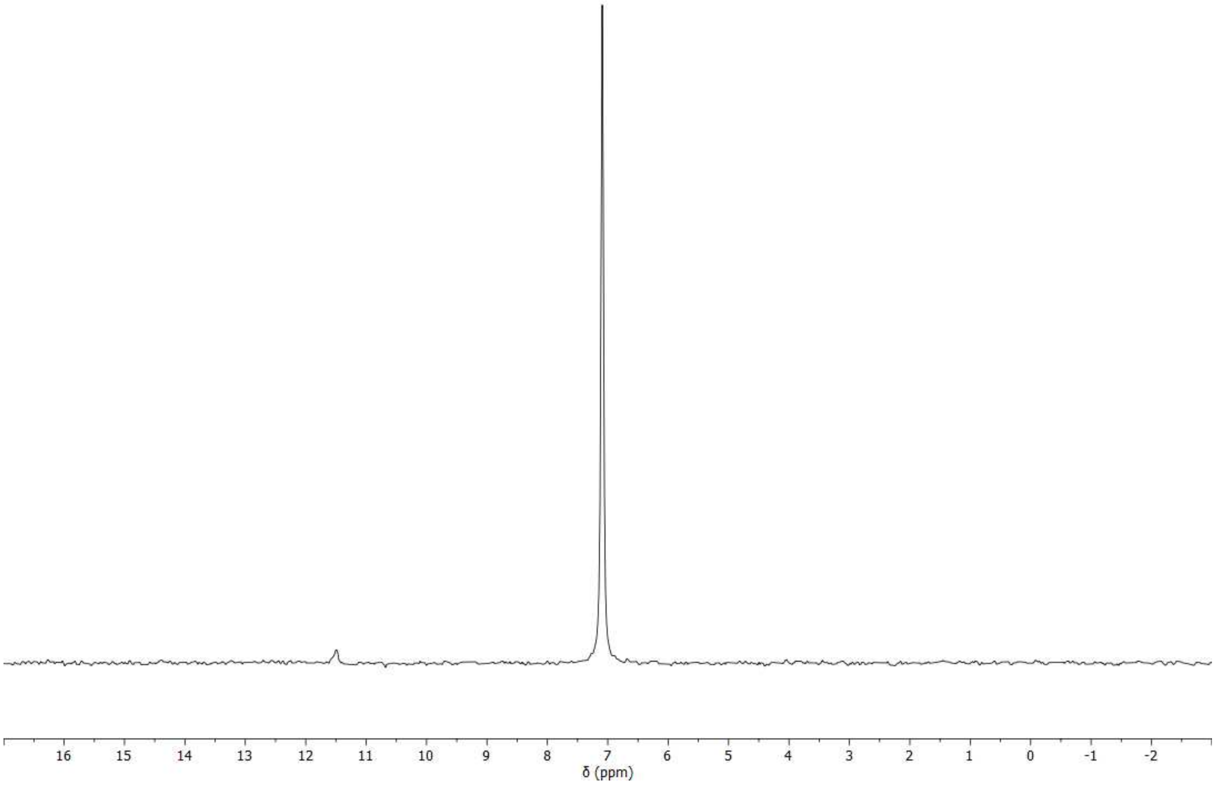

Figure S50: ^{2}H NMR spectrum of **9** in C_6H_6 at 25 °C.

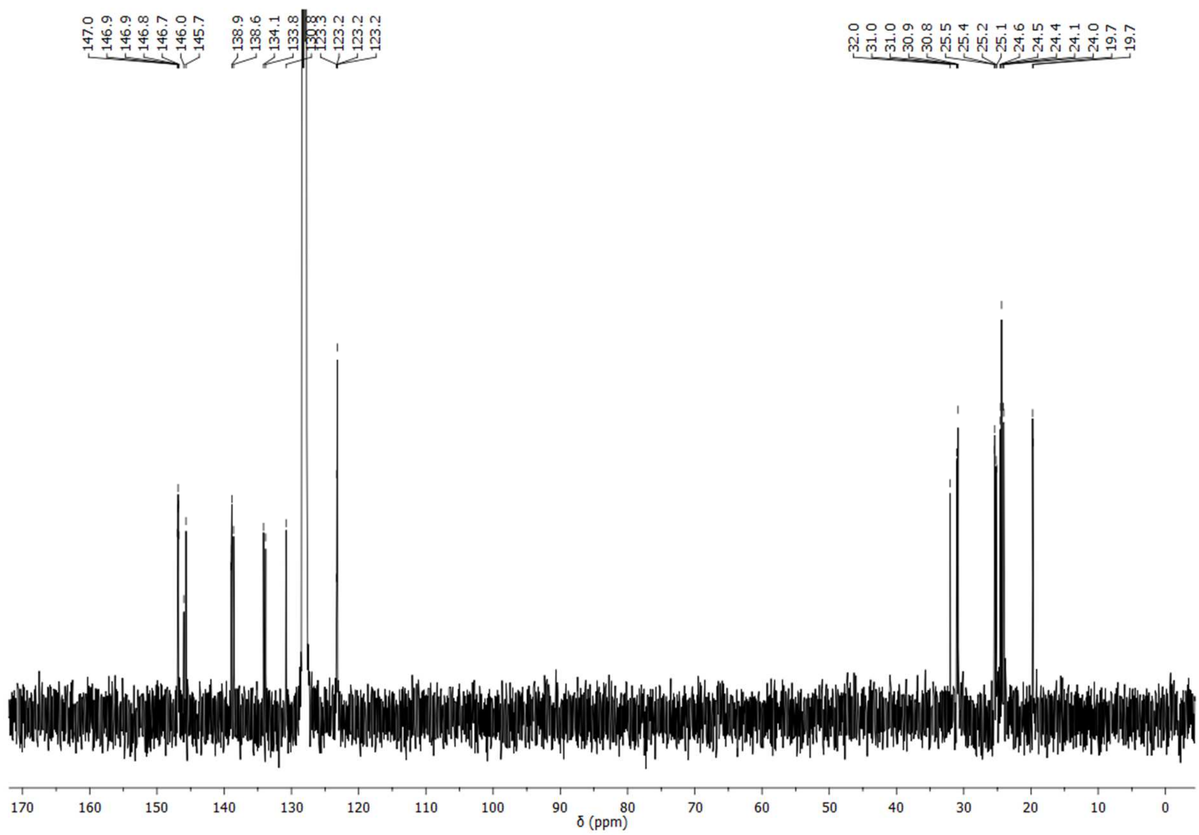

Figure S51: ^{13}C NMR spectrum of **9** in C_6D_6 at 25 °C.

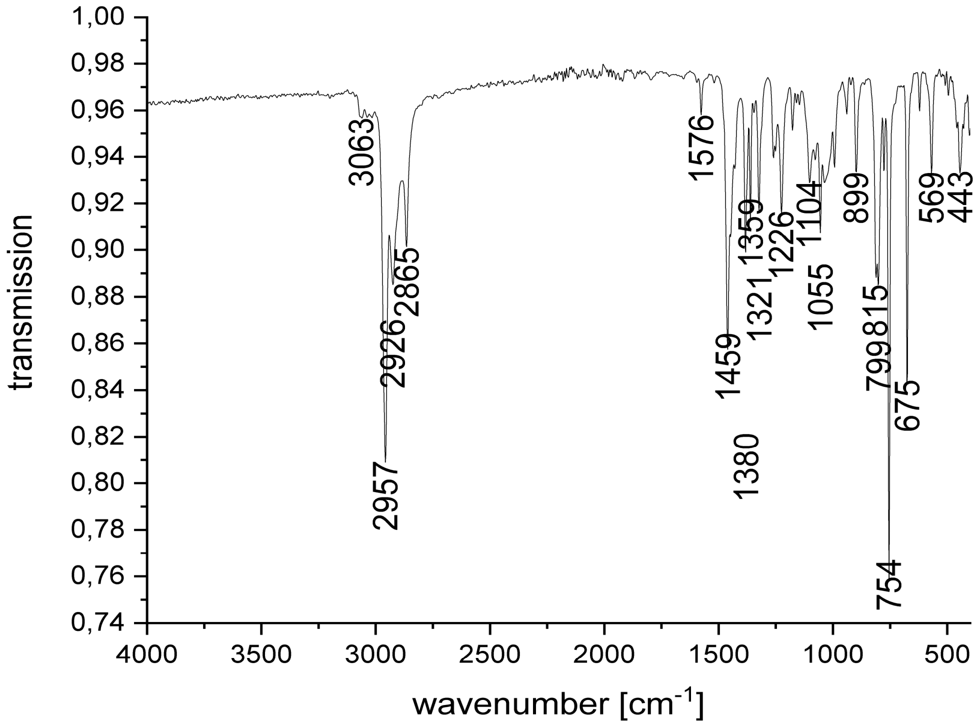

Figure S52: ATR-IR spectrum of **9**.

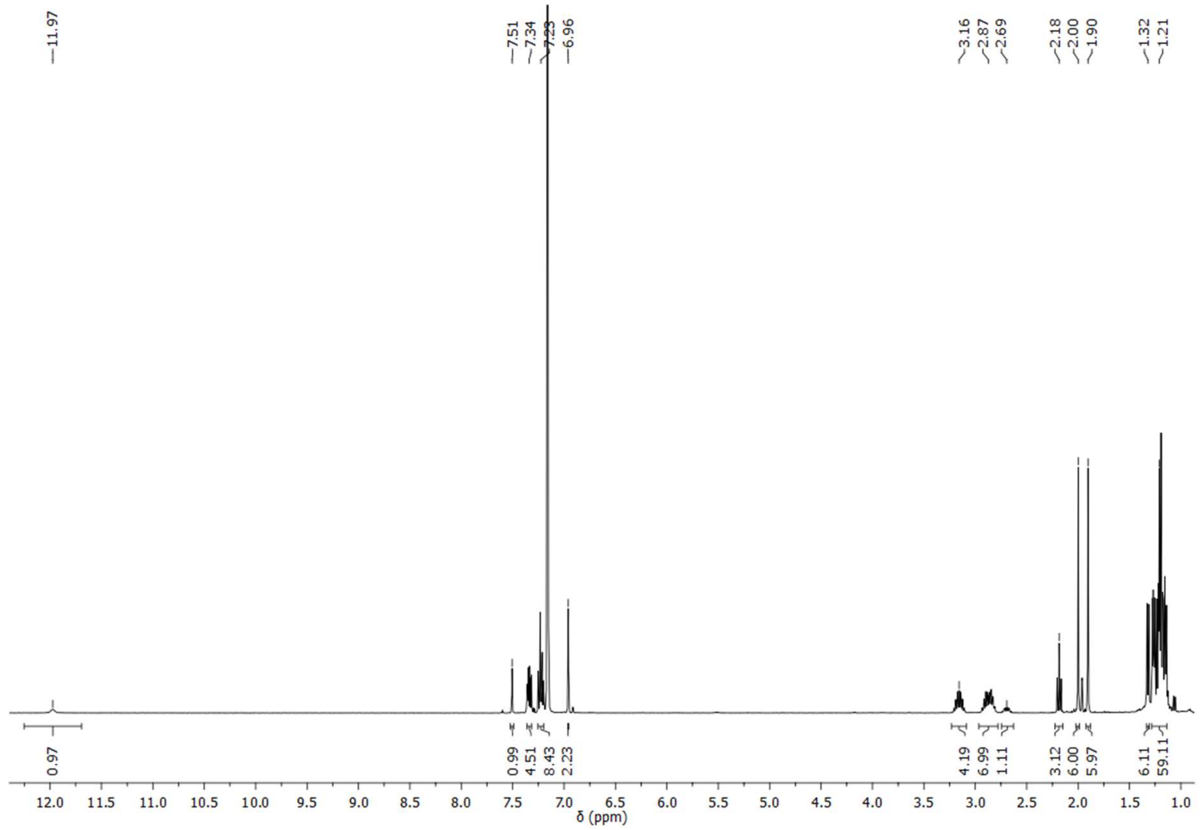

Figure S53: ^{1}H NMR spectrum of **10** in C_6D_6 at 25 °C.

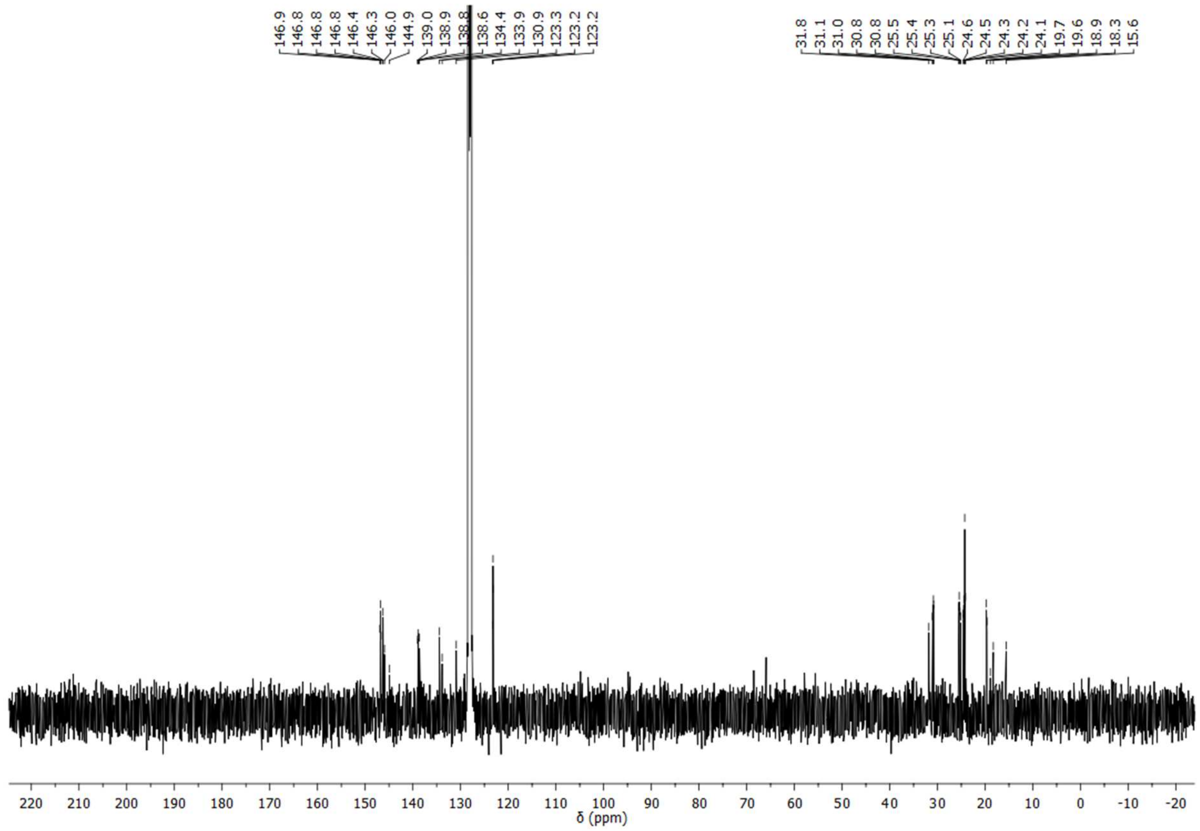

Figure S54: ^{13}C NMR spectrum of **10** in C_6D_6 at 25 °C.

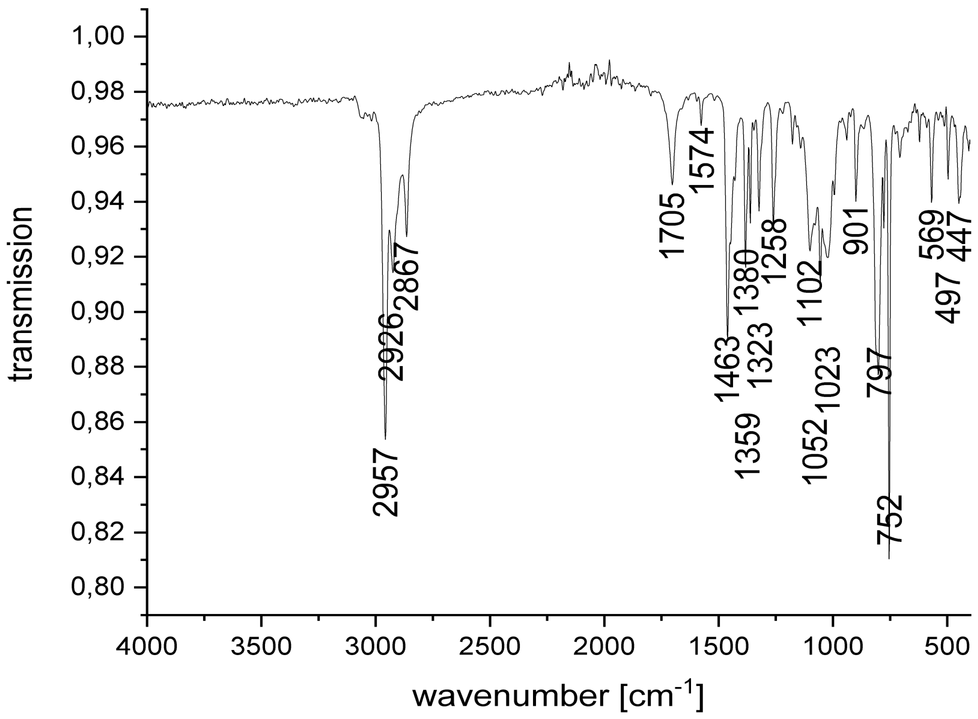

Figure S55: ATR-IR spectrum of **10**.

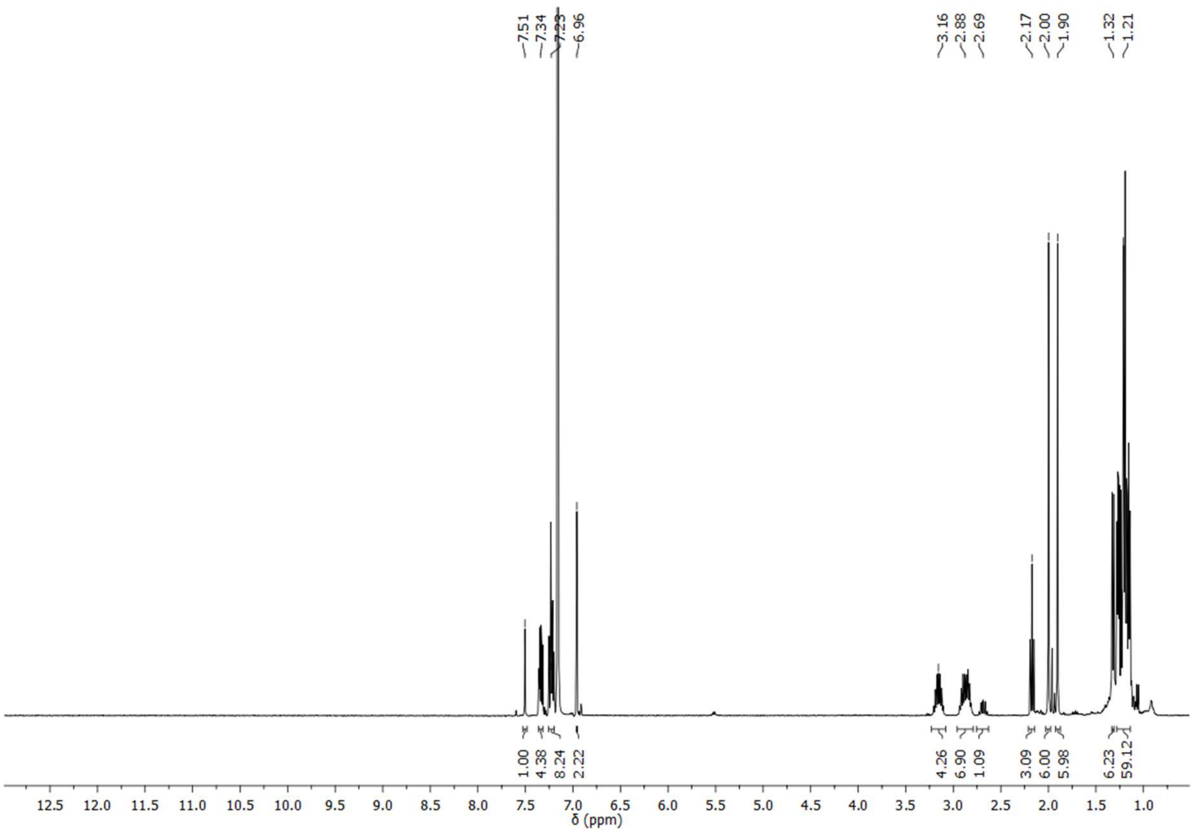

Figure S56: ^{1}H NMR spectrum of **11** in C_6D_6 at 25 °C.

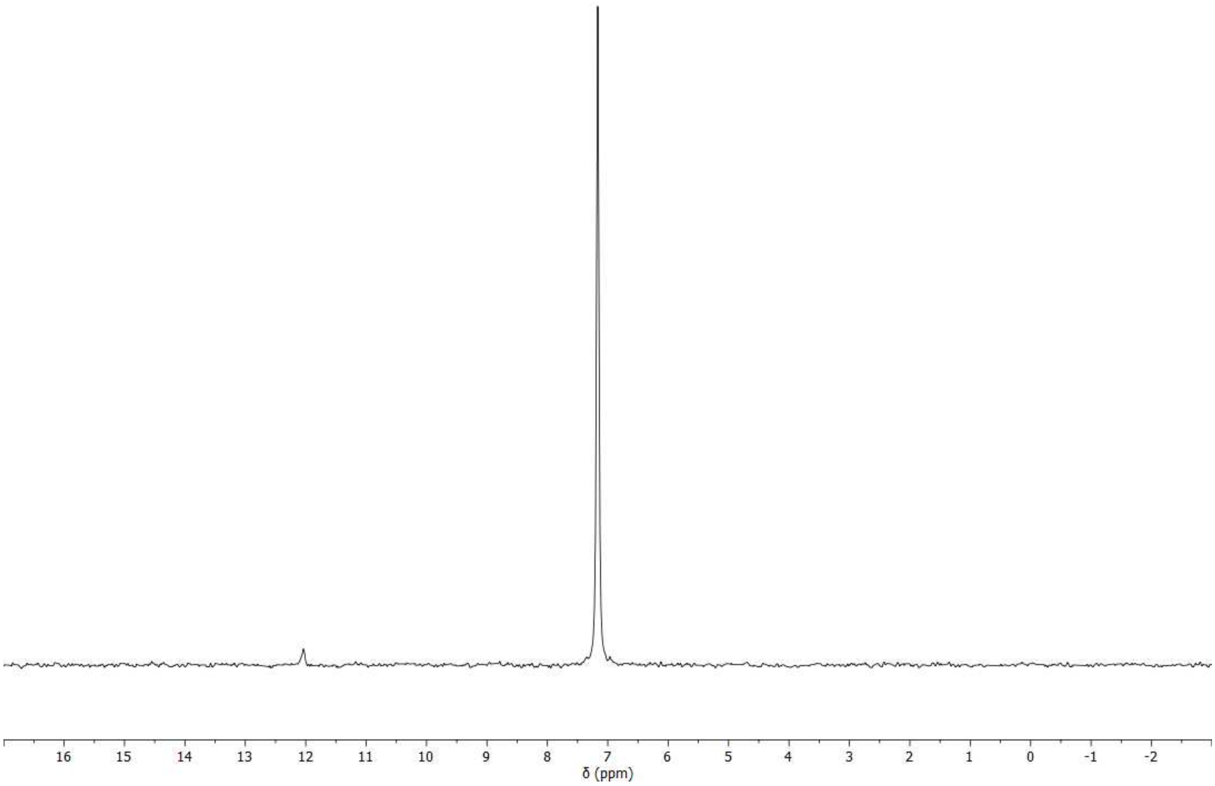

Figure S57: ^{2}H NMR spectrum of **11** in C_6H_6 at 25 °C.

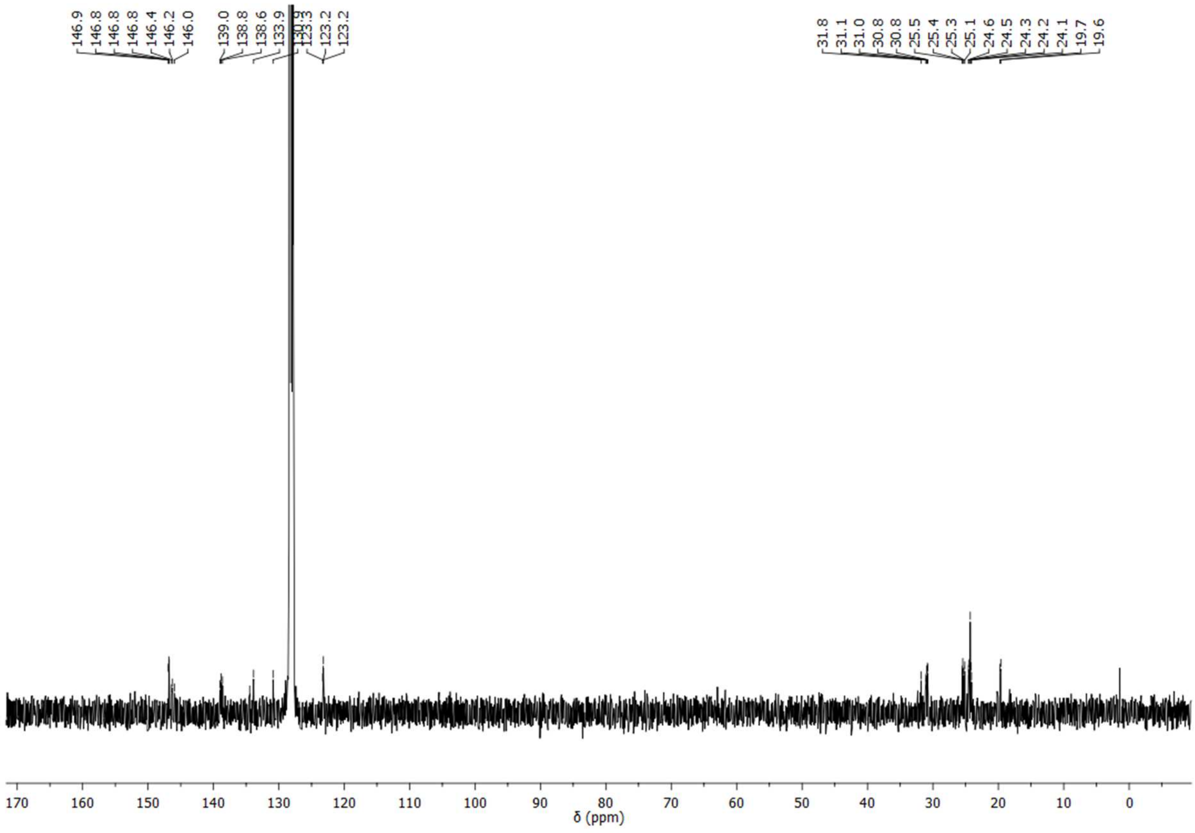

Figure S58: ^{13}C NMR spectrum of **11** in C_6D_6 at 25 °C.

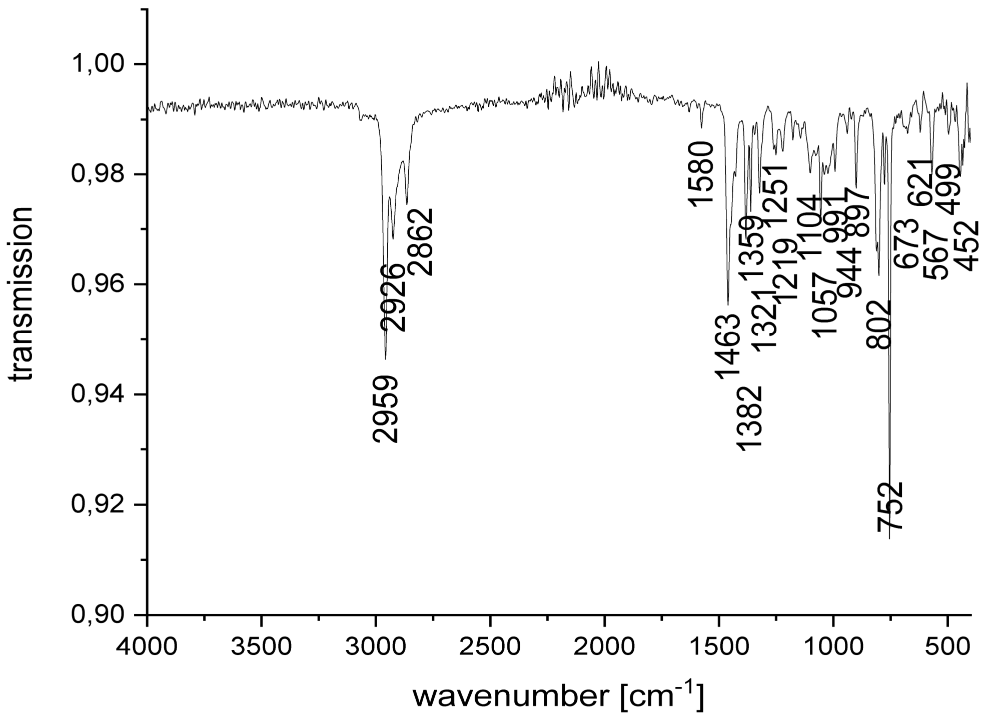

Figure S59: ATR-IR spectrum of **11**.

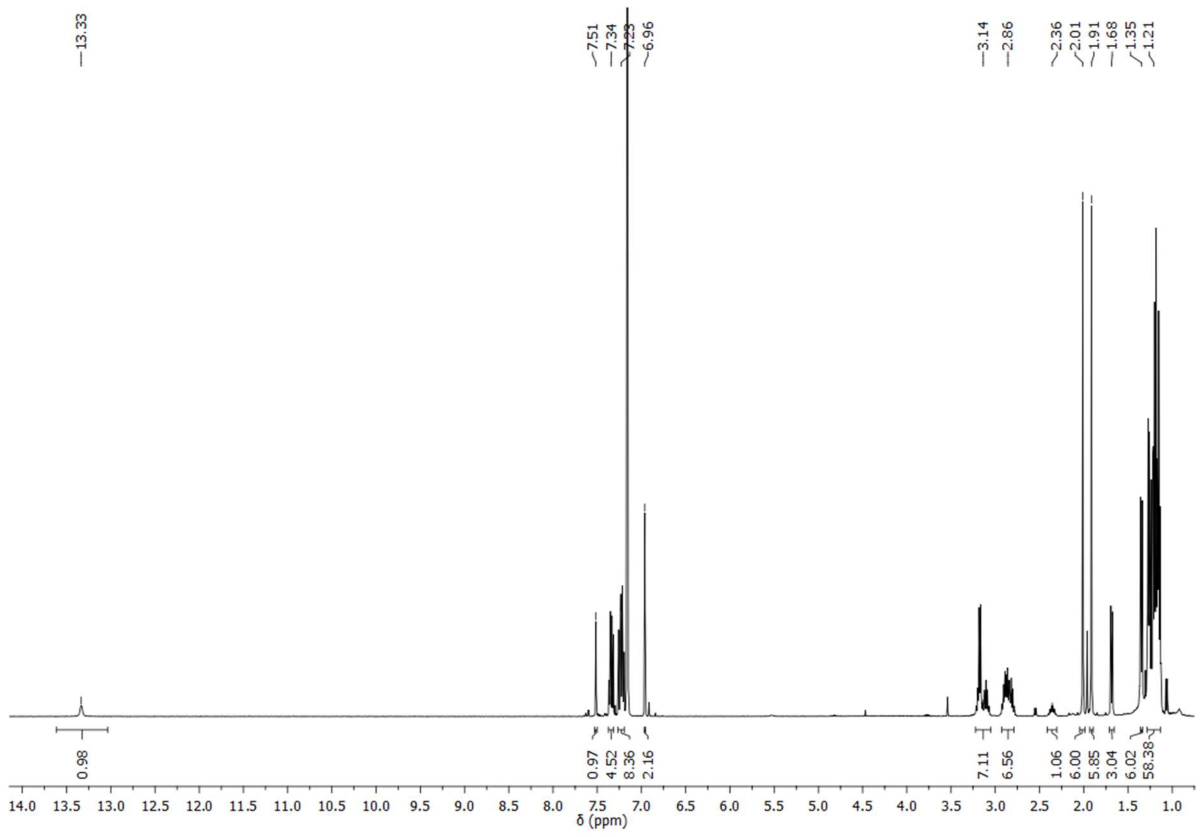

Figure S60: ^{1}H NMR spectrum of **12** in C_6D_6 at 25 °C.

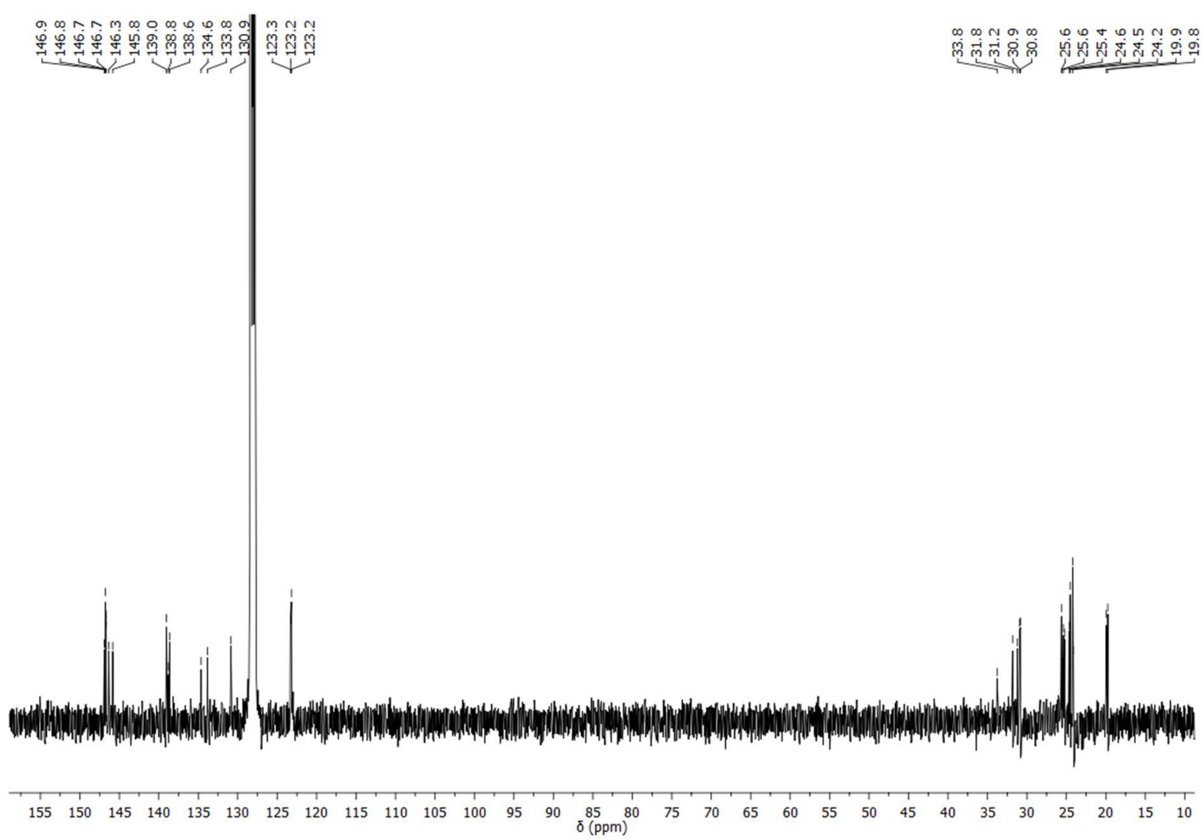

Figure S61: ^{13}C NMR spectrum of **12** in C_6D_6 at 25 °C.

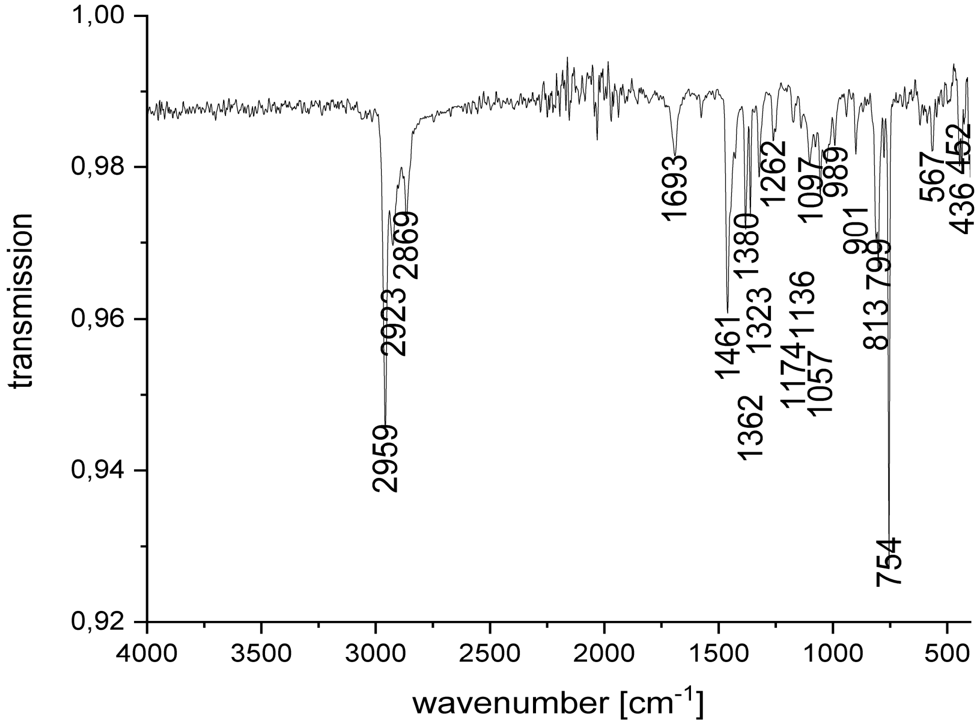

Figure S62: ATR-IR spectrum of **12**.

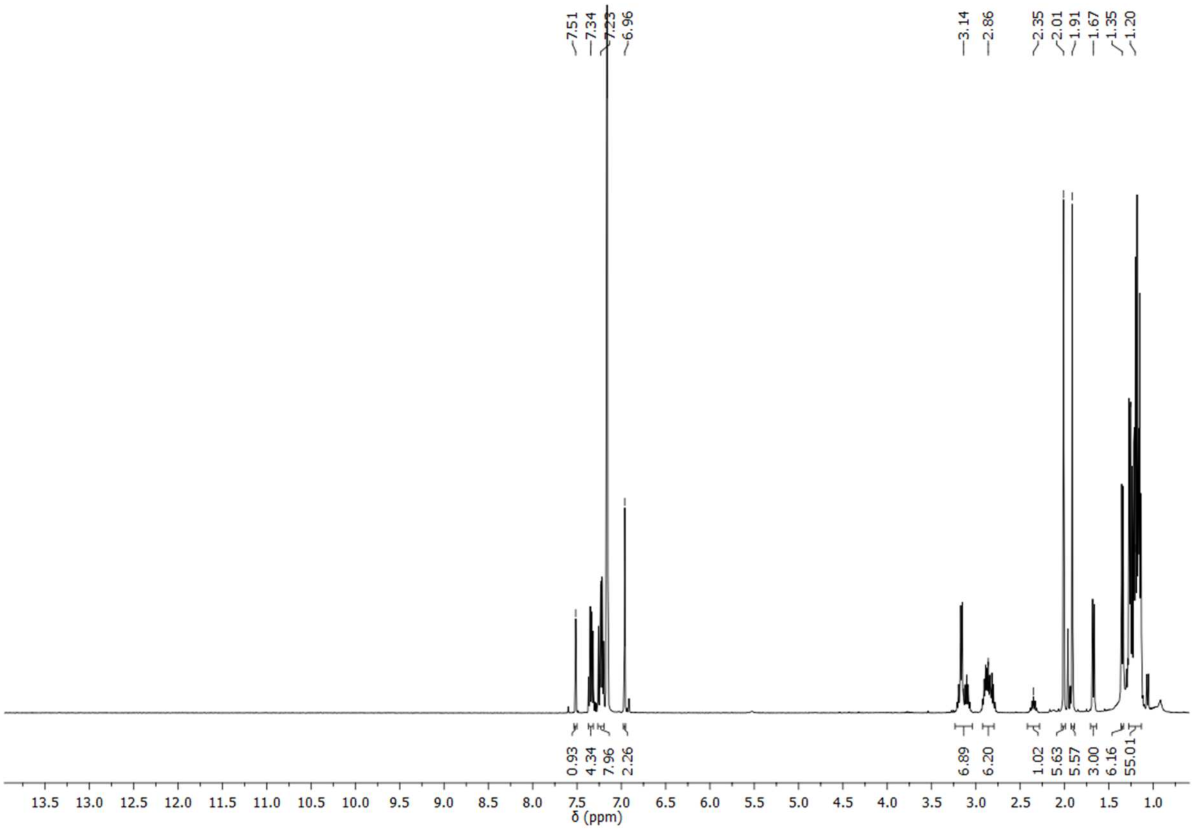

Figure S63: ^{1}H NMR spectrum of **13** in C_6D_6 at 25 °C.

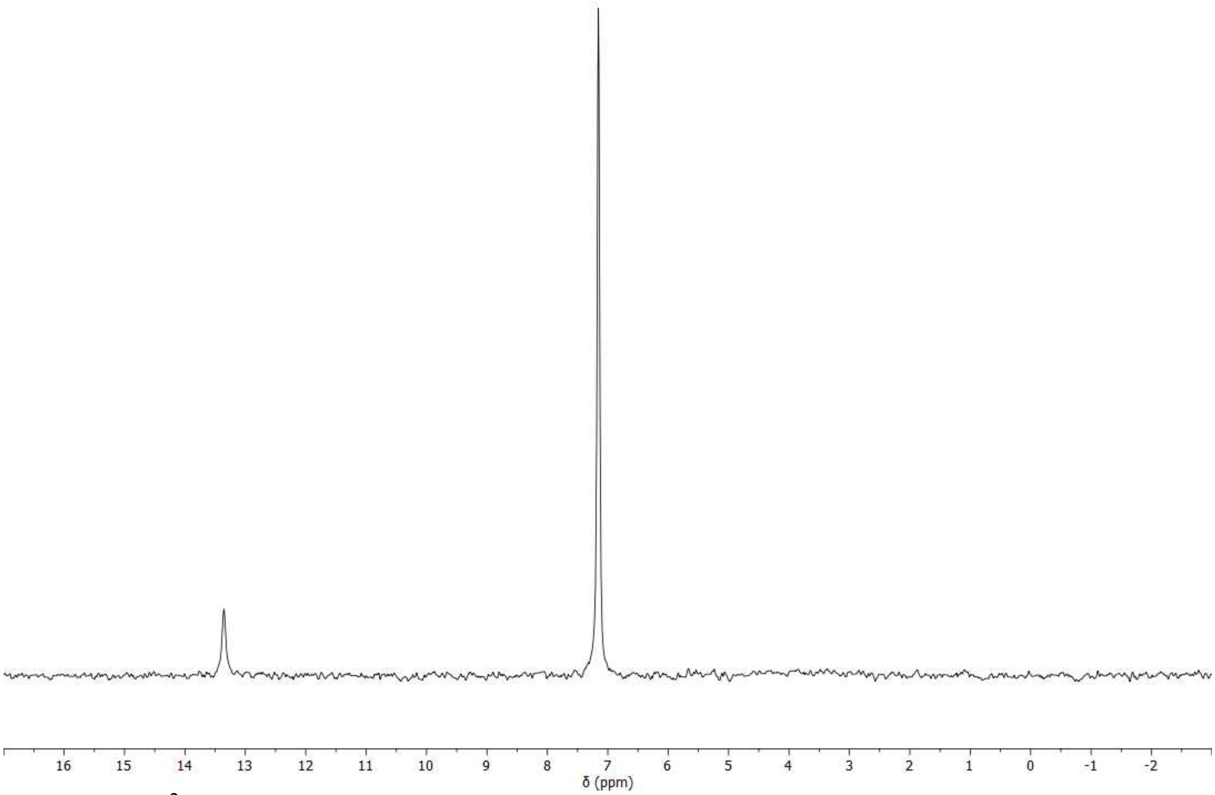

Figure S64: ^{2}H NMR spectrum of **13** in C_6H_6 at 25 °C.

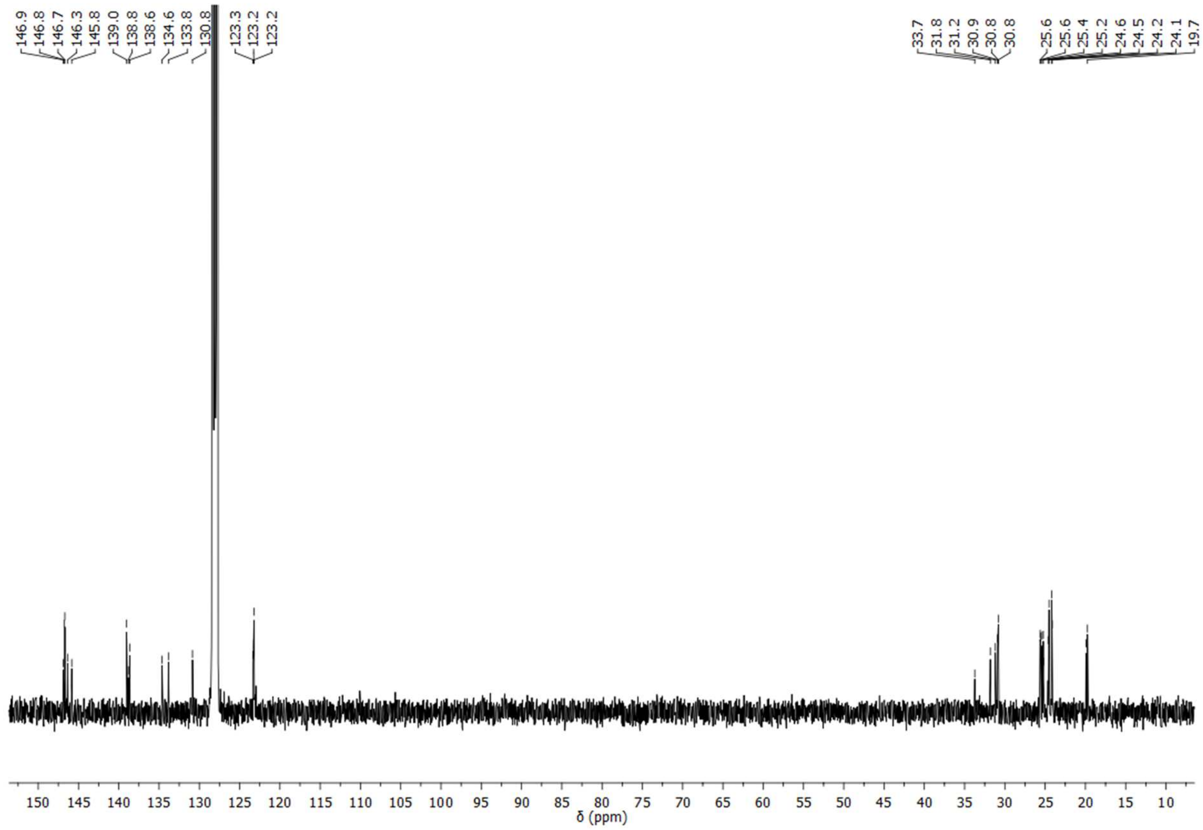

Figure S65: ^{13}C NMR spectrum of **13** in C_6D_6 at 25 °C.

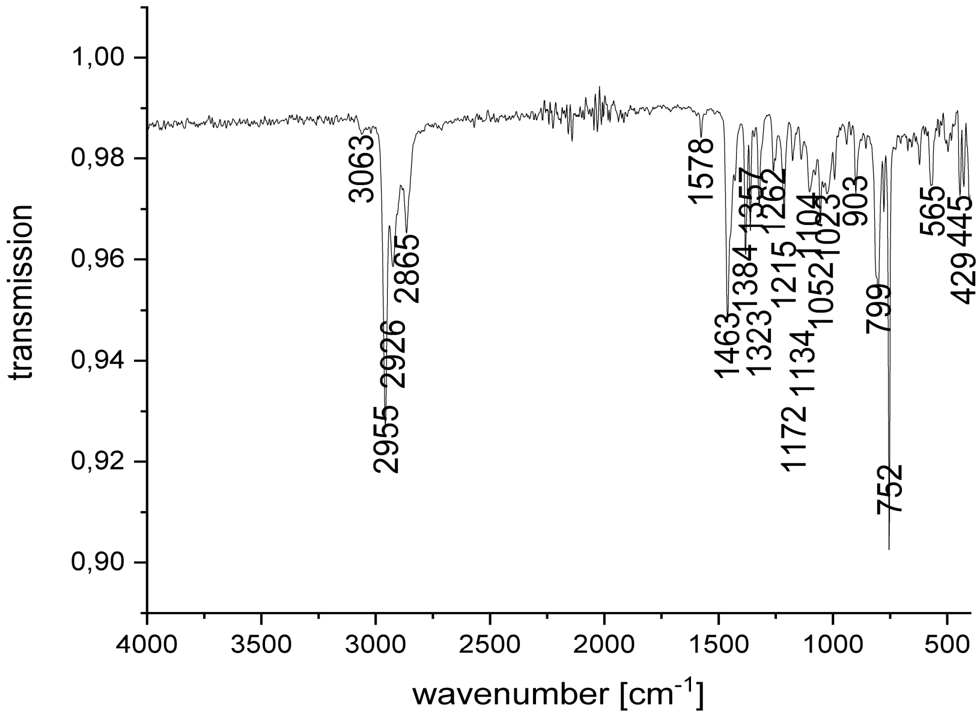

Figure S66: ATR-IR spectrum of **13**.

Comparison of IR spectra

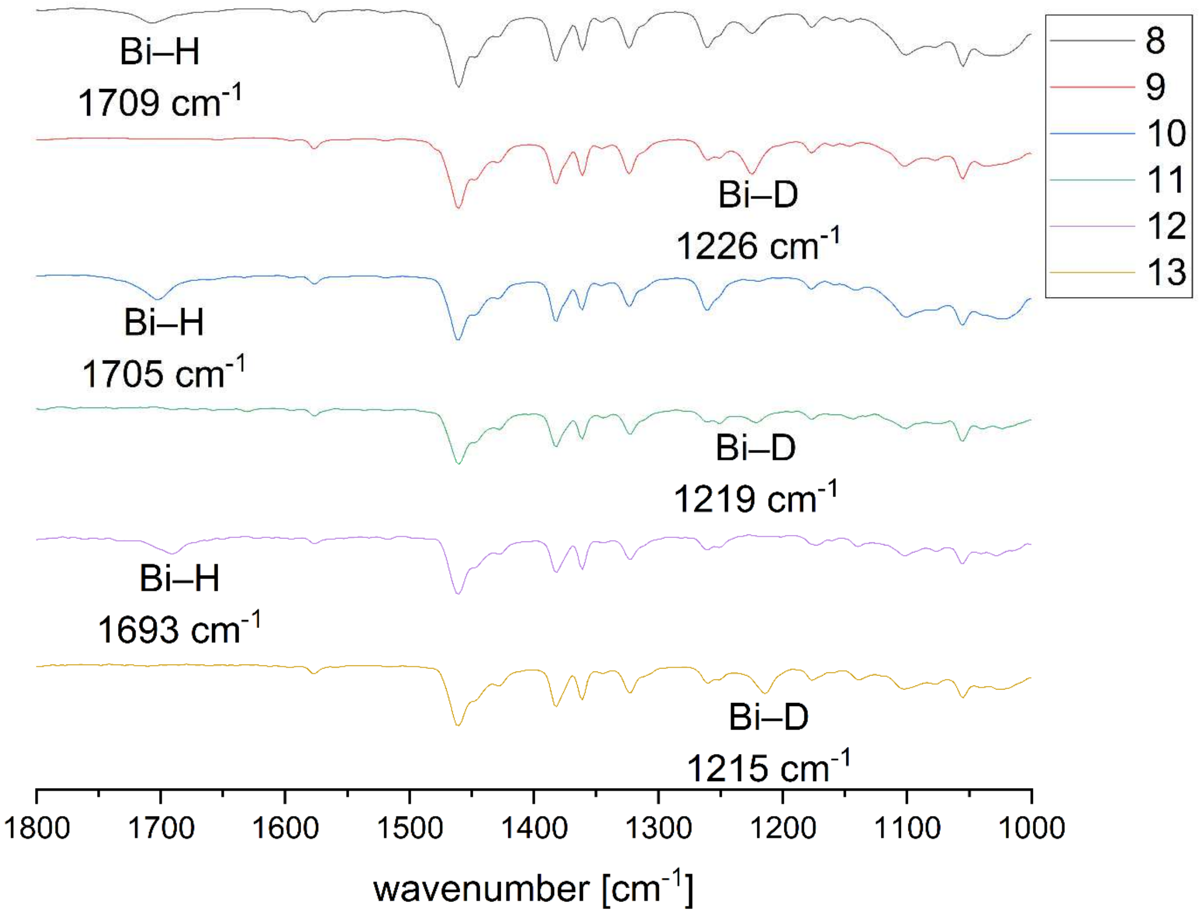

Figure S67: Comparison of IR spectra of **8** – **13**. Assignment of Bi–H and Bi–D stretching bands.

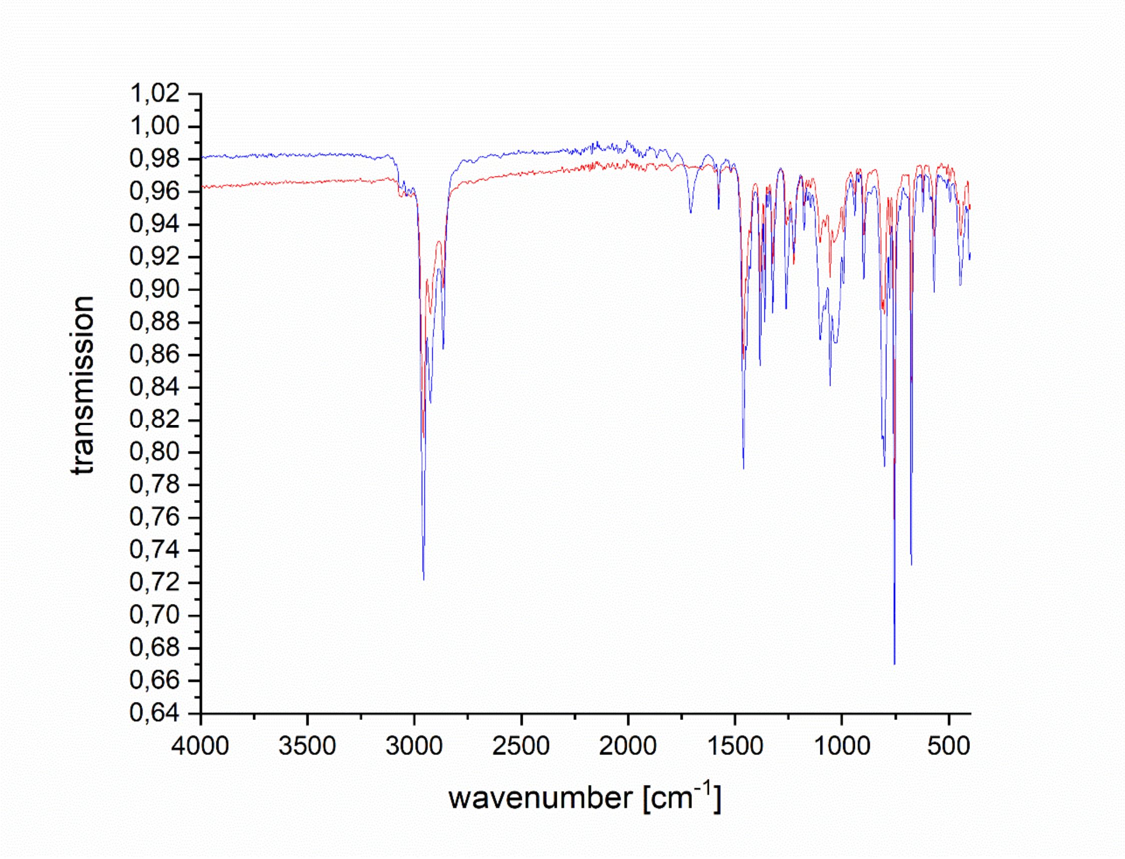

Figure S68: Overlay of IR spectra of **8** (blue) and **9** (red).

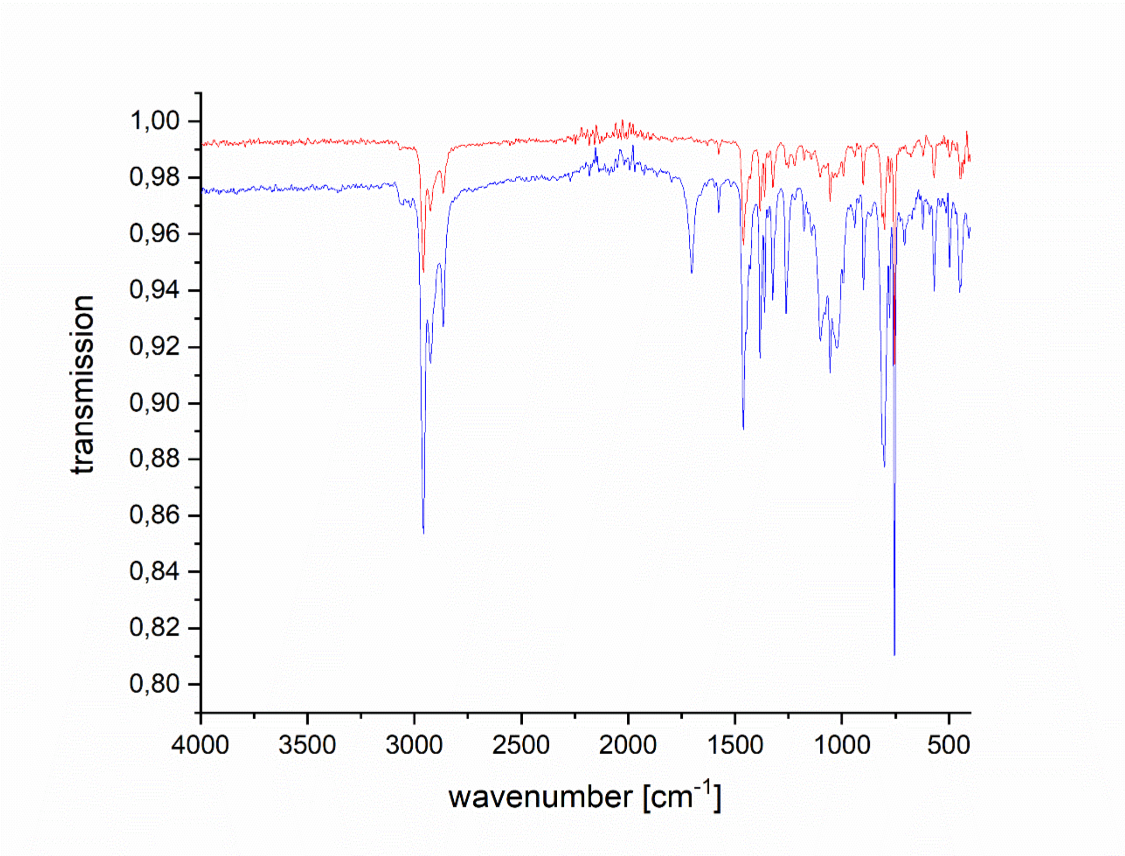

Figure S69: Overlay of IR spectra of **10** (blue) and **11** (red).

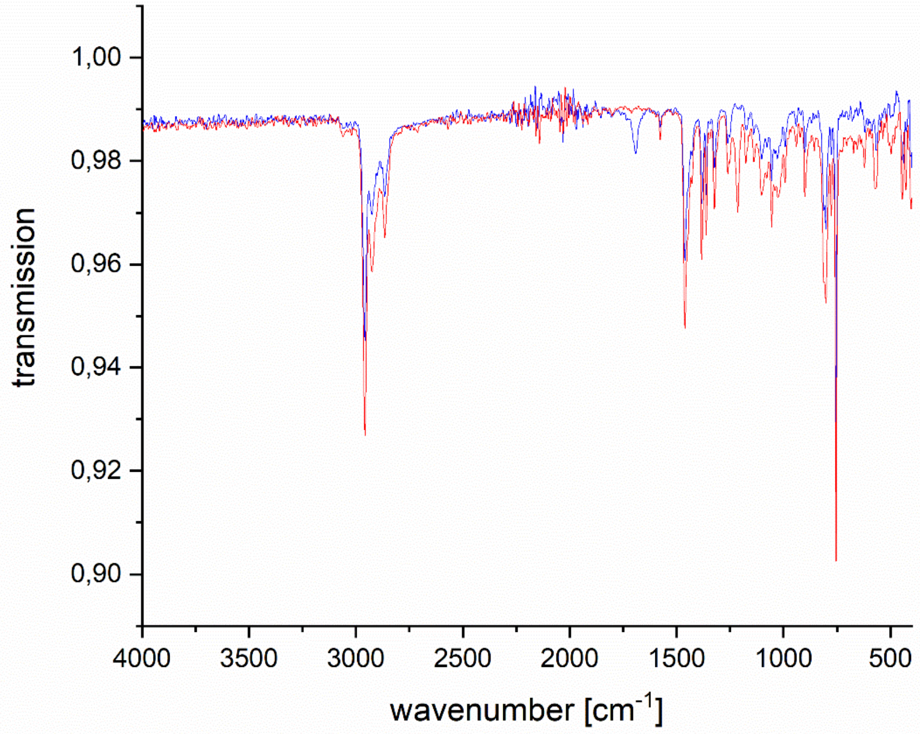

Figure S70: Overlay of IR spectra of **12** (blue) and **13** (red).

III. Crystallographic Details

The crystals were mounted on nylon loops in inert oil. Data of **Ar*-7**, **Ar*I**, and **Ar*Li·Li*t*-Bu** were collected on a Bruker AXS D8 Venture diffractometer with Photon II detector (mono-chromated Cu_{Ka} radiation, $l = 1.54178$ Å, mircofocus source) at 100(2) K while those of **Ar*H**, **1**, **4**, and **12** were collected on a Bruker AXS D8 Kappa diffractometer with APEX2 detector (mono-chromated Mo_{Ka} radiation, $l = 0.71073$ Å) at 100(2) K. The structures were solved by Direct Methods (SHELXS-2013)[3] and refined anisotropically by full-matrix least-squares on F^2 (SHELXL-2017).[4,5] Absorption corrections were performed semi-empirically from equivalent reflections on basis of multi-scans and in case of **12** numercally from indexed faces (Bruker AXS APEX3). Hydrogen atoms were refined using a riding model or rigid methyl groups. The OH hydrogen atoms in **2** could not be identified but are included in the sum formula for completeness. The methyl groups of the central BiHiPr moieties of **12** were refined with ideally staggerd orientations (AFIX 33). The BiH hydrogen atoms could not be identified but were included in the sum formula for completeness. **Ar*Li·Li*t*-Bu** is co-crystallised with approx. 15% of the free ligand. Except for a dipp and an isopropyl group, the free and the coordinated ligand share the same position. The Li_2*t*-Bu moiety is disordered over two positions which is correlated to a disorder of an isopropyl group. The phenyl ring of the alternate position of the free ligand was constrained to a regular hexagon of 1.39 Å edge lengths (AFIX 66) and all corresponding bond lengths and most angles of disordered atoms were restrained to be equal (SADI). RIGU restraints were applied to the displacement parameters of the disordered atoms. Additional SIMU restraints or EADP constraints were used in cases of strong correlation. Considering the vast use of re- and constraints quantitative results may be biassed and unreliable and should be carefully assessed. The crystal of **Ar*H** contained about 2% of**Ar*I**. This correlates with the disordered solvent which was refined using three alternate positions. Its bond lengths and angles were restrained to be equal (SADI) and RIGU and SIMU restraints were applied to its displacement parameters. The C1 of each residue were refined with common displacement parameters (EADP) due to their close proximity. The crystal was twinned and refined as a 2-component twin against HKLF5 data. A low cut at $d = 9$ Å was applied to exclude reflections shaded by the beamstop. The overall quality of the structure is low and results beyond the connectivity should be carefully assessed. The benzene molecule in **1** is disordered and could be crudely modelled by two alternate positions. The occupancies were constrained to 0.7 and 0.3, respectively, to avoid correlations. The bond lengths were restrained to be equal to 1.39 Å (DFIX) and the bond angles were restrained to be equal (SADI). In addition, the molecule was restrained to planarity (FLAT). RIGU and SIMU restraints were applied to the displacement parameters. The high R_{int} suggests rather poor data quality. Quantitative results should be carefully assessed. The $Bi(OH)_2$ moiety ins **2** is disordered over a mirror plane. The local symmetry was ignored in the refinement of the oxygen atoms (negative PART). One solvent molecule is disordered over a mirror plane the other over centres of inversion forming a diffusely disordered string of solvent parallel to *b*. The displacement parameters of these molecules were constrained to be equal (EADP). The bond lengths and angles of all solvent molecules were restrained to be equal (SADI) and RIGU and SIMU restraints were applied to their displacement parameters. Especially at high θ a high number of reflections have intensities lower than $2\sigma(I)$. Consequently, quantitative results should be carefully assessed and may be unreliable. Considering the disorder of the most interesting parameters one should best only discuss the connectivity. In **4** an isopropyl group is disordered over two positions. The isopropyl groups of the central moiety of **12** and another one of the ligand are disordered over two positions. Their displacement parameters were refined with RIGU restraints and an additional SIMU restraint for the central moieties. Especially at high θ a high number of reflections have intensities lower that $2\sigma(I)$. Consequently, quantitative results should be carefully assessed and may be unreliable.

CCDC-2421213(**1** ys_855m), -2441531 (**2** ys_867m), -2421214(**4** ys_860bm), -2421215(**12** ys_921_molym), -2421231(**Ar*-7** ys_terphenylbrm), -2421232(**Ar*I** ys_796m), -2421233(**Ar*Li·Li*t*-Bu** ys_814m), and -2421234(**Ar*H** ys_925_tw5) contain the supplementary crystallographic data for this paper. These data can be obtained free of charge from The Cambridge Crystallographic Data Centre via www.ccdc.cam.ac.uk/data_request/cif.

Identification code	**Ar*-7** ys_terphenylbrm	**Ar*I** ys_796m	**Ar*Li·Li*t*-Bu** ys_814m	**Ar*H** ys_925_tw5
Empirical formula	$C_{32}H_{41}Br$	$C_{83}H_{105}I$	$C_{79.46}H_{104.65}Li_{1.73}$	$C_{82.86}H_{113.66}I_{0.02}$
M	505.56	1229.56	1071.88	1112.24
Crystal size [mm]	0.500 × 0.100 × 0.100	0.300 × 0.262 × 0.122	0.269 × 0.172 × 0.115	0.755 × 0.425 × 0.218
T [K]	103(2)	100(2)	100(2)	100(2)
Crystal system	orthorhombic	monoclinic	triclinic	triclinic
Space group	*Pbca*	$P2_1/c$	*P*-1	*P*-1
a [Å]	16.9045(8)	21.1653(12)	12.6682(3)	13.2783(17)
b [Å]	15.4827(8)	16.4728(9)	15.2355(6)	13.5017(17)
c [Å]	21.0840(11)	22.4330(13)	18.9054(7)	22.250(3)
α [°]	90	90	89.349(2)	105.883(6)
β [°]	90	112.840(3)	87.296(3)	98.147(6)
γ [°]	90	90	70.445(2)	103.025(6)
V [Å^3]	5518.3(5)	7208.0(7)	3434.5(2)	3648.8(8)
Z	8	4	2	2
D_{calc} [g·cm^{-3}]	1.217	1.133	1.036	1.012
μ(CuK_α [mm^{-1}])	2.132	3.786	0.421	0.064
Transmissions	0.75/0.60	0.41/0.20	0.75/0.64	0.75/0.65
F(000)	2144	2624	1173	1224
Index ranges	$-21 \leq h \leq 21$	$-26 \leq h \leq 27$	$-16 \leq h \leq 16$	$-20 \leq h \leq 20$
	$-19 \leq k \leq 15$	$-20 \leq k \leq 21$	$-19 \leq k \leq 19$	$-20 \leq k \leq 19$
	$-26 \leq l \leq 26$	$-27 \leq l \leq 27$	$-24 \leq l \leq 24$	$0 \leq l \leq 34$
θ_{max} [°]	77.490	81.142	81.276	33.378
Reflections collected	76394	244658	182624	275083
Independent reflections	5867	15576	14982	26796
R_{int}	0.0369	0.0683	0.0436	0.0956
Refined parameters	308	782	893	906
R_1 [$I > 2\sigma(I)$]	0.0235	0.0572	0.0592	0.0962
wR_2 [all data]	0.0654	0.1830	0.1732	0.2209
GooF	1.054	1.037	1.030	1.064
$\Delta\rho_{final}$ (max/min) [e·Å^{-3}]	0.388/-0.353	2.143/-0.656	0.465/-0.398	0.647/-0.388

Identification code	**1** ys_855m	**2** ys_867m	**4** ys_860bm	**12** ys_921_molym
Empirical formula	$C_{86}H_{115}BiN_2$	$C_{88}H_{111}BiO_2$	$C_{82}H_{97}BiF_6$	$C_{79}H_{105}Bi$
M	1385.77	1409.74	1405.57	1262.60
Crystal size [mm]	0.118 × 0.081 × 0.050	0.349 × 0.075 × 0.050	0.444 × 0.140 × 0.086	0.366 × 0.310 × 0.100
T [K]	100(2)	100(2)	100(2)	100(2)
Crystal system	monoclinic	monoclinic	triclinic	triclinic
Space group	$P2_1/c$	*P 21/m*	*P*-1	*P*-1
a [Å]	17.198(2)	14.8472(7)	12.780(7)	12.6843(15)
b [Å]	18.462(2)	18.1835(8)	17.048(10)	15.6586(18)
c [Å]	24.110(3)	15.9361(8)	17.202(13)	37.118(4)
α [°]	90	90	88.081(16)	88.218(3)
β [°]	101.430(4)	114.186(2)	74.700(11)	88.332(3)
γ [°]	90	90	89.198(9)	70.279(3)
V [Å^3]	7503.0(16)	3924.7(3)	3613(4)	6935.3(14)
Z	4	2	2	4
D_{calc} [g·cm^{-3}]	1.227	1.193	1.292	1.209
μ(MoK_α [mm^{-1}])	2.394	2.290	2.497	2.582
Transmissions	0.75/0.67	0.37/0.30	0.75/0.66	0.41/0.22
F(000)	2912	1476	1452	2644
Index ranges	$-24 \le h \le 24$	$-19 \le h \le 19$	$-19 \le h \le 19$	$-18 \le h \le 18$
	$-26 \le k \le 26$	$-24 \le k \le 24$	$-26 \le k \le 26$	$-22 \le k \le 22$
	$-34 \le l \le 34$	$-21 \le l \le 21$	$-26 \le l \le 26$	$-53 \le l \le 53$
θ_{max} [°]	30.656	28.385	33.310	30.804
Reflections collected	231640	137243	287751	277040
Independent reflections	23077	10103	27665	43037
R_{int}	0.1697	0.0833	0.1222	0.0628
Refined parameters	884	468	847	1557
R_1 [$I > 2\sigma(I)$]	0.0525	0.0513	0.0519	0.0669
wR_2 [all data]	0.1372	0.1392	0.1338	0.1595
GooF	1.056	1.068	1.054	1.097
$\Delta\rho_{final}$ (max/min) [e·Å^{-3}]	3.335/-1.922	6.356/-1.206	1.675/-1.621	5.268/-6.034

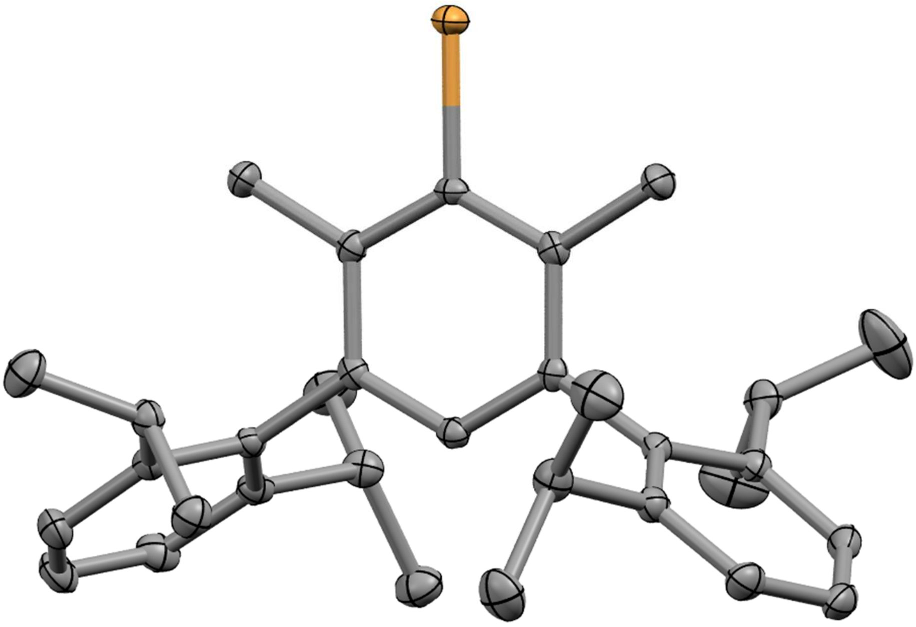

Figure S71: Molecular structure of **Ar*-7** in the solid state, crystallized from DCM/methanol. Ellipsoids are drawn at a probability level of 50%. Hydrogen atoms are omitted for clarity. Carbon atoms are depicted in grey, and a bromine atom in brown.

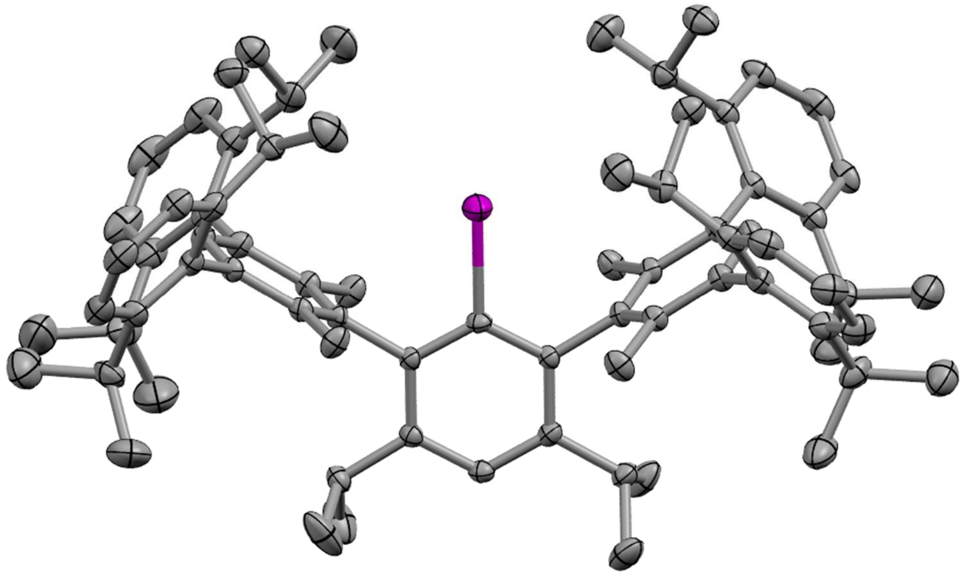

Figure S72: Molecular structure of **Ar*I** in the solid state, crystallized from DCM/methanol. Ellipsoids are drawn at a probability level of 50%. Hydrogen atoms and solvent molecules are omitted for clarity. Carbon atoms are depicted in grey, and an iodine atom in purple.

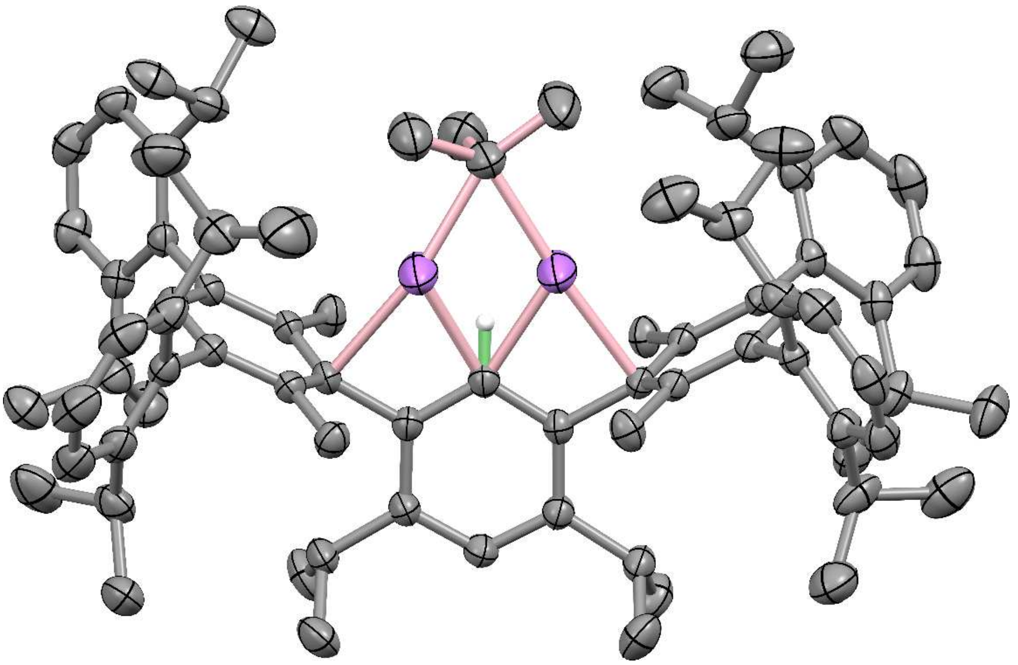

Figure S73: Molecular structure of **Ar*Li·Li*t*-Bu** in the solid state, crystallized from toluene. Ellipsoids are drawn at a probability level of 50%. Hydrogen atoms and minor components of disorder are omitted for clarity. Carbon atoms are depicted in grey, and lithium atoms in purple. The molecule is disordered over two alternate positions which make up about 85% and in 15% the Li_2tBu moiety is replaced by an H atom in the ipso position, i.e. about 15% of the crystal consists of the free ligand. This means that the crystal is a co-crystal of the product (85%) and a by-product (15%).

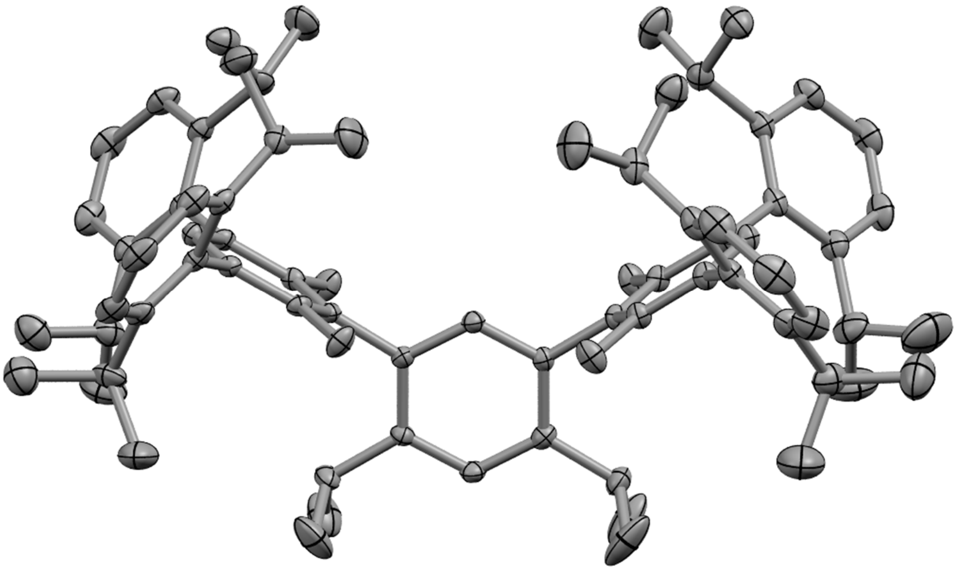

Figure S74: Molecular structure of **Ar*H** in the solid state, crystallized from *n*-heptane. Ellipsoids are drawn at a probability level of 50%. Hydrogen atoms, minor components of disorder, and solvent molecules are omitted for clarity.

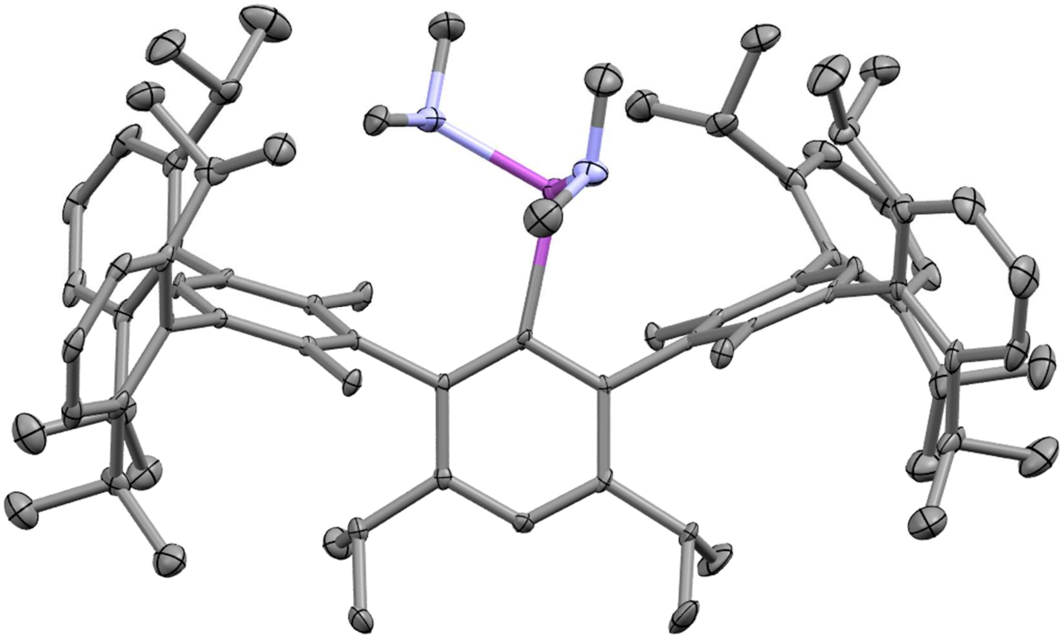

Figure S75: Molecular structure of **1** in the solid state, crystallized from benzene. Ellipsoids are drawn at a probability level of 50%. Hydrogen atoms and solvent molecules are omitted for clarity. Carbon atoms are depicted in grey, a bismuth atom in purple, and nitrogen atoms in blue.

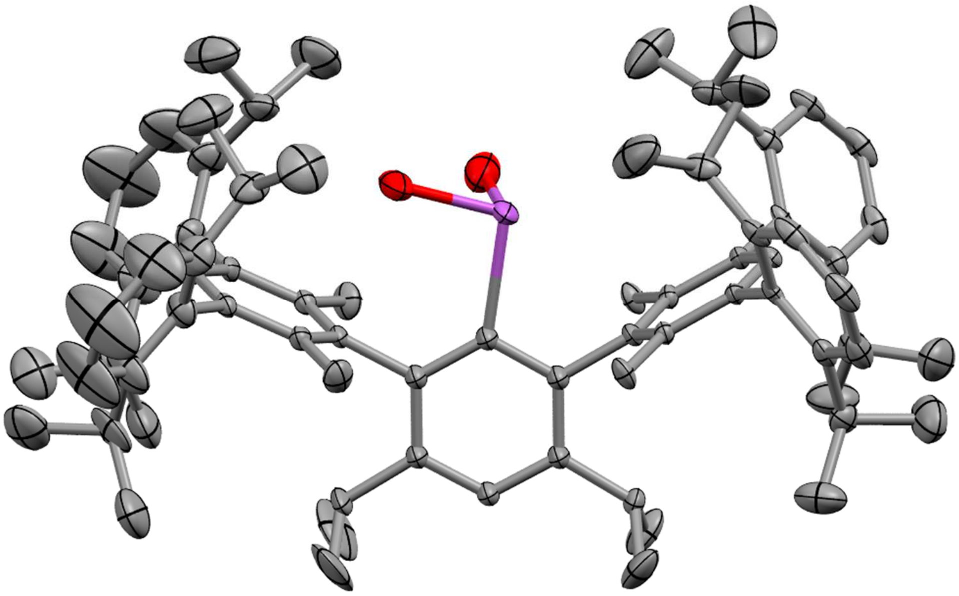

Figure S76: Molecular structure of **2** in the solid state, crystallized from benzene. Ellipsoids are drawn at a probability level of 50%. Hydrogen atoms and one component of disorder are omitted for clarity. Carbon atoms are depicted in grey, a bismuth atom in purple, and oxygen atoms in red.

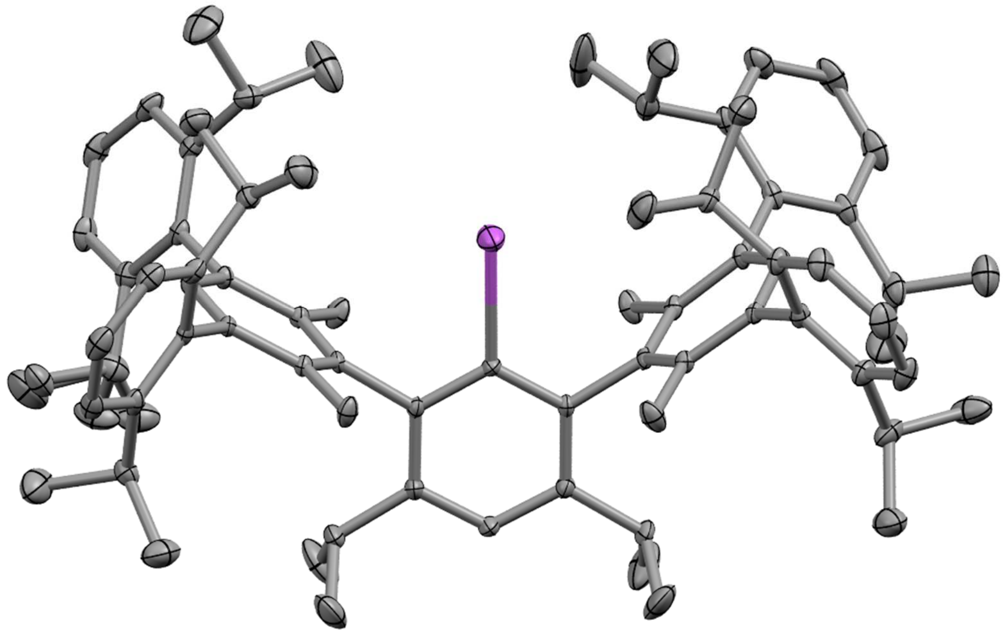

Figure S77: Molecular structure of **4** in the solid state, crystallized from hexafluorobenzene. Ellipsoids are drawn at a probability level of 50%. Hydrogen atoms and a solvent molecule are omitted for clarity. Carbon atoms are depicted in grey, and a bismuth atom in purple. (Crystals from benzene were always twinned and crystals from toluene diffracted poorly.)

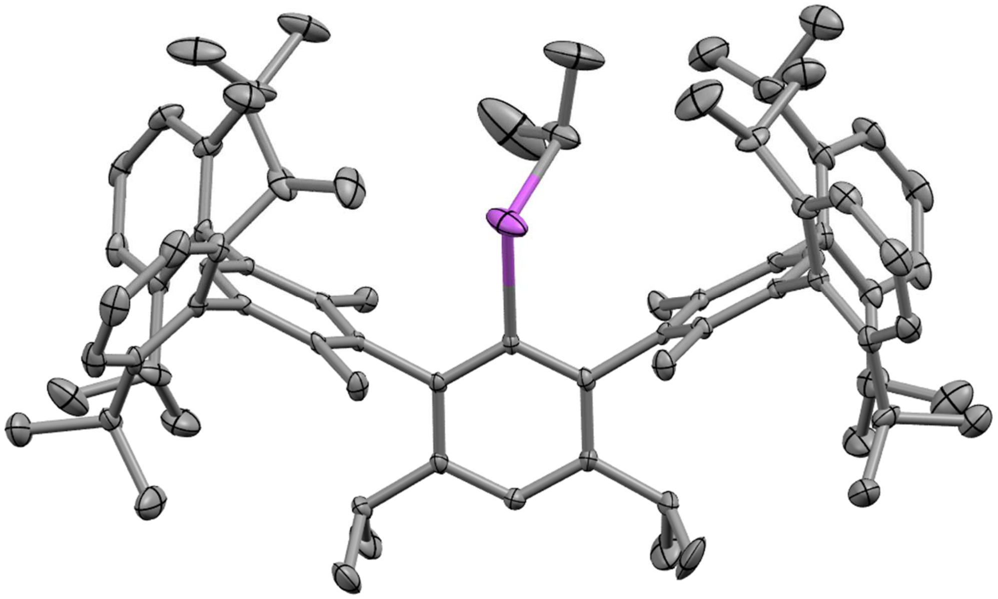

Figure S78: Molecular structure of **12** in the solid state, crystallized from hexane. Ellipsoids are drawn at a probability level of 50%. Hydrogen atoms and another nearly identical molecule in the asymmetric unit are omitted for clarity. Carbon atoms are depicted in grey, and a bismuth atom in purple.

IV. Buried Volume Calculations

The program SambVca 2.1[5] (https://www.aocdweb.com/OMtools/sambvca2.1/) was used with the following settings:
Select the atoms coordinated to the center of the sphere: the bismuth atom
Select the atoms for z axis definition: carbon atom bound to bismuth atom; z-axis negative
Select the atoms for xz-plane definition: carbon atom 2 (*ortho*)
Select the atoms to be deleted: none
Select the atomic radii: Bondi radii scaled by 1.17
Sphere radius: 3.5 Å
Distance of the coordination point from the center of the sphere: 0
Mesh spacing for numerical integration: 0.1 Å
Check the box below to include H atoms in the calculations: checked

Ar*Bi:

%V Free	**%V Buried**	% V Tot/V Ex
25.4	**74.6**	99.9

Quadrant	V f	V b	V t	%V f	**%V b**
SW	14.5	30.4	44.9	**32.2**	67.8
NW	13.8	31.0	44.9	**30.8**	69.2
NE	6.3	38.5	44.9	**14.1**	85.9
SE	11.0	33.8	44.9	**24.6**	75.4

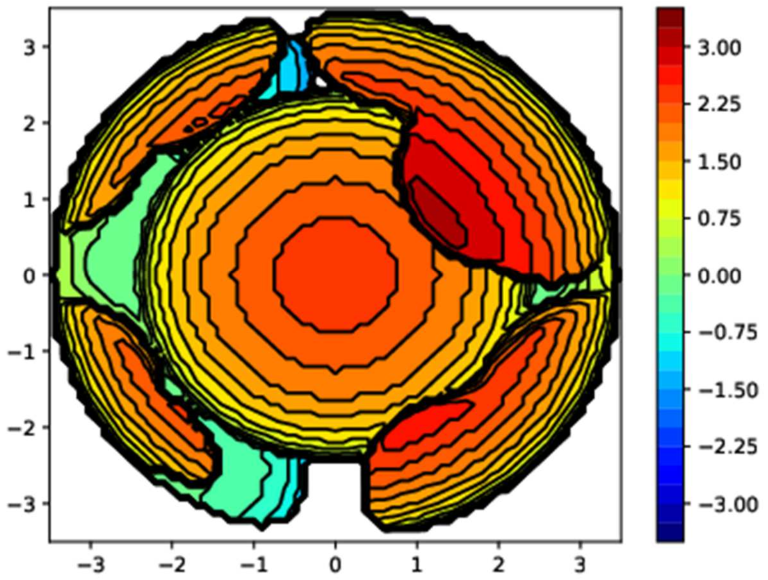

$M^sFluInd^{tBu}Bi$:

%V Free	**%V Buried**	% V Tot/V Ex
31.0	**69.0**	99.9

Quadrant	V f	V b	V t	%V f	**%V b**
SW	14.4	30.4	44.9	**32.1**	67.9
NW	13.4	31.5	44.9	**29.8**	70.2
NE	12.0	32.9	44.9	**26.7**	73.3
SE	15.9	28.9	44.9	**35.5**	64.5

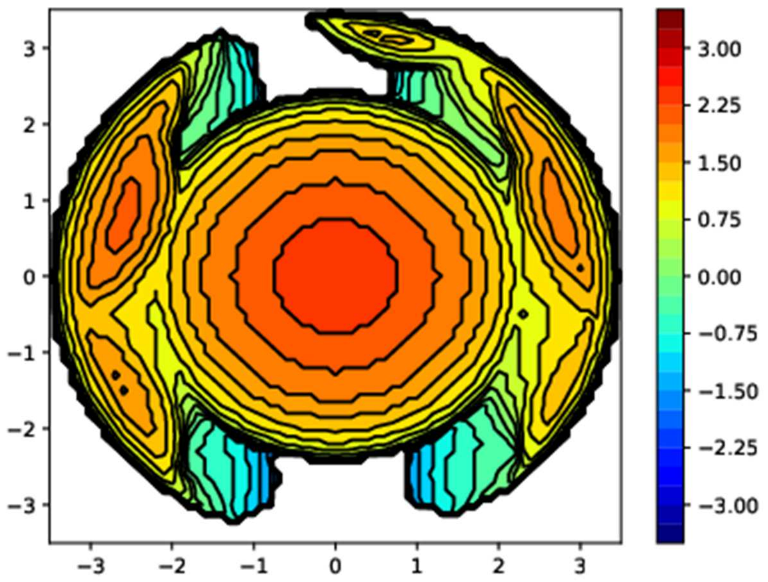

M^{s}FluInd*Bi:

%V Free	**%V Buried**	% V Tot/V Ex
25.3	**74.7**	99.9

Quadrant	V f	V b	V t	%V f	**%V b**
SW	8.0	36.9	44.9	**17.7**	82.3
NW	14.1	30.8	44.9	**31.3**	68.7
NE	9.6	35.2	44.9	**21.5**	78.5
SE	13.8	31.0	44.9	**30.8**	69.2

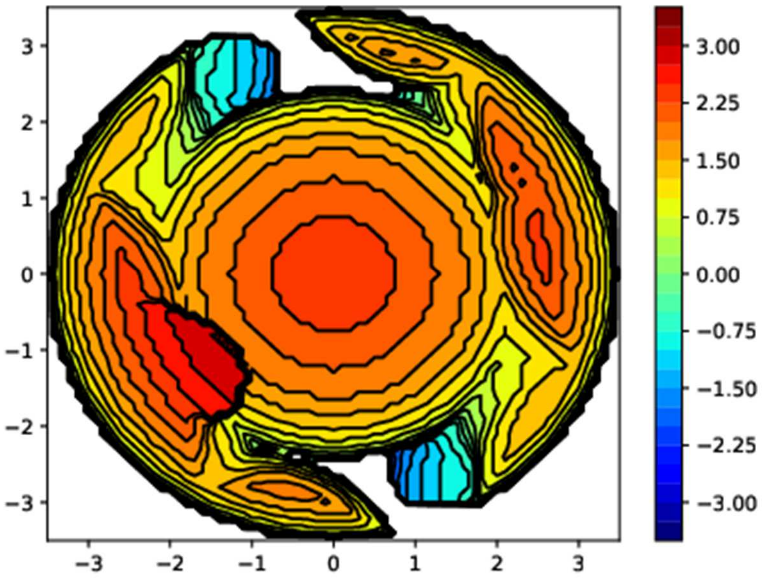

V. Computational Details

All calculations were performed by using the program packages Gaussian 16[7] and Orca 6.0[8]. The geometrical parameters of **4** and **8** were optimized by means of B3LYP[9-11] with additional dispersion correction D3BJ[12]. The basis sets applied were def2-SVP[13-14] for the light elements (H and C) and def2-TZVP[15] for bismuth. In the case of **4**, three structures were optimized assuming a closed-shell singlet ground state, a triplet ground state, and an open-shell singlet electronic ground state. For the latter, the open-shell variant UDFT was used as implemented in Gaussian via the "guess=mix" command. This command requests the HOMO and LUMO to be mixed to break α-β and spatial symmetries, which is useful for generating UHF wave functions for singlet states. For all structures C_1 symmetry was applied. Frequency calculations were carried out at each of the stationary points to verify the nature of the stationary point. It turned out that all products have no imaginary frequency. Furthermore, the energies of the three states of **4** were computed using the density functionals B3LYP and the basis sets def2-TZVP[13-14] for all elements.

For a more detailed analysis of the electronic structure, a geometry optimization with B3LYP-D3BJ/def2-TZVP was performed for the triplet ground state without symmetry restriction. Frequency calculation revealed that the structure is a true minimum without any negative frequency. Scalar relativistic complete active space self-consistent field (CASSCF) and *n*-electron valence second-order perturbation theory (NEVPT2[16]) calculations were carried out at the DFT-optimized geometry using the X2C scalar relativistic Hamiltonian[17] and the X2C-TZVPPall[18-19] basis set. Spin-orbit coupling (SOC) was treated using the mean-field SOC Hamiltonian[20-21] including picture change effects.

Table S1. Energies for the transition from ground state (state 1) to the excited states 2–21 calculated using NEVPT2.

Transition	Energy [eV]	Energy [cm^{-1}]	Wavelength [nm]
1 → 2	0.5138	4144	2412.9
1 → 3	0.5507	4442	2251.3
1 → 4	1.2119	9774	1023.1
1 → 5	1.2195	9836	1016.7
1 → 6	2.5450	20527	487.2
1 → 7	3.8293	30885	323.8
1 → 8	3.8321	30908	323.5
1 → 9	3.9082	31521	317.2
1 → 10	3.9838	32131	311.2
1 → 11	4.1273	33289	300.4
1 → 12	4.2733	34466	290.1
1 → 13	4.3394	34999	285.7
1 → 14	4.5509	36705	272.4
1 → 15	4.8597	39196	255.1
1 → 16	5.0289	40561	246.5
1 → 17	5.0628	40834	244.9
1 → 18	5.0795	40969	244.1
1 → 19	5.3269	42964	232.8
1 → 20	5.3307	42995	232.6
1 → 21	5.5288	44593	224.3

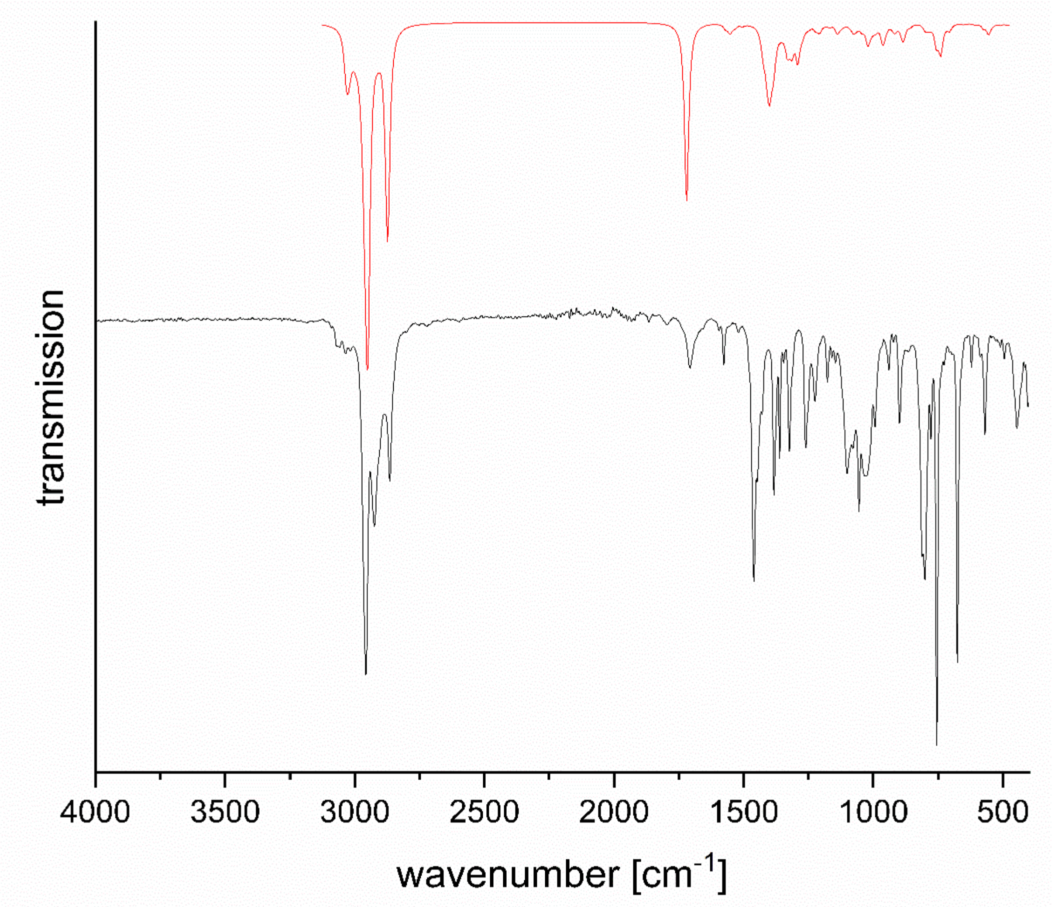

Figure S79: Overlay of measured (black) and calculated IR spectra of compound **8** simulated by means of B3LYP-D3BJ. The scale factor was determined to a value of 0.9486. The stretching frequency for the Bi–H bond is calculated to a value of 1718 cm^{-1}.

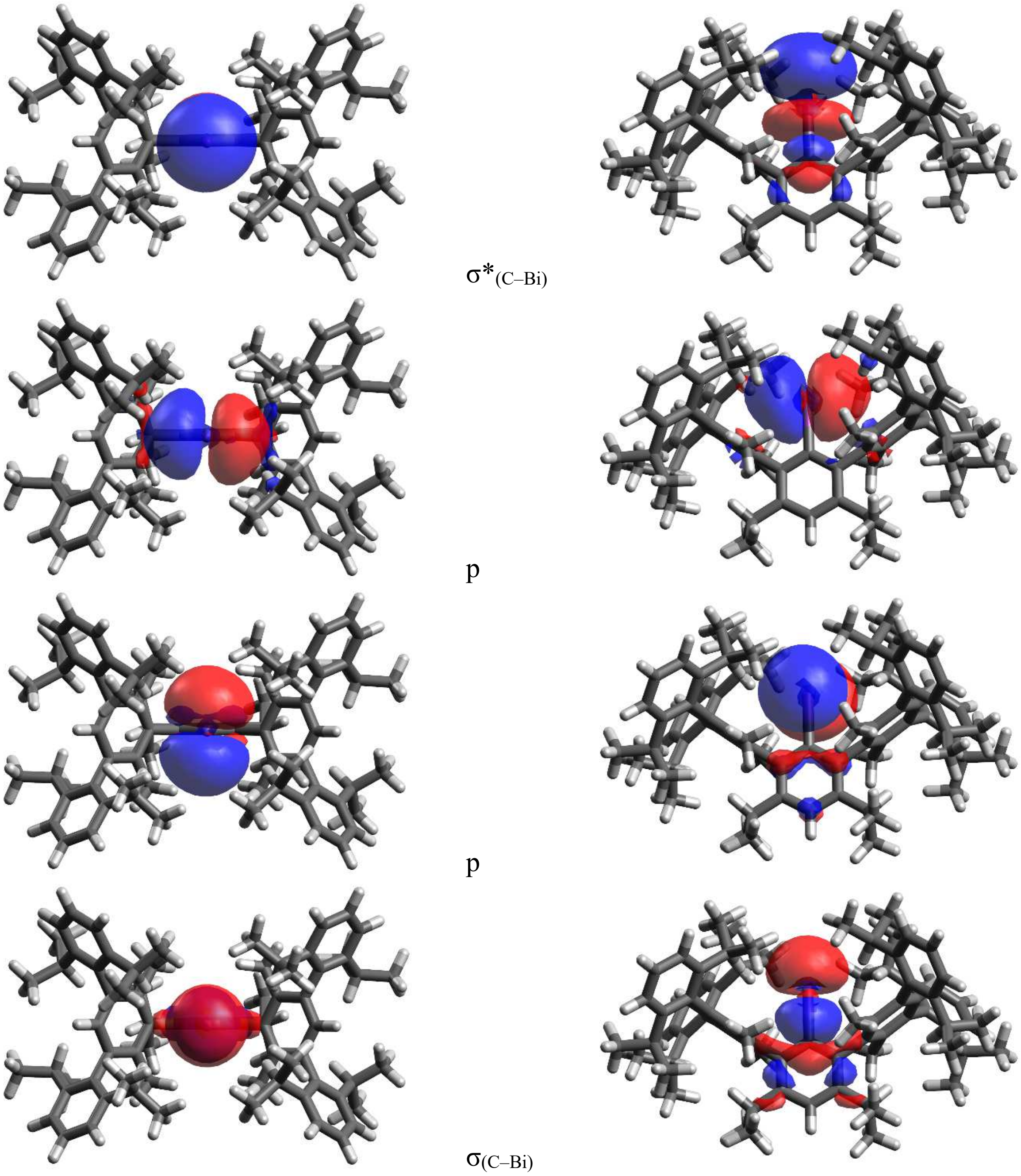

Figure S80: The active orbitals from the CASSCF(4,4) calculation of bismuthinidene **4**, with visual representations from different perspectives (side and top view).

state	relative energy	configuration
state 1 ($S = 1, M_S = 0$)	0 cm^{-1}	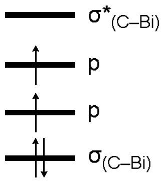
state 2 ($S = 1, M_S = \pm1$)	4144 cm^{-1} / 2413 nm	
state 3 ($S = 1, M_S = \pm1$)	4442 cm^{-1} / 2251 nm	
state 4 ($S = 0, M_S = 0$)	9774 cm^{-1} / 1023 nm	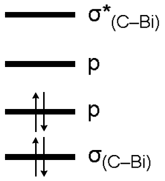
state 5 ($S = 0, M_S = 0$)	9836 cm^{-1} / 1017 nm	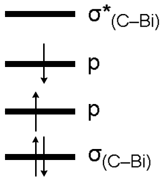
state 6 ($S = 0, M_S = 0$)	20527 cm^{-1} / 487 nm	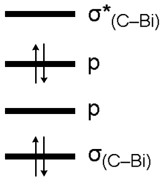

Figure S81: Lowest states of **4** calculated by means of NEVPT2. The energies of the states are given in relation to state 1.

Cartesian Coordinates for the Calculated Compounds

Cartesian coordinates of the optimized structure of **8** at B3LYP-D3BJ/def2-SVP,def2-TZVP level of theory (number of imaginary frequencies = 0). E = -3208.166340 au

Bi	-0.15868200	-0.07535100	-1.25534100
C	0.02448100	-0.24862700	1.03015500
C	-1.19546800	-0.30284300	1.73282200
C	-1.20423900	-0.44215900	3.13640600
C	0.02351000	-0.51679000	3.80179100
H	0.02543500	-0.62578600	4.88920600
C	1.24963400	-0.44470600	3.13226800
C	1.24638400	-0.30800300	1.72665400
C	-2.50652400	-0.49881600	3.92556700
H	-3.33237900	-0.43433100	3.20150700
C	-2.64398700	0.69806500	4.87841800
H	-3.62022600	0.68023700	5.38923700
H	-2.56503500	1.65207800	4.33583700
H	-1.85973600	0.68699100	5.65262300
C	-2.65905900	-1.82898300	4.67807100
H	-2.57221400	-2.68573600	3.99345900
H	-3.64153600	-1.88570300	5.17422600
H	-1.88601200	-1.94242600	5.45543600
C	2.55067900	-0.50772100	3.92393900
H	3.37746800	-0.46035800	3.20049200
C	2.70112500	0.69851200	4.86290400
H	1.90891100	0.71385900	5.62902600
H	2.64799100	1.64619000	4.30626100
H	3.67149700	0.66828300	5.38447000
C	2.68784200	-1.83212000	4.68929300
H	2.59560200	-2.69386800	4.01150600
H	1.91243600	-1.93213400	5.46622600
H	3.66854300	-1.89325100	5.18837900
C	-2.47932700	-0.16808300	0.97402300
C	-2.94778300	1.13397000	0.67756900
C	-4.12993200	1.28240700	-0.06963700
C	-4.80752400	0.13841800	-0.50891500
H	-5.72645700	0.25915200	-1.08665100
C	-4.36000400	-1.14761900	-0.20667800
C	-3.18186000	-1.31271800	0.55091400
C	-2.15868100	2.33674300	1.13299300
H	-1.24099200	2.45312000	0.53200900
H	-1.82659900	2.23136400	2.17477600
H	-2.73869300	3.26190600	1.03386300
C	-2.72575700	-2.70076800	0.92452300
H	-2.82198500	-3.39550800	0.07847300
H	-3.35847900	-3.11024900	1.73001200
H	-1.68710800	-2.70953300	1.27584100
C	-4.69454700	2.63006300	-0.38837000
C	-4.71510400	4.63820300	-1.74353800
H	-4.32317300	5.22327400	-2.57884900
C	-4.17283700	3.37497400	-1.46842500
C	-5.74950200	5.15559200	-0.96776200
H	-6.16135300	6.14280500	-1.19196100
C	-6.26433100	4.41082200	0.09392900
H	-7.07655900	4.82648600	0.69242300
C	-5.75417600	3.14263500	0.39960800
C	-3.57863200	2.49454300	-3.75620700
H	-2.78706000	2.02526700	-4.36324100
H	-3.91253300	3.40423700	-4.28151400
H	-4.43085800	1.80019900	-3.71309300
C	-3.05992500	2.82112800	-2.34804800
H	-2.72563000	1.87337900	-1.90358400
C	-1.83948100	3.74946500	-2.40476600
H	-1.02440800	3.28007100	-2.97731400
H	-1.45848000	3.97232100	-1.39682900
H	-2.07769800	4.70825900	-2.89271900
C	-6.29089700	2.35515200	1.59144700
H	-6.13964600	1.28884700	1.37204600
C	-5.48248000	2.66842500	2.86139600

H	-5.84551700	2.07159700	3.71409000
H	-5.57004100	3.73445000	3.12783100
H	-4.41651800	2.44217300	2.72321800
C	-7.79081000	2.55298600	1.83406200
H	-8.37533100	2.36586600	0.91995100
H	-8.02407400	3.57178500	2.18262700
H	-8.14290100	1.85684700	2.61136700
C	-5.15531400	-2.33255700	-0.65045400
C	-6.15965100	-2.84546600	0.19959500
C	-6.91632200	-3.94184900	-0.23597400
H	-7.70291100	-4.34589400	0.40516000
C	-6.67767900	-4.52493800	-1.47924000
H	-7.27581800	-5.37927900	-1.80599400
C	-5.67145600	-4.02291400	-2.30468400
H	-5.48942600	-4.49231900	-3.27331600
C	-4.89442700	-2.92767000	-1.90565500
C	-6.44520000	-2.20108700	1.55097500
H	-5.60283000	-1.53178600	1.78031000
C	-7.70754800	-1.32773800	1.48077600
H	-8.59669300	-1.93738900	1.25128300
H	-7.61643700	-0.56202100	0.69605800
H	-7.88526900	-0.81352400	2.43921800
C	-6.53324000	-3.22228100	2.69244900
H	-7.40316200	-3.88940700	2.58304000
H	-6.63540100	-2.70729800	3.66124000
H	-5.63117600	-3.85240300	2.73510700
C	-3.81409300	-2.36319300	-2.82032600
H	-3.11632100	-1.79595500	-2.18576500
C	-4.41762200	-1.36796100	-3.82376900
H	-3.63170200	-0.91267500	-4.44806400
H	-4.95028600	-0.55727400	-3.30653100
H	-5.13635000	-1.87265500	-4.48985300
C	-2.99930900	-3.44407600	-3.53978400
H	-3.60437700	-3.99143200	-4.27993300
H	-2.58672800	-4.17816500	-2.83097800
H	-2.15705000	-2.98708500	-4.08248100
C	2.53639300	-0.18440500	0.98348000
C	3.23926700	-1.33347900	0.58405300
C	4.42616200	-1.18017000	-0.16085600
C	4.88261900	0.10033300	-0.47061100
H	5.80545500	0.21321000	-1.04348000
C	4.19528200	1.24937600	-0.06148400
C	3.00473300	1.11180800	0.67177800
C	2.76430300	-2.72010100	0.93853400
H	1.75865200	-2.71000600	1.37516600
H	3.44749800	-3.19347700	1.66348200
H	2.75926900	-3.37561700	0.05521100
C	2.20272300	2.31752000	1.09739600
H	1.23154700	2.34848500	0.57755000
H	2.73112100	3.25303800	0.87995900
H	1.96573200	2.28492400	2.17063000
C	5.20235900	-2.37306200	-0.61461900
C	4.90913600	-2.96141200	-1.86592200
C	5.67413300	-4.05696500	-2.28726900
H	5.46975800	-4.52165600	-3.25368300
C	6.69613300	-4.56618600	-1.48599900
H	7.28498600	-5.42012100	-1.83043800
C	6.96057300	-3.99443800	-0.24229000
H	7.75561200	-4.40895700	0.38093400
C	6.21702400	-2.89811400	0.21526700
C	6.51704100	-2.26294400	1.56809600
H	5.63077400	-1.67667200	1.85502700
C	6.75542800	-3.29344400	2.67851000
H	5.92569300	-4.01489900	2.74042100
H	6.84420700	-2.78997700	3.65452700
H	7.68514200	-3.86333500	2.52128200
C	7.69098200	-1.27697900	1.46047000
H	8.61347400	-1.79751600	1.15529300
H	7.88120700	-0.78241200	2.42693100
H	7.48411300	-0.49491100	0.71563100

C	3.80311800	-2.39099100	-2.74683100
H	3.10809900	-1.84823300	-2.08852400
C	4.36939800	-1.36299400	-3.73890700
H	5.08434200	-1.83784800	-4.43075700
H	4.89444400	-0.55235900	-3.21468700
H	3.56219800	-0.90958100	-4.33685300
C	2.98940400	-3.46657900	-3.47499800
H	3.58829300	-3.99816300	-4.23166600
H	2.13860500	-3.00604400	-4.00140400
H	2.58909800	-4.21428800	-2.77307000
C	4.74628300	2.59399100	-0.41412300
C	5.64757300	3.23074400	0.46965000
C	6.12292000	4.50790600	0.14303100
H	6.81342100	5.01682200	0.81941400
C	5.72665600	5.14027700	-1.03486500
H	6.10388600	6.13790300	-1.27357300
C	4.85587400	4.49496700	-1.91147100
H	4.55872600	4.99242500	-2.83775200
C	4.35774200	3.21747400	-1.62122900
C	3.40549700	2.53272300	-2.59386400
H	3.24701800	1.50643900	-2.23484700
C	3.99745200	2.43119300	-4.00602900
H	4.97804500	1.93148000	-3.99111700
H	4.13289700	3.42339300	-4.46608900
H	3.33009200	1.84987300	-4.66243500
C	2.02976400	3.21466300	-2.61452400
H	1.33875200	2.67214700	-3.27992800
H	2.09931800	4.25352900	-2.97634000
H	1.58038200	3.24053800	-1.61121700
C	6.07872400	2.56551400	1.77206100
H	5.75384100	1.51624500	1.72811200
C	7.60272000	2.55785200	1.94979500
H	8.00803100	3.57551600	2.06872000
H	7.88192400	1.98574600	2.84911800
H	8.10226000	2.09585500	1.08451100
C	5.37852700	3.20630600	2.98066900
H	5.65005700	2.68526900	3.91328700
H	5.66377300	4.26573200	3.08835800
H	4.28495400	3.16362800	2.87524200
H	1.60616600	0.04826900	-1.58086600
C	-0.08440800	-2.32321900	-1.57743100
H	0.73433700	-2.74379700	-0.98469800
H	-1.03972600	-2.75753700	-1.26592100
H	0.08360300	-2.52006300	-2.64399300

Cartesian coordinates of the optimized closed-shell singlet structure of **4** at B3LYP-D3BJ/def2-SVP,def2-TZVP level of theory (number of imaginary frequencies = 0). E = -3167.651399 au

Bi	-0.00007200	0.00002100	-0.74259400
C	-0.00004300	0.00014200	1.48261500
C	-1.21965300	-0.07735700	2.16215800
C	-1.23196400	-0.08644900	3.56649800
C	0.00001200	0.00001100	4.23962100
H	0.00002800	-0.00005000	5.33032800
C	1.23196400	0.08653100	3.56646400
C	1.21959000	0.07758000	2.16212000
C	-2.54665200	-0.23835500	4.31839400
H	-3.34833400	0.07510000	3.62918300
C	-2.64268700	0.64514000	5.56703600
H	-3.65257000	0.58715500	6.00317700
H	-2.43199900	1.69990800	5.33086200
H	-1.93206500	0.32806400	6.34696600
C	-2.80207800	-1.71579200	4.65986500
H	-2.79351800	-2.33902900	3.75354900
H	-3.77819000	-1.84418300	5.15582300
H	-2.02102000	-2.09929000	5.33651200
C	2.54671100	0.23828500	4.31829500
H	3.34829800	-0.07545900	3.62910300

C	2.80246300	1.71572900	4.65949300
H	2.02150000	2.09951300	5.33608800
H	2.79401900	2.33880500	3.75306700
H	3.77861300	1.84400100	5.15540600
C	2.64262900	-0.64501800	5.56707300
H	2.43171300	-1.69978100	5.33108000
H	1.93212600	-0.32767100	6.34700100
H	3.65255000	-0.58717300	6.00314200
C	-2.40911500	-0.09924900	1.25544900
C	-3.08216300	1.12209100	0.99302300
C	-4.09704300	1.14999700	0.02316300
C	-4.40780800	-0.02603000	-0.67699000
H	-5.19415500	0.00577700	-1.43443200
C	-3.75685400	-1.23191700	-0.43095000
C	-2.73486700	-1.28523800	0.54892000
C	-2.64790800	2.37216800	1.71465200
H	-1.61069100	2.62336400	1.43791600
H	-2.65421600	2.23459900	2.80516500
H	-3.28970100	3.22554700	1.46642400
C	-2.10553500	-2.61397600	0.89446600
H	-1.83951200	-3.17845900	-0.00877300
H	-2.82327000	-3.23644200	1.45559300
H	-1.20841100	-2.48932400	1.51136200
C	-4.84851800	2.40040700	-0.30322300
C	-4.98591200	4.52726100	-1.45047600
H	-4.54630400	5.28885700	-2.09662000
C	-4.25342200	3.37462600	-1.13745900
C	-6.27252000	4.71607900	-0.94748600
H	-6.83023500	5.62120100	-1.20105300
C	-6.84711500	3.75425300	-0.11797400
H	-7.85110100	3.91900000	0.27652600
C	-6.15035200	2.58527000	0.21713900
C	-2.94103300	2.40130500	-3.05263200
H	-1.93083500	2.18925300	-3.43820200
H	-3.48339700	2.99372200	-3.80757600
H	-3.46559900	1.44277500	-2.93580500
C	-2.86060700	3.15290600	-1.71523500
H	-2.30698200	2.49768500	-1.02622200
C	-2.04120400	4.43978500	-1.85192200
H	-1.00542000	4.19569600	-2.13160000
H	-2.01543500	5.00223300	-0.90564000
H	-2.44038700	5.10725300	-2.63262500
C	-6.75130100	1.56884100	1.18242600
H	-6.31608700	0.58940100	0.93944700
C	-6.33959900	1.89342700	2.62840300
H	-6.73397600	1.13774900	3.32744900
H	-6.72902300	2.87851800	2.93331600
H	-5.24573900	1.91765300	2.73619800
C	-8.27163000	1.42437700	1.06183000
H	-8.57654400	1.23030000	0.02174100
H	-8.80291700	2.32551400	1.40764800
H	-8.62218600	0.58408200	1.68148100
C	-4.17297500	-2.46661600	-1.16449100
C	-5.12818200	-3.32644100	-0.57329600
C	-5.51698300	-4.48056200	-1.26418100
H	-6.25817000	-5.15102700	-0.82441500
C	-4.97251600	-4.78503000	-2.51174000
H	-5.28473900	-5.69031800	-3.03833700
C	-4.03230400	-3.93306500	-3.08663200
H	-3.61351000	-4.17708000	-4.06561900
C	-3.61817000	-2.76654600	-2.42768300
C	-5.77108700	-2.97610300	0.76359000
H	-5.13128400	-2.22626800	1.25188300
C	-7.14269800	-2.31798200	0.54125500
H	-7.84027200	-3.02010600	0.05622900
H	-7.05806700	-1.43214600	-0.10526100
H	-7.58763000	-2.00018800	1.49823300
C	-5.87636200	-4.17200600	1.71777200
H	-6.58158000	-4.93369000	1.34879000
H	-6.23922600	-3.84326700	2.70463300

H	-4.89937100	-4.66005100	1.85863000
C	-2.60909900	-1.84327400	-3.09762500
H	-2.34878900	-1.05796800	-2.36977100
C	-3.22936600	-1.14137100	-4.31453500
H	-2.52376400	-0.41657300	-4.75074000
H	-4.14656500	-0.60042900	-4.03729100
H	-3.49540900	-1.87028800	-5.09719200
C	-1.30957800	-2.56733000	-3.47115700
H	-1.48097400	-3.34660400	-4.23071900
H	-0.85232900	-3.03935900	-2.58961700
H	-0.57642200	-1.85596100	-3.88388900
C	2.40904400	0.09938900	1.25540500
C	3.08192100	-1.12204400	0.99292300
C	4.09682700	-1.15003500	0.02309700
C	4.40779400	0.02599600	-0.67695900
H	5.19416500	-0.00586600	-1.43437500
C	3.75700400	1.23196200	-0.43085400
C	2.73499000	1.28535900	0.54895800
C	2.64745300	-2.37210900	1.71444900
H	1.61020900	-2.62312000	1.43764900
H	2.65373100	-2.23462600	2.80497600
H	3.28912900	-3.22556400	1.46617700
C	2.10574800	2.61415100	0.89443700
H	1.20884400	2.48961700	1.51167500
H	1.83937100	3.17842000	-0.00883600
H	2.82367800	3.23679800	1.45511800
C	4.84815800	-2.40051200	-0.30336700
C	4.25295300	-3.37462800	-1.13765100
C	4.98535800	-4.52727700	-1.45079900
H	4.54567600	-5.28880000	-2.09698100
C	6.27198600	-4.71621200	-0.94789600
H	6.82963500	-5.62133900	-1.20159400
C	6.84668000	-3.75449900	-0.11832800
H	7.85068300	-3.91932800	0.27610200
C	6.14999900	-2.58550400	0.21692700
C	6.75105500	-1.56921400	1.18229400
H	6.31588600	-0.58972300	0.93945400
C	6.33935800	-1.89397700	2.62823500
H	5.24549700	-1.91813900	2.73603400
H	6.73379900	-1.13843600	3.32739600
H	6.72870200	-2.87915000	2.93299200
C	8.27137900	-1.42479600	1.06165300
H	8.80266900	-2.32599000	1.40731300
H	8.62199400	-0.58460100	1.68140600
H	8.57623700	-1.23056800	0.02157600
C	2.86010000	-3.15276100	-1.71527100
H	2.30651400	-2.49778800	-1.02598900
C	2.94035100	-2.40072300	-3.05242900
H	3.48268400	-2.99285300	-3.80762100
H	3.46485100	-1.44218400	-2.93535000
H	1.93009600	-2.18862300	-3.43782700
C	2.04072600	-4.43962300	-1.85231800
H	2.43987000	-5.10680800	-2.63328500
H	1.00491000	-4.19547200	-2.13182800
H	2.01504900	-5.00240000	-0.90622900
C	4.17342200	2.46661300	-1.16431200
C	5.12900200	3.32604500	-0.57315600
C	5.51827500	4.47998700	-1.26408000
H	6.25977100	5.15013700	-0.82435200
C	4.97389300	4.78466600	-2.51162100
H	5.28650700	5.68979500	-3.03826300
C	4.03325300	3.93312100	-3.08644200
H	3.61449600	4.17732600	-4.06540000
C	3.61863600	2.76680200	-2.42745100
C	2.60898400	1.84404400	-3.09723800
H	2.34856000	1.05875300	-2.36941000
C	3.22852700	1.14204600	-4.31445100
H	4.14566600	0.60079200	-4.03761400
H	3.49447700	1.87092400	-5.09717700
H	2.52251900	0.41749700	-4.75041400

C	1.30966400	2.56877200	-3.47015900
H	0.57607200	1.85788400	-3.88293700
H	1.48118300	3.34827700	-4.22945300
H	0.85287800	3.04065100	-2.58829800
C	5.77175400	2.97550900	0.76375100
H	5.13177400	2.22576100	1.25194500
C	7.14327900	2.31718900	0.54150500
H	7.84096900	3.01920100	0.05648500
H	7.58812700	1.99937000	1.49851200
H	7.05856500	1.43133800	-0.10498100
C	5.87713300	4.17134800	1.71800800
H	6.23993300	3.84250700	2.70485900
H	6.58244500	4.93297600	1.34909000
H	4.90019200	4.65949000	1.85886700

Cartesian coordinates of the optimized open-shell singlet structure of **4** at B3LYP-D3BJ/def2-SVP,def2-TZVP level of theory (number of imaginary frequencies = 0). E = -3167.661462 au

Bi	-0.00412700	-0.01109800	-1.01155900
C	0.00227800	0.04726400	1.25178500
C	-1.21932100	0.06207600	1.94497800
C	-1.23207600	0.12539000	3.35228400
C	-0.00292000	0.15669100	4.02385900
H	-0.00164400	0.20148500	5.11474300
C	1.22970100	0.13481000	3.35327300
C	1.22470500	0.07964800	1.94749600
C	-2.55231500	0.12408400	4.11317400
H	-3.32634600	0.47790600	3.41390600
C	-2.55784500	1.07200200	5.31794100
H	-3.56848200	1.13409000	5.75152700
H	-2.24595000	2.08857300	5.03155300
H	-1.88228100	0.72579400	6.11656800
C	-2.94458500	-1.30340800	4.52785700
H	-3.01741000	-1.96649800	3.65438000
H	-3.91967500	-1.30931700	5.04181000
H	-2.19450500	-1.73135900	5.21306600
C	2.53420300	0.17486700	4.13915800
H	3.35529500	0.16072500	3.40524400
C	2.66766600	1.46863700	4.95659700
H	1.89020200	1.53314500	5.73499100
H	2.57206700	2.35908800	4.31700500
H	3.64748600	1.51365700	5.45887800
C	2.70081900	-1.06017900	5.03735100
H	2.63705200	-1.99205500	4.45584200
H	1.92119400	-1.09767100	5.81543800
H	3.67931800	-1.04465400	5.54377300
C	-2.46913000	0.02525800	1.12605000
C	-3.08036800	1.25176100	0.77051700
C	-4.18272300	1.23793500	-0.10082700
C	-4.65670700	0.00940300	-0.58259400
H	-5.51172300	0.00385800	-1.26245200
C	-4.07248900	-1.20294200	-0.22087000
C	-2.95554800	-1.20584500	0.64123300
C	-2.50475000	2.54452800	1.29343700
H	-1.48335100	2.69803300	0.90818000
H	-2.41908200	2.53162200	2.38990800
H	-3.11564600	3.40684600	1.00150700
C	-2.31174000	-2.51962400	1.01075500
H	-1.96389800	-3.05125100	0.11204300
H	-3.03745300	-3.18843000	1.49915300
H	-1.45584300	-2.38255400	1.68137400
C	-4.86064600	2.49573000	-0.54037400
C	-4.92952500	4.45803800	-1.95805700
H	-4.48916100	5.08616900	-2.73404500
C	-4.26555400	3.29616600	-1.54379900
C	-6.14980300	4.82491200	-1.39187900
H	-6.65486300	5.73471200	-1.72613300
C	-6.72439100	4.03534300	-0.39723000

H	-7.67538000	4.33894200	0.04332400
C	-6.09491600	2.86329700	0.04415600
C	-3.19523100	1.92849200	-3.36863900
H	-2.23962100	1.57853700	-3.79318400
H	-3.76208100	2.43353400	-4.16761300
H	-3.76737600	1.04494300	-3.05380900
C	-2.94780000	2.87826400	-2.18658200
H	-2.38334300	2.30299900	-1.43818100
C	-2.05926200	4.05544100	-2.59980700
H	-1.06967000	3.68811000	-2.91168900
H	-1.91241600	4.76070000	-1.76701300
H	-2.48114900	4.61681000	-3.44891800
C	-6.69034300	2.03904400	1.18124600
H	-6.36929300	0.99853800	1.03373700
C	-6.11663700	2.49489500	2.53358400
H	-6.51075500	1.87478600	3.35540200
H	-6.38442000	3.54453400	2.73757500
H	-5.02001300	2.41962700	2.54805200
C	-8.22199000	2.03427900	1.20717000
H	-8.64067600	1.74754400	0.22997800
H	-8.63596900	3.01910200	1.47699200
H	-8.58490200	1.31395600	1.95710800
C	-4.62989000	-2.48929200	-0.73881900
C	-5.59324000	-3.18289700	0.02990800
C	-6.11088100	-4.38663200	-0.46413200
H	-6.86128100	-4.93097700	0.11299500
C	-5.68083500	-4.90146900	-1.68741300
H	-6.09323200	-5.84282200	-2.05922000
C	-4.72487300	-4.21568900	-2.43386100
H	-4.39291800	-4.62648600	-3.39035600
C	-4.18463900	-3.00603500	-1.97464300
C	-6.09420600	-2.60815600	1.34975000
H	-5.36262800	-1.85493100	1.67784100
C	-7.43139700	-1.87773100	1.14598200
H	-8.21546700	-2.57918800	0.81703200
H	-7.34252600	-1.09382700	0.37943900
H	-7.76844800	-1.40262000	2.08175100
C	-6.19491900	-3.65469300	2.46616500
H	-6.97671000	-4.40360500	2.26186700
H	-6.44908500	-3.17035000	3.42250600
H	-5.24254600	-4.19129300	2.59932100
C	-3.15136000	-2.26865100	-2.81665000
H	-2.81365100	-1.39655000	-2.23673200
C	-3.77447800	-1.73700000	-4.11552900
H	-3.04398100	-1.13995400	-4.68457900
H	-4.64575000	-1.09843400	-3.90489300
H	-4.11434400	-2.56247800	-4.76203500
C	-1.91049900	-3.12550100	-3.10180400
H	-2.15481500	-4.01106200	-3.71019000
H	-1.44311300	-3.47596600	-2.16913000
H	-1.15753000	-2.54021800	-3.65390600
C	2.47335800	0.04398400	1.12613500
C	3.02555000	-1.20784900	0.77582100
C	4.13985000	-1.24208100	-0.08376100
C	4.66149500	-0.03880400	-0.57104300
H	5.51960900	-0.07167900	-1.24608900
C	4.12327600	1.20291900	-0.22465100
C	3.01394100	1.25456300	0.64122600
C	2.38458600	-2.47626400	1.28197600
H	1.39781700	-2.62457200	0.81191300
H	2.20307800	-2.43095200	2.36437600
H	3.00080500	-3.35645400	1.06298800
C	2.39903200	2.56985100	1.05073800
H	1.32687300	2.59998900	0.80294000
H	2.89426100	3.41626500	0.56003600
H	2.46826300	2.71457500	2.13983400
C	4.78260100	-2.52759100	-0.49451300
C	4.19262500	-3.31021200	-1.51414800
C	4.82105000	-4.50301700	-1.89464500
H	4.38360000	-5.11897300	-2.68196000

C	6.00269300	-4.91568000	-1.28016300
H	6.48035700	-5.84940600	-1.58766000
C	6.57488100	-4.14034200	-0.27285400
H	7.49689000	-4.47838400	0.20275700
C	5.98043900	-2.93840100	0.13560500
C	6.57569100	-2.12224000	1.27919600
H	6.31016200	-1.07048200	1.10189500
C	5.93809700	-2.52358000	2.62023200
H	4.84750700	-2.38899000	2.60132600
H	6.34030000	-1.91093400	3.44372700
H	6.14367800	-3.58207400	2.84916800
C	8.10425300	-2.18940100	1.35645100
H	8.46150800	-3.18702200	1.65844600
H	8.47636500	-1.47212600	2.10480400
H	8.56812200	-1.94235700	0.38889500
C	2.92104600	-2.83880600	-2.20999400
H	2.35417500	-2.23465000	-1.48605600
C	3.25472900	-1.90891600	-3.38716900
H	3.82590700	-2.44539000	-4.16222500
H	3.85529800	-1.04964300	-3.05734100
H	2.33224600	-1.51879200	-3.84832700
C	1.99774400	-3.97786000	-2.65212200
H	2.42975200	-4.56656000	-3.47727400
H	1.04110500	-3.56862300	-3.01044900
H	1.78221500	-4.66711600	-1.82084400
C	4.73867300	2.44353600	-0.78765900
C	5.77098900	3.09309700	-0.07336400
C	6.35190400	4.24273000	-0.62367500
H	7.15541100	4.75286500	-0.08795700
C	5.91697900	4.74707000	-1.84939200
H	6.37827400	5.64655300	-2.26484800
C	4.89248100	4.10481200	-2.54233500
H	4.55461800	4.50917400	-3.49956000
C	4.29001300	2.94814900	-2.02774300
C	3.16247100	2.27286300	-2.79817100
H	2.87749500	1.36746900	-2.24224100
C	3.61270200	1.82219100	-4.19434900
H	4.49649100	1.16875500	-4.13477700
H	3.87342200	2.68110700	-4.83376700
H	2.80901400	1.26192800	-4.69862700
C	1.91499200	3.16547800	-2.86993200
H	1.09270000	2.64064800	-3.38259500
H	2.11609000	4.09764600	-3.42252200
H	1.56270300	3.43850600	-1.86391500
C	6.27047400	2.53482100	1.25369400
H	5.51976500	1.81299800	1.60771200
C	7.58322100	1.76007000	1.05700100
H	8.38459900	2.42681100	0.69873900
H	7.91723300	1.30299600	2.00279600
H	7.46188100	0.95622800	0.31560100
C	6.40914600	3.60880200	2.33985200
H	6.67252500	3.14687100	3.30493800
H	7.19944900	4.33838500	2.10091600
H	5.46763700	4.16427900	2.47229700

Cartesian coordinates of the optimized triplet structure of **4** at B3LYP-D3BJ/def2-SVP,def2-TZVP level of theory (number of imaginary frequencies = 0). E = -3167.668743 au

Bi	-0.01255100	0.03340200	-1.05872500
C	-0.00040100	0.00084600	1.21389900
C	1.22369700	-0.00913700	1.90931800
C	1.23559700	-0.05308600	3.31697900
C	0.00783300	-0.04263800	3.99312800
H	0.01177900	-0.05944000	5.08413500
C	-1.22420900	-0.01223400	3.32569600
C	-1.21990300	-0.01209000	1.91739900
C	2.55299500	-0.07133600	4.08278900
H	3.33085200	-0.40168600	3.37708400

C	2.55191100	-1.05978500	5.25522700
H	3.55872300	-1.13184200	5.69623600
H	2.24908800	-2.06766000	4.93081200
H	1.86612800	-0.74523300	6.05824800
C	2.94019300	1.34196500	4.54686200
H	3.01895100	2.03310800	3.69565200
H	3.91144800	1.33170100	5.06787300
H	2.18539700	1.74729700	5.24071400
C	-2.53841300	-0.02104900	4.09742700
H	-3.31462100	0.35808100	3.41451100
C	-2.94259600	-1.45345500	4.48227500
H	-2.18990100	-1.90731200	5.14773300
H	-3.03523700	-2.09429700	3.59418300
H	-3.91116100	-1.45984000	5.00835900
C	-2.52168400	0.89825100	5.32448300
H	-2.20278800	1.91819700	5.05846600
H	-1.84127800	0.52705700	6.10763300
H	-3.52712700	0.96020700	5.77002600
C	2.48473700	0.01443500	1.10737100
C	3.08212100	-1.21685900	0.75094900
C	4.20108100	-1.21182800	-0.09920500
C	4.69574100	0.01182000	-0.57026700
H	5.55843100	0.00923200	-1.24019200
C	4.12259900	1.22953000	-0.20613300
C	2.99984200	1.24121800	0.64725500
C	2.47380200	-2.50551700	1.24661600
H	1.46902500	-2.65105100	0.81661400
H	2.33901500	-2.49258100	2.33802700
H	3.08927000	-3.37300400	0.98089800
C	2.36077500	2.55937400	1.00775500
H	1.94809700	3.05122400	0.11203700
H	3.10403100	3.25658800	1.42374600
H	1.54675500	2.43744200	1.73153800
C	4.87198800	-2.47791900	-0.52528100
C	4.96199100	-4.43130200	-1.95518400
H	4.54307700	-5.03704500	-2.76214100
C	4.31181500	-3.25191100	-1.56552500
C	6.13727400	-4.83949600	-1.32895700
H	6.63268900	-5.76191600	-1.64236900
C	6.68434000	-4.06941100	-0.30198200
H	7.60474900	-4.40114700	0.18129500
C	6.06868000	-2.88221500	0.11451800
C	3.34390900	-2.35264400	-3.71386100
H	2.43150400	-1.97271700	-4.20239100
H	3.73677500	-3.18007800	-4.32711100
H	4.09228700	-1.54652600	-3.71801400
C	3.03972400	-2.81592700	-2.28177600
H	2.63636500	-1.94555400	-1.74432500
C	1.95312200	-3.89932700	-2.26401500
H	1.02053400	-3.51471900	-2.70573200
H	1.73197700	-4.22595900	-1.23667600
H	2.25171900	-4.78838500	-2.84252000
C	6.63879600	-2.07308200	1.27554300
H	6.34571700	-1.02552700	1.11771200
C	6.00867100	-2.51782400	2.60625100
H	6.38731000	-1.90714600	3.44228200
H	6.24679600	-3.57312100	2.81775000
H	4.91435300	-2.41880400	2.58343800
C	8.16844600	-2.10179300	1.35584200
H	8.62765600	-1.81982000	0.39567900
H	8.55110100	-3.09658300	1.63514800
H	8.52018300	-1.39309500	2.12203500
C	4.69312900	2.50954400	-0.72518500
C	5.73102300	3.14816400	-0.01090600
C	6.26801600	4.33737500	-0.52206600
H	7.07671600	4.84027300	0.01260900
C	5.78136300	4.89000700	-1.70611300
H	6.21043800	5.81798700	-2.09246300
C	4.74344600	4.26254200	-2.39448200
H	4.36614400	4.70642200	-3.31823900

C	4.18194500	3.07127500	-1.91635600
C	6.27885300	2.54120000	1.27526200
H	5.54825200	1.79450600	1.62062600
C	7.59576800	1.79533600	1.00798000
H	8.37516800	2.48874700	0.65185600
H	7.46362600	1.01897700	0.23991000
H	7.96608700	1.30754900	1.92434600
C	6.43833700	3.56953600	2.40220800
H	7.21544300	4.31626300	2.17337700
H	6.73200500	3.06906000	3.33883100
H	5.49678700	4.11035100	2.58584600
C	3.07129300	2.37248500	-2.68934000
H	2.60151600	1.64784400	-2.00660300
C	3.65089000	1.56708300	-3.86159500
H	2.85958100	1.00216900	-4.38067600
H	4.40553400	0.84831200	-3.50962900
H	4.13579300	2.23190900	-4.59516200
C	1.96512000	3.32450700	-3.15845400
H	2.32292700	4.03325100	-3.92225000
H	1.55686700	3.91032300	-2.32028500
H	1.13690600	2.75228000	-3.60669700
C	-2.48391600	-0.01716600	1.12012200
C	-3.08925200	1.22119800	0.80339400
C	-4.20299000	1.23650700	-0.05375100
C	-4.69324300	0.02388000	-0.55794100
H	-5.55519600	0.04095400	-1.22877200
C	-4.11506200	-1.20124300	-0.22978600
C	-2.98895100	-1.23211000	0.61859900
C	-2.49500000	2.49689900	1.34786100
H	-1.47992100	2.65654800	0.94819700
H	-2.38811700	2.45438000	2.44187900
H	-3.10619500	3.37022300	1.09119000
C	-2.33966900	-2.55647900	0.93720700
H	-1.51106900	-2.44829200	1.64633400
H	-1.94663000	-3.02800500	0.02277300
H	-3.07153300	-3.26475100	1.35448600
C	-4.87218700	2.51095100	-0.45671600
C	-4.28151000	3.32511300	-1.45160800
C	-4.93465200	4.50539200	-1.82944600
H	-4.49751100	5.14439500	-2.59845500
C	-6.14053700	4.87663400	-1.23592600
H	-6.63737500	5.80091200	-1.54154200
C	-6.71169000	4.07237000	-0.25100500
H	-7.65187900	4.37888100	0.21026500
C	-6.09261900	2.88183700	0.15410700
C	-6.68391200	2.03828100	1.27918600
H	-6.37969200	0.99735900	1.10144700
C	-6.08542600	2.45229100	2.63422300
H	-4.99001000	2.36032300	2.63188500
H	-6.47788100	1.81799000	3.44594200
H	-6.33465600	3.50040200	2.86752900
C	-8.21497900	2.05428600	1.32640400
H	-8.61116300	3.03782900	1.62603800
H	-8.57788200	1.32079400	2.06349000
H	-8.65099700	1.79758100	0.34844900
C	-2.98214200	2.90117900	-2.12709700
H	-2.42155400	2.28846600	-1.40600300
C	-3.26803700	1.99705000	-3.33583000
H	-3.83466800	2.54117000	-4.10889200
H	-3.85547900	1.11673900	-3.04087200
H	-2.32732900	1.63929800	-3.78634700
C	-2.07387700	4.07225300	-2.51413500
H	-2.49804100	4.67419000	-3.33374500
H	-1.09911300	3.69509900	-2.85993200
H	-1.89522000	4.74290100	-1.65919400
C	-4.68460700	-2.47061400	-0.77574900
C	-5.65632600	-3.17082600	-0.02381300
C	-6.18530300	-4.35832300	-0.54456500
H	-6.94234200	-4.90731400	0.01938800
C	-5.75757100	-4.85146200	-1.77758400

H	-6.17877000	-5.78036500	-2.17027700
C	-4.79233000	-4.16012400	-2.50686700
H	-4.46186200	-4.55421600	-3.47091900
C	-4.24096200	-2.96601800	-2.02097900
C	-3.19652300	-2.22218200	-2.84346600
H	-2.85552500	-1.36269800	-2.24740600
C	-3.80587700	-1.66443900	-4.13785800
H	-4.67447100	-1.02373500	-3.92280400
H	-4.14605200	-2.47603000	-4.80160000
H	-3.06701400	-1.06234600	-4.69062700
C	-1.96041700	-3.08426400	-3.13505000
H	-1.19492700	-2.49367400	-3.66421000
H	-2.20597600	-3.95273800	-3.76715500
H	-1.50849700	-3.46322500	-2.20579600
C	-6.15099700	-2.62116500	1.30902500
H	-5.41219000	-1.88191800	1.65233600
C	-7.48222000	-1.87501200	1.12442600
H	-8.27267400	-2.56173700	0.77996300
H	-7.81420100	-1.41949900	2.07169400
H	-7.38752400	-1.07373800	0.37674100
C	-6.25817900	-3.69163400	2.40192100
H	-6.50806100	-3.22700400	3.36913400
H	-7.04535200	-4.43039900	2.18186000
H	-5.30935800	-4.23754500	2.52202900

Cartesian coordinates of the optimized triplet structure of **4** at B3LYP-D3BJ/def2-TZVP level of theory (number of imaginary frequencies = 0). E = -3170.831740 au

Bi	0.00224800	0.01322900	-1.09629500
C	0.00054200	-0.00620100	1.18032000
C	1.21559500	-0.01017200	1.87618800
C	1.22170200	-0.04920200	3.27571700
C	-0.00162200	-0.03634000	3.94274800
H	-0.00225600	-0.04806400	5.02468000
C	-1.22381900	-0.00873100	3.27455600
C	-1.21532700	-0.01702900	1.87418100
C	2.52863000	-0.06842000	4.04989400
H	3.31460700	-0.35082500	3.34738800
C	2.53438700	-1.10075900	5.18075500
H	3.52807900	-1.16407600	5.62817800
H	2.26381800	-2.09216700	4.81397600
H	1.83415200	-0.83558700	5.97515500
C	2.87658100	1.32887300	4.57932500
H	2.94913600	2.05119900	3.76650600
H	3.83294700	1.31436200	5.10672600
H	2.11129200	1.68112600	5.27468600
C	-2.53258600	-0.00628700	4.04618800
H	-3.31506500	0.30373300	3.35149100
C	-2.89140700	-1.41775900	4.52880100
H	-2.12874400	-1.79907200	5.21162200
H	-2.97040500	-2.11194700	3.69247400
H	-3.84753800	-1.41309000	5.05679300
C	-2.53536100	0.98855100	5.21006800
H	-2.25649700	1.98949000	4.87688200
H	-1.84046900	0.69262100	5.99827800
H	-3.53043300	1.04418300	5.65540200
C	2.47967300	0.02050600	1.08561100
C	3.08661700	-1.19888800	0.73475300
C	4.21421900	-1.18278800	-0.08926500
C	4.71256000	0.03875300	-0.53596700
H	5.58289000	0.04432000	-1.17990000
C	4.12789000	1.24487000	-0.18115200
C	2.99328600	1.24538800	0.64249100
C	2.48682200	-2.49136500	1.22243000
H	1.46913400	-2.61320100	0.84507600
H	2.41418100	-2.50788200	2.31079600
H	3.07510600	-3.34756900	0.90452300
C	2.33379700	2.55269000	0.99347200
H	1.85667300	2.99305900	0.11397400

H	3.06812000	3.27901100	1.34366400
H	1.57032500	2.42778500	1.75597200
C	4.88898100	-2.44246900	-0.51576900
C	5.03351700	-4.33511700	-1.99849200
H	4.67078500	-4.88948300	-2.85476600
C	4.40384100	-3.14771000	-1.62953800
C	6.11880500	-4.81670100	-1.28415800
H	6.59548900	-5.74268100	-1.58033100
C	6.59848800	-4.10790400	-0.19265500
H	7.44961100	-4.49055400	0.35477700
C	6.00024500	-2.91439900	0.20537300
C	3.61517400	-2.33494000	-3.88308800
H	2.77232600	-1.90066600	-4.42501800
H	3.92286100	-3.23936900	-4.41184900
H	4.44377500	-1.62714300	-3.92097200
C	3.21905400	-2.64255100	-2.43494700
H	2.88742500	-1.70622200	-1.98622700
C	2.03481600	-3.61425100	-2.38379100
H	1.17592400	-3.19620600	-2.91196100
H	1.73242700	-3.81389900	-1.35566400
H	2.28316700	-4.56916200	-2.85166900
C	6.51609900	-2.16916800	1.42581400
H	6.12793200	-1.15211700	1.37642100
C	5.97523300	-2.79735400	2.71741800
H	6.31508200	-2.23557900	3.59054800
H	6.32094300	-3.82828500	2.82173400
H	4.88552400	-2.80809800	2.72422500
C	8.04292600	-2.06691400	1.46351200
H	8.43233100	-1.63444900	0.54079400
H	8.51396400	-3.04159200	1.60312500
H	8.35582500	-1.43139900	2.29406500
C	4.70897200	2.52091200	-0.68821100
C	5.74136600	3.14530600	0.03036100
C	6.30036600	4.31552100	-0.47961400
H	7.10180500	4.80635400	0.05690100
C	5.84059400	4.86368300	-1.66713500
H	6.28536000	5.77293800	-2.05163200
C	4.80548900	4.25209700	-2.35851600
H	4.44875000	4.69330500	-3.27987100
C	4.22219200	3.08057600	-1.88120700
C	6.25877700	2.54845500	1.32812800
H	5.52591400	1.81374200	1.66390300
C	7.57829200	1.80134000	1.09732200
H	8.35482800	2.48494200	0.74704700
H	7.45930500	1.01833900	0.34808000
H	7.92731500	1.33602600	2.02172600
C	6.40033200	3.58623200	2.44537000
H	7.17743400	4.31989300	2.22366600
H	6.67108700	3.09561300	3.38245100
H	5.46529100	4.12622600	2.60276600
C	3.10757900	2.40182300	-2.65720200
H	2.61149900	1.70734100	-1.97736100
C	3.67754500	1.56581800	-3.80901700
H	2.88042900	1.02918900	-4.32825200
H	4.39392700	0.83196700	-3.43995000
H	4.18981600	2.20192900	-4.53431300
C	2.03927900	3.37568800	-3.15859700
H	2.42838700	4.05416500	-3.91987500
H	1.63894600	3.97972100	-2.34296100
H	1.21154300	2.82264700	-3.60706800
C	-2.47853800	-0.03258000	1.08191500
C	-3.08936400	1.19207300	0.75836800
C	-4.21580500	1.19087800	-0.06764700
C	-4.71033400	-0.02239300	-0.54083200
H	-5.58057300	-0.01691900	-1.18501800
C	-4.12333000	-1.23438400	-0.21065200
C	-2.98799900	-1.24918000	0.61161400
C	-2.49134800	2.47645400	1.26880100
H	-1.49542300	2.63364200	0.84770100
H	-2.36646100	2.45303900	2.35189100

H	-3.10874400	3.33243200	1.01086900
C	-2.31947200	-2.56025500	0.93047100
H	-1.58072800	-2.45520600	1.71992100
H	-1.80747800	-2.95601300	0.04911900
H	-3.05178100	-3.31125800	1.22768200
C	-4.89480100	2.45621500	-0.46976100
C	-4.37939000	3.21281300	-1.53492300
C	-5.02401400	4.39582700	-1.89234900
H	-4.64048300	4.98709100	-2.71414200
C	-6.15089300	4.82536900	-1.21052200
H	-6.63963700	5.74806000	-1.49717700
C	-6.65606100	4.06998400	-0.16225900
H	-7.53768400	4.41474900	0.36099500
C	-6.04512900	2.87844800	0.22164600
C	-6.58132100	2.08298100	1.40126300
H	-6.22964300	1.05753900	1.29047600
C	-6.00552800	2.61648600	2.72045800
H	-4.91638300	2.58335700	2.71755900
H	-6.36112900	2.02102500	3.56453300
H	-6.30926700	3.65301700	2.88358500
C	-8.10956200	2.03053900	1.45418500
H	-8.54556700	3.00760700	1.67026700
H	-8.43243700	1.34914400	2.24337100
H	-8.52655800	1.67692600	0.51007000
C	-3.15290300	2.76063300	-2.30833500
H	-2.77244200	1.85819300	-1.82998200
C	-3.51328300	2.38793500	-3.75070500
H	-3.87710700	3.25626200	-4.30405700
H	-4.29208700	1.62532900	-3.77451800
H	-2.63852000	1.99688400	-4.27502900
C	-2.02790000	3.80026400	-2.27253100
H	-2.31430500	4.71874700	-2.78876900
H	-1.13388300	3.40869700	-2.76115900
H	-1.76448600	4.06107900	-1.24716600
C	-4.70520600	-2.50192200	-0.73787700
C	-5.72790700	-3.14378700	-0.02016200
C	-6.28496000	-4.30881400	-0.54351100
H	-7.07869200	-4.81260800	-0.00742700
C	-5.83378100	-4.83519700	-1.74429600
H	-6.27693100	-5.74084700	-2.13902000
C	-4.81061900	-4.20510100	-2.43633300
H	-4.46173200	-4.62746300	-3.36963700
C	-4.22937600	-3.03785700	-1.94565500
C	-3.12937600	-2.34113500	-2.72624800
H	-2.66108600	-1.61682200	-2.05819600
C	-3.71508300	-1.55102000	-3.90226800
H	-4.46157100	-0.83559100	-3.55669200
H	-4.19698100	-2.21984200	-4.61887800
H	-2.93166900	-0.99805000	-4.42503900
C	-2.02882600	-3.29527000	-3.19591700
H	-1.21775400	-2.73111500	-3.66062900
H	-2.39565100	-4.00953700	-3.93520500
H	-1.61128200	-3.86051000	-2.36142100
C	-6.23870100	-2.57140900	1.29122500
H	-5.51750600	-1.82438500	1.62450500
C	-7.57753600	-1.85139600	1.08720700
H	-8.34506100	-2.54856000	0.74395100
H	-7.92159300	-1.40074600	2.02072900
H	-7.48747300	-1.06055000	0.34199700
C	-6.34043500	-3.62419500	2.39883600
H	-6.61172400	-3.15082600	3.34461300
H	-7.10118900	-4.37505700	2.17800600
H	-5.39011400	-4.14178500	2.53894800

VI. References

[1] G. B. Deacon, G.J.Farquharson, "Synthesis of perbromobenzoic acids and perbromobenzenes from aromatic carboxylic acids by permercuration and bromodemercuration" *Aust. J. Chem*. **1977**, *30*, 293

[2] K. V. Baker, J. M. brown, N. Hughes, A. J. Skarnulis, A. Sexton, "Mechanical activation of magnesium turnings for the preparation of reactive Grignard reagents" *J. Org. Chem*. **1991**, *56*, 698.

[3] G. M. Sheldrick, "Phase annealing in SHELX-90: direct methods for larger structures" *Acta Cryst*. **1990**, *A46*, 467

[4] G. M. Sheldrick, SHELXL-2017, "Program for the Refinement of Crystal Structures University of Göttingen, Göttingen (Germany) **2017**. (see also: G.M. Sheldrick "Crystal structure refinement with SHELXL", *Acta Cryst.,***2015** *C71*, 3-8)

[5] C. B. Hübschle, G. M. Sheldrick, B. Dittrich, shelXle, "A Qt GUI for SHELXL" *J. Appl. Cryst.* **2011**, *44*, 1281-1284

[6] L. Falivene, Z. Cao, A. Petta, L. Serra, A. Poater, R. Oliva, V. Scarano, L. Cavallo, "Towards the online computer-aided design of catalytic pockets" *Nat. Chem.* **2019**, *11*, 872–879.

[7] M. J. Frisch, G. W. Trucks, H. B. Schlegel, G. E. Scuseria, M. A. Robb, J. R. Cheeseman, G. Scalmani, V. Barone, G. A. Petersson, H. Nakatsuji, X. Li, M. Caricato, A. V. Marenich, J. Bloino, B. G. Janesko, R. Gomperts, B. Mennucci, H. P. Hratchian, J. V. Ortiz, A. F. Izmaylov, J. L. Sonnenberg, D. Williams-Young, F. Ding, F. Lipparini, F. Egidi, J. Goings, B. Peng, A. Petrone, T. Henderson, D. Ranasinghe, V. G. Zakrzewski, J. Gao, N. Rega, G. Zheng, W. Liang, M. Hada, M. Ehara, K. Toyota, R. Fukuda, J. Hasegawa, M. Ishida, T. Nakajima, Y. Honda, O. Kitao, H. Nakai, T. Vreven, K. Throssell, J. A. Montgomery, Jr.; , J. E. Peralta, F. Ogliaro, M. J. Bearpark, J. J. Heyd, E. N. Brothers, K. N. Kudin, V. N. Staroverov, T. A. Keith, R. Kobayashi, J. Normand, K. Raghavachari, A. P. Rendell, J. C. Burant, S. S. Iyengar, J. Tomasi, M. Cossi, J. M. Millam, M. Klene, C. Adamo, R. Cammi, J. W. Ochterski, R. L. Martin, K. Morokuma, O. Farkas, J. B. Foresman, D. J. Fox, "Gaussian 16, Revision A.03" Wallingford CT, **2016**.

[8] F. Neese, "Software update: The ORCA program system—Version 5.0" *WIREs Comput. Mol. Sci.* **2022**, *12*, e1606.

[9] A. D. Becke, "Density-functional exchange-energy approximation with correct asymptotic behavior" *Phys. Rev. A* **1988**, *38*, 3098-3100.

[10] C. Lee, W. Yang, R. G. Parr, "Development of the Colle-Salvetti correlation-energy formula into a functional of the electron density" *Phys. Rev. B* **1988**, *37*, 785-789.

[11] B. Miehlich, A. Savin, H. Stoll, H. Preuss, "Results obtained with the correlation energy density functionals of Becke and Lee, Yang and Parr" *Chem. Phys. Lett.* **1989**, *157*, 200-206.

[12] S. Grimme, S. Ehrlich, L. Goerigk, "Effect of the damping function in dispersion corrected density functional theory" *J. Comp. Chem.* **2011**, *32*, 1456-1465.

[13] F. Weigend, R. Ahlrichs, "Balanced basis sets of split valence, triple zeta valence and quadruple zeta valence quality for H to Rn: Design and assessment of accuracy" *Phys. Chem. Chem. Phys.* **2005**, *7*, 3297-3305.

[14] F. Weigend, "Accurate Coulomb-fitting basis sets for H to Rn" *Phys. Chem. Chem. Phys.* **2006**, *8*, 1057-1065.

[15] B. P. Pritchard, D. Altarawy, B. Didier, T. D. Gibson, T. L. Windus, "New Basis Set Exchange: An Open, Up-to-Date Resource for the Molecular Sciences Community" *J. Chem. Inf. Model.* **2019**, *59*, 4814-4820.

[16] C. Angeli, R. Cimiraglia, S. Evangelisti, T. Leininger, J.-P. Malrieu, "Introduction of n-electron valence states for multireference perturbation theory" *J. Chem. Phys.* **2001**, *114*, 10252-10264.

[17] W. Kutzelnigg, W. Liu, “Quasirelativistic theory equivalent to fully relativistic theory” *J. Chem. Phys.* **2005**, *123*, 241102.
[18] P. Pollak, F. Weigend, “Segmented Contracted Error-Consistent Basis Sets of Double- and Triple-ζ Valence Quality for One- and Two-Component Relativistic All-Electron Calculations” *J. Chem. Theory Comput.* **2017**, *13*, 3696-3705.
[19] Y. J. Franzke, R. Treß, T. M. Pazdera, F. Weigend, 2Error-consistent segmented contracted all-electron relativistic basis sets of double- and triple-zeta quality for NMR shielding constants” *Phys. Chem. Chem. Phys.* **2019**, *21*, 16658-16664.
[20] B. A. Heß, C. M. Marian, U. Wahlgren, O. Gropen, “A mean-field spin-orbit method applicable to correlated wavefunctions” *Chem. Phys. Lett.* **1996**, *251*, 365-371.
[21] F. Neese, “Efficient and accurate approximations to the molecular spin-orbit coupling operator and their use in molecular g-tensor calculations” *J. Chem. Phys.* **2005**, *122*, 34107.
